# Genome-Wide Linkage and Association Mapping of Halo Blight Resistance in Common Bean to Race 6 of the Globally Important Bacterial Pathogen

**DOI:** 10.3389/fpls.2017.01170

**Published:** 2017-07-07

**Authors:** Andrew J. Tock, Deidré Fourie, Peter G. Walley, Eric B. Holub, Alvaro Soler, Karen A. Cichy, Marcial A. Pastor-Corrales, Qijian Song, Timothy G. Porch, John P. Hart, Renato C. C. Vasconcellos, Joana G. Vicente, Guy C. Barker, Phillip N. Miklas

**Affiliations:** ^1^School of Life Sciences, Faculty of Science, University of WarwickWellesbourne, United Kingdom; ^2^Department of Plant Sciences, Faculty of Biology, University of CambridgeCambridge, United Kingdom; ^3^ARC-Grain Crops InstitutePotchefstroom, South Africa; ^4^Functional and Comparative Genomics, Institute of Integrative Biology, University of LiverpoolLiverpool, United Kingdom; ^5^Grain Legume Genetics and Physiology Research Unit, Agricultural Research Service, US Department of AgricultureProsser, WA, United States; ^6^Sugarbeet and Bean Research Unit, Agricultural Research Service, US Department of AgricultureEast Lansing, MI, United States; ^7^Soybean Genomics and Improvement Laboratory, Agricultural Research Service, US Department of AgricultureBeltsville, MD, United States; ^8^Tropical Agriculture Research Station, Agricultural Research Service, US Department of AgricultureMayagüez, Puerto Rico; ^9^Department of Biology, Federal University of LavrasLavras, Brazil

**Keywords:** *Phaseolus vulgaris*, *Pseudomonas syringae* pv. *phaseolicola*, race-nonspecific and race-specific resistance, NLR, RNA-binding protein, plant immunity

## Abstract

*Pseudomonas syringae* pv. *phaseolicola* (*Psph*) Race 6 is a globally prevalent and broadly virulent bacterial pathogen with devastating impact causing halo blight of common bean (*Phaseolus vulgaris* L.). Common bean lines PI 150414 and CAL 143 are known sources of resistance against this pathogen. We constructed high-resolution linkage maps for three recombinant inbred populations to map resistance to *Psph* Race 6 derived from the two common bean lines. This was complemented with a genome-wide association study (GWAS) of Race 6 resistance in an Andean Diversity Panel of common bean. Race 6 resistance from PI 150414 maps to a single major-effect quantitative trait locus (QTL; HB4.2) on chromosome Pv04 and confers broad-spectrum resistance to eight other races of the pathogen. Resistance segregating in a Rojo × CAL 143 population maps to five chromosome arms and includes HB4.2. GWAS detected one QTL (HB5.1) on chromosome Pv05 for resistance to Race 6 with significant influence on seed yield. The same HB5.1 QTL, found in both Canadian Wonder × PI 150414 and Rojo × CAL 143 populations, was effective against Race 6 but lacks broad resistance. This study provides evidence for marker-assisted breeding for more durable halo blight control in common bean by combining alleles of race-nonspecific resistance (HB4.2 from PI 150414) and race-specific resistance (HB5.1 from cv. Rojo).

## Introduction

As an important contribution to the re-discovery of Mendelian genetics and its application in plant breeding, Biffen (1907, 1912) explained resistance to wheat yellow rust (*Puccinia striiformis*) by the inheritance of a single recessive gene and then deployed the resistance allele in new *Triticum* varieties. Most research on resistance to fungal phytopathogens has since focused attention on dominant disease resistance, which has been shown in numerous plant pathosystems to involve host receptor-like proteins that enable detection of pathogen effector proteins, consequently eliciting a host defense response (Dangl and Jones, 2001; Dodds and Rathjen, 2010). By contrast, recessive or incompletely dominant disease resistance may indicate that a host protein, which is targeted by the pathogen in a compatible interaction, is either absent or has been mutated to a dysfunctional form (Fraser, 1986, 1990). This has been supported by molecular evidence of mutations in non-receptor proteins conferring recessive resistance to viral, bacterial and fungal pathogens (Collmer et al., 2000; Kang et al., 2005; Iyer-Pascuzzi and McCouch, 2007; Orjuela et al., 2013; Wang et al., 2013). Importantly, combining alleles for receptor- and non-receptor-mediated resistance has been proposed as a strategy for providing durable disease control in crops (Leach et al., 2001; Iyer-Pascuzzi and McCouch, 2007).

Halo blight, caused by the bacterium *Pseudomonas syringae* pv. *phaseolicola* (*Psph*) (Burkholder, 1926; Young et al., 1978), is a disease of major economic significance that can plague common bean (*Phaseolus vulgaris* L.) production worldwide. This seed-transmitted disease favors cool and humid environments, occurring at higher latitudes in the northern and southern hemispheres and at higher altitudes in tropical and sub-tropical regions in Africa and South America. *Psph* causes yield losses of up to 45% (Asensio-S.-Manzanera et al., 2006; Singh and Schwartz, 2010; Félix-Gastélum et al., 2016). Genetic resistance provides the only viable control of this disease and is essential to the reliable production of disease-free seed, especially in bean-producing countries in East and Central Africa where smallholder farmers rely on seed saved from a previous harvest (Taylor et al., 1996a; Asensio-S.-Manzanera et al., 2006; Arnold, 2011; Miklas et al., 2014). Alternative hosts present in the tropics, coupled with the commonly short periods between consecutive crops, are additional factors that limit the feasibility of maintaining pathogen-free seed of susceptible varieties (Taylor et al., 1996a).

Seminal research established Phaseolus–Pseudomonas as an experimental pathosystem for investigating the molecular basis of plant–pathogen interactions (Taylor et al., 1996a,b). Nine races of *Psph* were identified from the interaction phenotypes between a large global collection of pathogen isolates and eight differential bean lines (Taylor et al., 1996a). The six most common *Psph* races were used to test a large diversity collection of *P. vulgaris* (1,048 accessions from the Americas and Africa) (Taylor et al., 1996b). From the combined data, they proposed a gene-for-gene model involving five major-effect, race-specific plant resistance (*R*) genes and corresponding pathogen avirulence (*avr*) genes (Taylor et al., 1996b). Subsequent genetic research based on linkage mapping identified the locations of major-effect *R* genes (*Pse-1* to *Pse-6*) on three chromosomes of *P. vulgaris* (Pv02, Pv04, and Pv10), which are effective against one or more *Psph* races (Miklas et al., 2009, 2011, 2014).

None of these race-specific genes provide resistance to the globally prevalent *Psph* Race 6, which continues to threaten bean production worldwide (Taylor et al., 1996a,b; Lamppa et al., 2002; Rico et al., 2003; Félix-Gastélum et al., 2016). However, Taylor et al. (1996b) identified bean accession PI 150414, a red dry bean landrace from El Salvador, as a source of quantitative, potentially race-nonspecific, resistance effective against Race 6. Patel and Walker (1965) had described this accession as a source for halo blight resistance, and Taylor et al. (1978) determined inheritance of the resistance as recessive or incompletely dominant expression of a single allele. This resistance has been used for development of new cultivars such as Wis HBR 72 (Hagedorn et al., 1974), Edmund (Conway et al., 1982) and Kranskop-HR 1. Important sources of resistance to Race 6 have been reported in popular African cultivars; the dry bean cultivar CAL 143 from East Africa possesses resistance to multiple diseases, including bean rust (*Uromyces appendiculatus*) (Murillo et al., 2006), angular leaf spot (*Phaeoisariopsis griseola*) (Chataika et al., 2010; Oblessuc et al., 2012) and halo blight (Chataika et al., 2011). This cultivar exhibited dominant resistance against endemic *Psph* field isolates (uncharacterized) present in Malawi, and preliminary evaluations of CAL 143 against the nine *Psph* races (at the ARC-Grain Crops Institute in Potchefstroom, South Africa) suggest that it possesses quantitative resistance to multiple races, including Race 6.

Minor-effect quantitative trait loci (QTL) for resistance to Race 6 have been described in previous reports. Trabanco et al. (2014) observed two minor-effect QTL (renamed herein as HB4.1 and HB6.1) in a RIL population (Xana × Cornell 49-242) that explained 11 and 12% of phenotypic variation. González et al. (2016) conducted a multi-environment study investigating the genetic basis of quantitative resistance to nine *Psph* races in primary and trifoliolate leaf, stem and pod tissues using an Andean RIL population (PMB0225 × PHA1037). They identified 11 minor-effect epistatic QTL without detectable additive effects (with each interaction explaining ≤ 8.52% of phenotypic variation), and one minor-effect QTL with both epistatic and individual additive effects (explaining 2.64 and 2.04% of phenotypic variation).

The objective of the current study was to fine-map major-effect resistance to Race 6 present in PI 150414, Edmund and CAL 143 using three recombinant inbred populations. The cultivar Edmund possesses Race 6 resistance derived from PI 150414 and the dominant, race-specific *R* gene *Pse-3* (Conway et al., 1982; Teverson, 1991; Taylor et al., 1996b). *Pse-3* confers qualitative resistance to Races 3 and 4, which are confined largely to East and Central Africa (Taylor et al., 1996a), and reportedly conditions quantitative resistance to other races (Taylor et al., 1996b; Fourie et al., 2004). Additionally, a subset of 384 accessions from an Andean Diversity Panel (ADP) (Cichy et al., 2015) was evaluated using a genome-wide association study (GWAS) for resistance to Race 6 in a field-based experiment in Potchefstroom, South Africa.

## Materials and methods

### Plant material and pathogen isolates

A SOA-BN × Edmund (SE) recombinant inbred population was developed at the University of Warwick Crop Centre, Wellesbourne, UK, and consisted of 80 F_6:7_ inbreds derived by single-seed descent. SOA-BN is a brown-seeded accession of northern European origin and is susceptible to all *Psph* races described by Teverson (1991). Edmund combines resistances to halo blight (*Pse-3* and quantitative resistance derived from PI 150414), *Bean common mosaic virus* (BCMV) and anthracnose (*Colletotrichum lindemuthianum*) (Conway et al., 1982).

Canadian Wonder × PI 150414 (CP) and Rojo × CAL 143 (RC) recombinant inbred populations were generated by USDA-ARS (Prosser, WA) and ARC-GCI (Potchefstroom), and consisted of 60 F_5:7_ and 147 F_5:7_ inbreds, respectively. The CP population represents a wide cross between the Andean and Middle American genepools. Canadian Wonder is the susceptible host differential cultivar (Taylor et al., 1996a) and PI 150414 is the well-described source of quantitative resistance used in breeding Edmund and other cultivars (Taylor et al., 1996b). The cultivar Rojo, a popular large-seeded red bean from Tanzania, possesses the *I* and *bc-1*^2^ genes for resistance to BCMV and uncharacterized resistance to halo blight, and is a component of an Andean Diversity Panel (ADP) of *P. vulgaris* (ADP-0096).

The ADP consists of 504 accessions that were assembled to represent the genetic diversity of cultivated beans within the Andean gene pool. A description of the individual accessions is available on the USDA-ARS, Feed the Future—Bean Research Team website^1^. A subset of 384 ADP accessions was chosen for this study.

All of the bacterial isolates used in this study were obtained from the Taylor collection of *Psph* maintained at Warwick Crop Centre. Bacterial isolates used for evaluating the SE population at Warwick included UK-725A (Race 1), Tanzania-1301A (Race 3) and UK-716B (Race 6). The CP and RC populations were evaluated under glasshouse conditions at Potchefstroom, South Africa against eight of the nine differential *Psph* races, including: UK-1281A (Race 1), USA-882 (Race 2), Tanzania-1301A (Race 3), Kenya-1375A (Race 5), Rwanda-1499B (Race 6), Rwanda-1449B (Race 7), Lesotho-2656A (Race 8) and Malawi-2709A (Race 9). The RC population and 384 ADP lines were tested under field conditions for reaction to Race 6 (Tanzania-1299A).

### Experimental design and phenotyping

**Figure 1:**
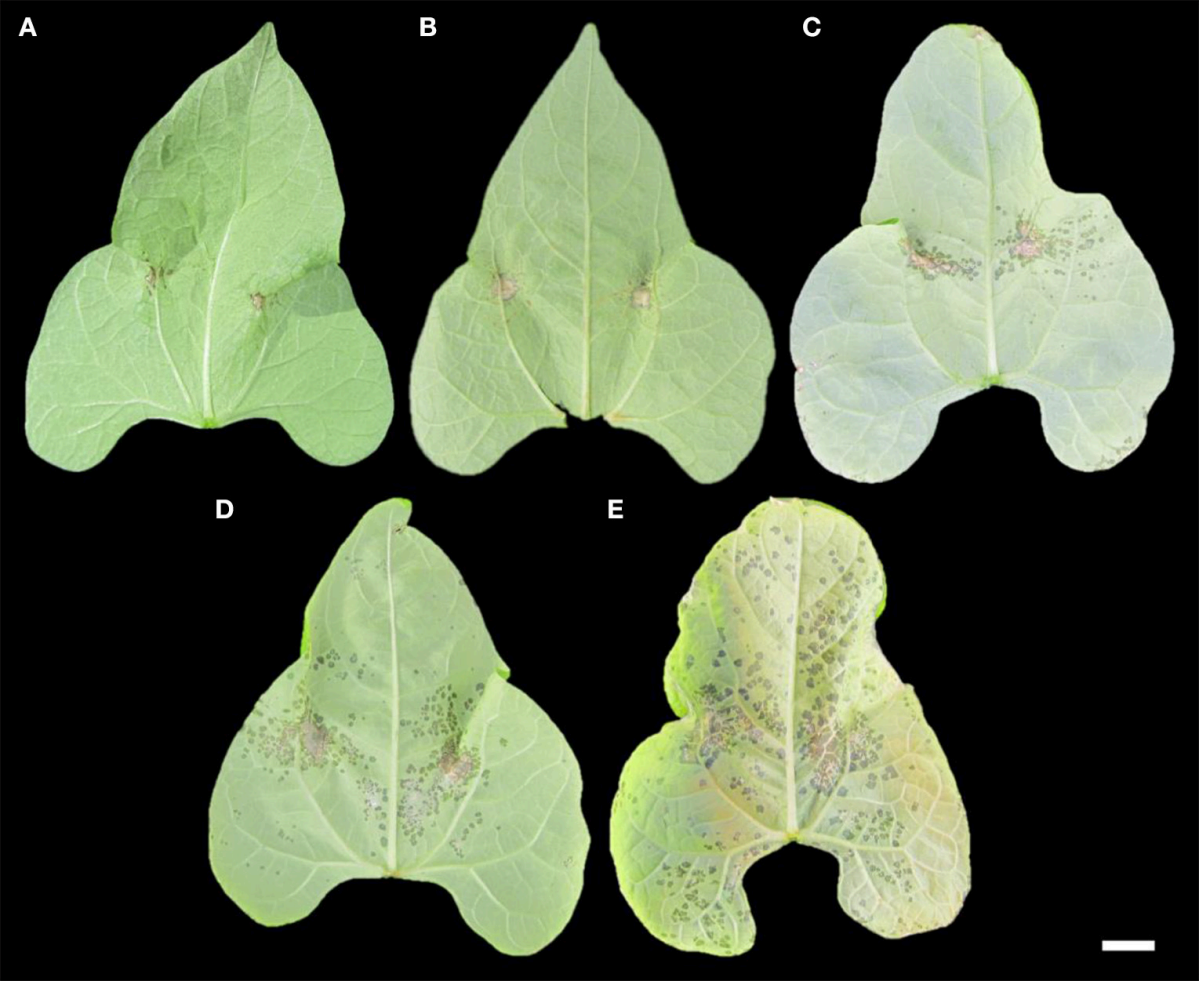
Interaction phenotypes exhibited by different *Phaseolus vulgaris* genotypes 10 days after inoculation with *Pseudomonas syringae* pv. *phaseolicola* Race 6 or Race 1, including **(A)** Red Mexican UI-3 (disease score = 1.0; highly resistant), **(B)** Edmund (disease score = 2.0; resistant), **(C)** SOA-BN × Edmund recombinant inbred line 113 (disease score = 3.0; slightly susceptible), **(D)** SOA-BN × Edmund recombinant inbred line 22 (disease score = 4.0; susceptible), and **(E)** SOA-BN (disease score = 5.0; fully susceptible). Scale bar: 1 cm.

For glasshouse experiments at Warwick Crop Centre, Wellesbourne, UK (52°12.54′N, 1°36.23′W), seeds of each bean line were sown in 7-cm plastic pots (one seed per pot) containing Levington M2 compost with vermiculite covering, arranged in a completely randomized design. Plants were grown under glasshouse conditions with a heating temperature of 18°C and a venting temperature of 20°C, and with supplementary lighting to provide a 16-h photoperiod. Bacterial inoculum was prepared by suspending a 48-h-old culture of Race 6 (strain 716B) grown at 24–25°C on King's B medium in sterile water and adjusting the suspension to 10^8^–10^9^ CFU ml^−1^ (as determined using a spectrophotometer). Ten-day-old seedlings were spray-inoculated as described by Taylor et al. (1996a). Additionally, one seedling of each parental line was mock-inoculated with sterile water. Inoculated and mock-inoculated seedlings were kept separately under conditions of high humidity by sealing them in 780 × 620 mm polypropylene autoclave bags for 48 h (10–15 plants per bag), before being returned to glasshouse conditions. One seedling of each parental line was included in each polypropylene bag as resistant and susceptible controls. Seedlings of Red Mexican UI-3 were included as highly resistant controls in Race 1 experiments. Seedlings of Guatemala 196-B, Tendergreen and ZAA 12 (A43) were included as severe hypersensitive resistant controls in Race 3 experiments. Phenotypic assessments were made according to the five-point scale of Innes et al. (1984) (Figure 1), with 1 being highly resistant (characterized by a necrotic reaction confined to the area of infiltrative inoculation) and 5 being highly susceptible (characterized by 1–3-mm-diameter water-soaked lesions distributed randomly and extensively over the primary leaf undersurface). Experiments were replicated in time to give up to five pseudo-replicates per RIL per bacterial isolate. The mean reaction of each RIL to each race was calculated from the infection scores assigned to replicate plants. Fixed- and random-effects variance components were estimated by restricted maximum likelihood (REML) analysis using the mixed-effects modeling package lme4 version 1.1–12, and tested using lmerTest v2.0-32 in R v3.3.0 (R Core Team, 2016).

For glasshouse experiments at ARC-GCI, Potchefstroom, South Africa (26°43′37.6″S, 27°04′40.3″E), 20 plants of each RIL, the parental lines, and the halo blight differential set (except Tepary 1072) (Taylor et al., 1996a) were grown in individual 8-cm-diameter plastic pots in sterile commercial potting soil (pH 6.9) and maintained in the glasshouse at 27/19°C day/night temperature with a 12-h photoperiod. Pots were randomized prior to inoculation. Seven- to ten-day-old seedlings with fully expanded primary leaves were inoculated, and plants were kept in a humidity chamber (19°C, 100% RH) for 48 h before being transferred to a glasshouse equipped with a humidifier (25/18°C day/night temperature, 70% RH). Plants were rated for disease severity 10 days after inoculation on the five-point scale of Innes et al. (1984). Least-squares means for disease severity score of each RIL were estimated using PROC GLM (SAS Institute, 2013). Reaction to *Bean common mosaic necrosis virus* (BCMNV) strain NL-3 D was used to map the *I* gene. This enabled the presence of *Pse-3* to be determined and its effect on the disease reaction to Races 1, 2, 5, 6, 7, 8, and 9 to be examined. Four plants of each line were inoculated with BCMNV strain NL-3 D as described by Larsen et al. (2005).

For field testing of the 384 ADP lines and the RC mapping population at the Potchefstroom ARC-GCI Station in South Africa, the soil was fertilized prior to planting using 3:2:1 of N:P:K at a rate of 250–300 kg ha^−1^. The ADP trial was hand-sown on January 24th, 2014 in single-row plots of 4 m length per line with a spacing of 0.75 m between rows, at a population density equivalent to 204,000 plants ha^−1^. Accessions were replicated three times in a randomized complete block design. All lines of the halo blight differential set (Taylor et al., 1996a) were included as checks, except for Tepary 1072. Herbicides Flumetsulam (20 g L^−1^) and S-metolachlor (640 g L^−1^) were applied at 1.7 L ha^−1^ directly after planting. Bacterial inoculum was prepared as described above. Plants were sprayed with inoculum using a Stihl SR430 mist-blower (~0.25 L per plot) at 21, 28, and 35 days after planting. Plots were irrigated (overhead) prior to inoculation, and repeated thereafter, at weekly intervals, to enhance disease development. The climatic conditions were conducive to halo blight development with a total rainfall of 386 mm (Jan.–Apr.) and temperatures of 14/27°C (min./max.). On March 24th and 25th, 2014 (mid-pod fill growth stage), disease severity was rated on a 1–9 scale as described by Van Schoonhoven and Pastor-Corrales (1987). Ratings were conducted on a plot basis as a collective score for all the plants within the row. At harvest maturity, the entire row was threshed to estimate seed yield (kg ha^−1^). Least-squares means for disease severity score and seed yield for the 384 ADP accessions for use in association mapping were generated using PROC GLM (SAS Institute, 2013). Trial design and management for evaluating the RC population was similar to the ADP trial, except lines were hand-sown on February 10th, 2015, there were six replications, and seed yield was not measured. Least-squares means for disease severity score of each RIL were estimated using PROC GLM (SAS Institute, 2013).

### Genotyping recombinant inbred populations

The SE population was characterized for genome-wide parental SNP variation using genotyping-by-sequencing (GBS) with ApeKI restriction enzyme-based complexity reduction (Elshire et al., 2011) to enable high-resolution map construction. Genomic DNA of each SE RIL and parental line was extracted from young trifoliolate leaves according to a CTAB protocol adapted from Afanador et al. (1993). Double-stranded DNA was quantified using a Qubit 2.0 Fluorometer (Invitrogen, USA). A GBS library was prepared according to previously published protocols using previously published barcode-adapters and PCR primers (Elshire et al., 2011), as optimized for common bean (Hart and Griffiths, 2015). The library was validated on an Agilent 2100 Bioanalyzer (Agilent Technologies, USA) and sequenced twice (at 48-plex) by 101-cycle single-end sequencing on two lanes of an Illumina HiSeq 2500 (Illumina, USA) at the Institute of Biotechnology Genomic Diversity Facility, Cornell University, USA. Sequencing reads were analyzed using the TASSEL v5.0 GBSv2 Discovery/Production Pipeline (Bradbury et al., 2007; Glaubitz et al., 2014). Resulting sequence tags were aligned to the *P. vulgaris* v1.0 reference genome (Schmutz et al., 2014) with the Burrows-Wheeler Aligner (Li and Durbin, 2009) and SNPs within aligned tags were called according to default pipeline parameters. Qualifying SNPs were those with minor allele frequency (MAF) ≥0.01 and for which ≥0.8 of samples were genotyped. Monomorphic and uninformative markers were removed, leaving 1,220 SNPs for linkage map construction (Map Data S1). The *I*/*Pse-3*-linked cleaved amplified polymorphic sequence (CAPS) marker targeting SNP ss715641188 developed by Bello et al. (2014) was included within this finalized set of markers.

The CP population was genotyped using the BARCBean6K_3 BeadChip containing 5,398 SNPs. After filtering, 1,284 SNPs were used for linkage map construction and QTL analysis as described below (Map Data S1).

The RC population was genotyped using GBS. Fresh leaf tissue of the first trifoliolate leaf from three seedlings from each RIL was bulked for genomic DNA extraction using the DNeasy 96 Plant Kit (Qiagen, Germany) according to the manufacturer's instructions. Double-stranded DNA was quantified with the QuantiFluor dsDNA Dye System and a Quantus Fluorometer (Promega, USA). The DNA was diluted to 5 ng μL^−1^ with nuclease-free water, and arrayed. A GBS library was constructed, validated and sequenced as described for the SE population, except using 149-plex and one lane of an Illumina HiSeq 2500 (Illumina, USA) at the Weill Cornell Medical College Genomics Resources Core Facility, Cornell University. The sequencing reads were processed with the GBS Discovery Pipeline (Glaubitz et al., 2014) as implemented in TASSEL v3.0.168 (Bradbury et al., 2007). GBS tags were aligned to the *P. vulgaris* v1.0 reference genome as described for the SE population, and the DiscoverySNPCaller plugin was used to call SNPs. As a result, 10,714 SNPs were discovered prior to filtering. This dataset was prepared for QTL analysis by removing all SNPs that were not called in ≥0.8 of the lines and by imposing a MAF of ≥0.1. The resulting dataset consisted of 1,004 SNPs which were used for linkage mapping (Map Data S1).

Five hundred ADP accessions from both the ADP#1 and ADP#2 panels^2^, including the 384 accessions that were phenotyped for resistance to halo blight, were also characterized for genome-wide SNP variation using GBS. Genomic DNA was extracted and quantified from the ADP accessions following the protocol outlined above for the RC population, except that ~50 mg of lyophilized leaf tissue from each accession was homogenized for DNA extraction. Two GBS libraries, one at 364-plex and one at 136-plex, were constructed and validated as described for the SE population. The 364-plex library was sequenced on five lanes and the 136-plex library was sequenced on two lanes of an Illumina HiSeq 2500 (Illumina, USA) at the Weill Cornell Medical College Genomics Resources Core Facility, Cornell University. Raw sequencing reads were deposited in the NCBI SRA under study accession SRP061551. The processing of sequencing reads for SNP identification used the same bioinformatics pipeline as described for the RC population. There were 17,750 SNPs for the 384 ADP accessions that met the criteria (MAF ≥0.05 and for which ≥0.8 of samples were genotyped) for association mapping. Missing data were imputed using Beagle v4.1 (Browning and Browning, 2016) with default parameters.

### Linkage map construction and QTL analysis

Linkage maps were constructed using MapDisto v2.0.b86 (Lorieux, 2012). Markers were assigned to linkage groups corresponding to the 11 common bean chromosomes (Pv01–Pv11; LOD_min_ = 3.0; *r*_max_ = 0.24). Markers were ordered according to linkage using the Seriation-II algorithm and the SARF (Sum of Adjacent Recombination Frequencies) locus-ordering criterion. The AutoRipple and AutoCheckInversions functions were applied repeatedly until each procedure could not find an improved order. Map distances were calculated using the Kosambi mapping function.

QTL analyses were conducted using R/qtl v1.39-5 (Broman et al., 2003) in R v3.3.0 (R Core Team, 2016). One-dimensional, single-QTL genome scans were performed using multiple imputation with a scan interval of 1 cM (imputations = 100; error probability = 0.001). Two-dimensional genome scans were performed using Haley-Knott regression with a scan interval of 1 cM (error probability = 0.001) to enable assessment of evidence for multi-QTL models involving additive or interacting loci. For each phenotype, genome-wide significance (α = 0.05) was determined using permutation tests (1,000 iterations). QTL support intervals were defined as the interval in which the LOD score is within 1.8 units of its maximum (Broman and Sen, 2009). The physical boundaries of each QTL were delineated by the positions of the closest markers flanking the 1.8-LOD support interval in the common bean reference genome. QTL models were fitted and refined using multiple imputation (scan interval = 1 cM; imputations = 100; error probability = 0.001) to derive the percentage of phenotypic variation explained by each locus. For association mapping using the 384 ADP accessions, population structure for GWAS was determined using principal component analysis (PCA) using the prcomp() function in R v3.2. An identity-by-state kinship matrix was created using the Efficient Mixed Model Association (EMMA) algorithm implemented in the Genome Association and Prediction Integrated Tool (GAPIT) R package (Lipka et al., 2012). Details of population structure within the ADP are provided in Cichy et al. (2015). The mixed linear model (MLM) implemented in GAPIT with a *P*-value of 0.05 was used to detect QTL conditioning field resistance to Race 6 and seed yield.

## Results

### Halo blight resistance to *Psph* race 6 in cv. Edmund (derived from PI 150414) maps to a single major-effect locus

**Figure 2:**
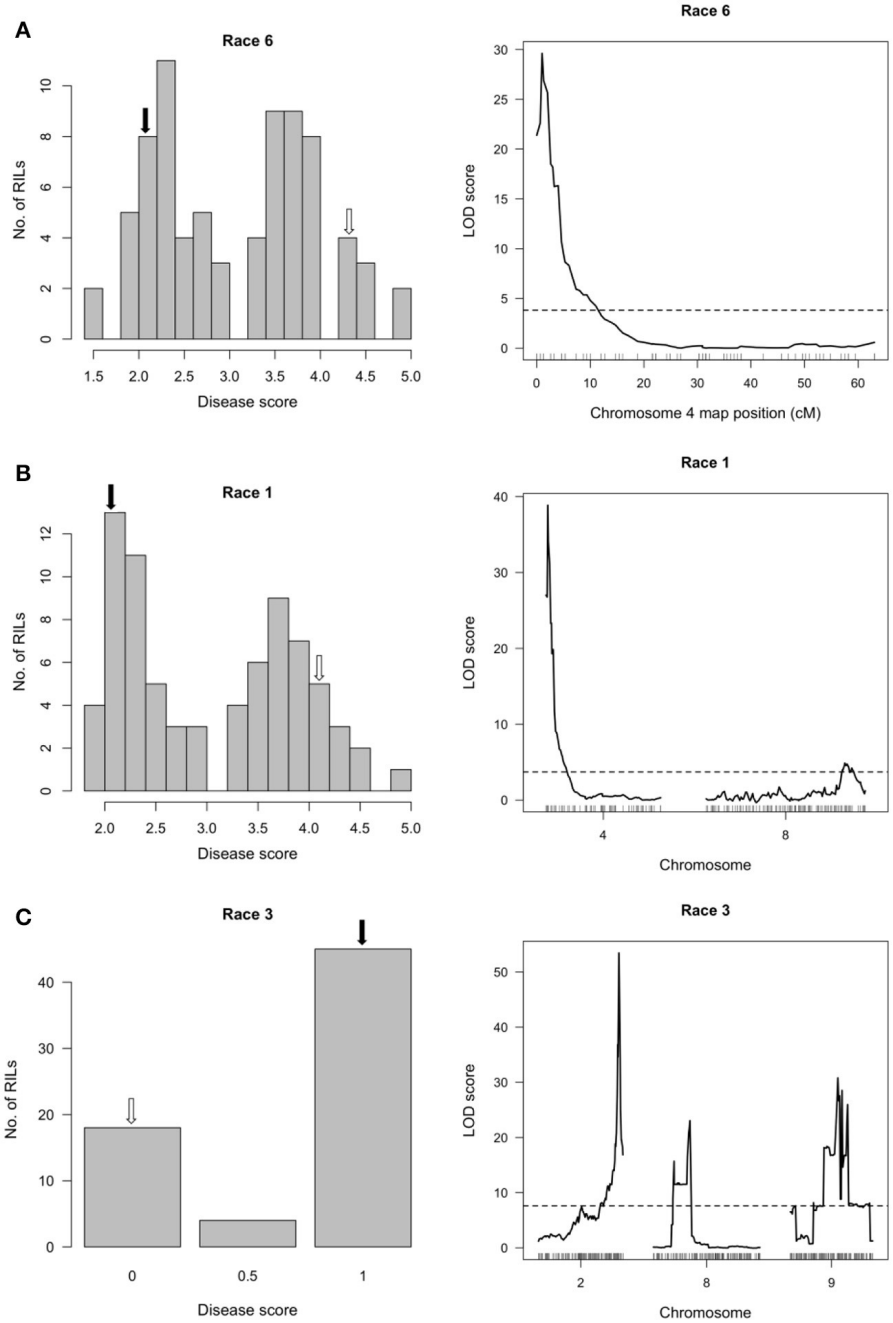
Resistance mapping in the *Phaseolus vulgaris* SOA-BN × Edmund (SE) recombinant inbred population following separate inoculations with **(A)** Race 6, **(B)** Race 1 or **(C)** Race 3 of *Pseudomonas syringae* pv. *phaseolicola* detects major-effect QTL on Pv04 and Pv02. **(Left graphs)** Distribution of interaction phenotypes exhibited by SE inbreds in glasshouse experiments. For reaction to Race 6 or to Race 1, the phenotype scale ranges from highly resistant (score 1.0) to fully susceptible (score 5.0). For reaction to Race 3, a score of 0 corresponds to full susceptibility and a score of 1 corresponds to a severe hypersensitive reaction, indicating presence of *Pse-3*. White arrows denote the mean disease score for SOA-BN and black arrows denote the mean disease score for Edmund. **(Right graphs)** LOD profiles obtained by one-dimensional and two-dimensional genome scans. QTL models were fitted and refined using the multiple imputation method. Dashed lines denote significance at the 0.05 probability level.

**Figure 3:**
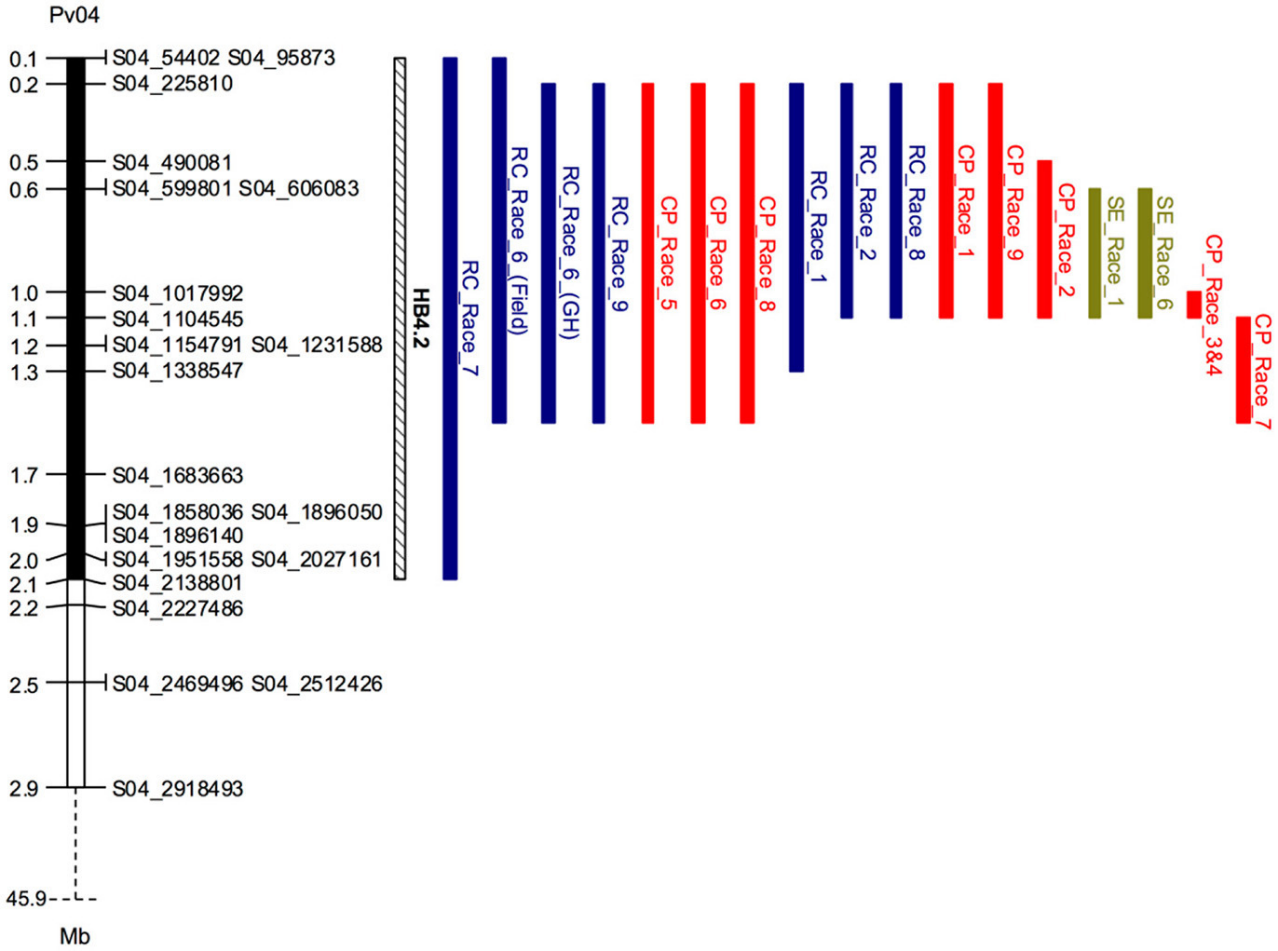
Co-localizing major-effect QTL (HB4.2) on Pv04 conferring resistance to multiple races of *Pseudomonas syringae* pv. *phaseolicola* (*Psph*) in SOA-BN × Edmund (SE; green), Canadian Wonder × PI 150414 (CP; red) and Rojo × CAL 143 (RC; blue) recombinant inbred populations. The physical boundaries of QTL 1.8-LOD support intervals are depicted as vertical bars, with population and *Psph* race indicated to the right of each interval. The locations of the closest SNP markers delimiting QTL intervals in the *P. vulgaris* v1.0 reference genome (Schmutz et al., 2014) are provided in megabase pairs (Mb). For clarity, the image depicts a magnified view of the top of Pv04.

In the SOA-BN × Edmund (SE) RIL population, halo blight resistance segregated in a bimodal distribution following separate inoculations with Race 6 and Race 1 in glasshouse experiments (Figures 2A,B, left graphs). Approximately 50% of the SE RILs had a mean disease score <3 (highly resistant to resistant/slightly susceptible) following inoculations with each race, and ~50% had a mean disease score >3 (slightly susceptible to fully susceptible). This supports the previous report by Taylor et al. (1978) indicating inheritance of a single major-effect halo blight resistance gene in PI 150414. One-dimensional, single-QTL genome scans mapped only one major-effect locus following inoculations with each race, which spanned a 500-kb interval on the short arm of Pv04 (delimited by SNP markers at 0.600 and 1.104 Mb) (Figures 2A,B, right graphs; Figure 3; Table S1; Tock, 2017). This QTL is distinct from the location of the previously mapped QTL HB4.1 (~44.0 Mb) that conditioned minor-effect resistance to Race 6 (Trabanco et al., 2014), and was thus designated HB4.2. A two-dimensional genome scan detected a second, minor-effect, additive QTL for reaction to Race 1 on Pv08 (HB8.1) at 57.955–58.844 Mb (Figure 2B, right graph; Figure S1; Table S1). Neither additive nor interacting minor-effect QTL were detected for reaction to Race 6.

The severe hypersensitive reaction to Race 3 (conferred by *Pse-3*) co-segregated with the *I* gene locus on Pv02 (48.398–48.534 Mb) (Figure 2C, right graph; Table S1), which is consistent with previous observations (Teverson, 1991; Miklas et al., 2011, 2014). Linked markers show a pattern of segregation distortion in favor of the parent possessing the resistance allele similar to that observed in the RC population (described below). Minor-effect QTL with additive and interactive relationships with *Pse-3* were also detected on Pv08 (HB8.2; 2.575–3.054 Mb) and Pv09 (HB9.1; 22.811–25.010 Mb) (Figure 2C, right graph; Table S1; Figure S2).

Based on the public reference genome of *P. vulgaris* (Schmutz et al., 2014), the 500-kb mapping interval on Pv04 spans a region that contains 38 genes (Table S2; Goodstein et al., 2012). This includes a cluster of 13 genes that are predicted to encode nucleotide-binding site–leucine-rich repeat (NLR) proteins with putative coiled-coil (CC) N-terminal domains. Four of these are predicted to be pseudogenes. Other candidates within this interval encode predicted defense-related proteins, including: an RNA recognition motif (RRM)-containing protein (Phvul.004G007600; Qi et al., 2010; Woloshen et al., 2011; Staiger et al., 2013); a mitogen-activated protein kinase kinase (MAPKK; Phvul.004G010400; Dóczi et al., 2007; Berr et al., 2010); a WD40 repeat-containing protein (Phvul.004G010500; Miller et al., 2016); an E3 ubiquitin protein ligase (UPL6; Phvul.004G009400; Duplan and Rivas, 2014); a protein with homology to an *Arabidopsis thaliana* Armadillo/β-catenin-like repeat protein (Phvul.004G009700; Sharma et al., 2014); a predicted cysteine-rich receptor-like kinase (CRK; Phvul.004G011000); and two plant self-incompatibility proteins (Phvul.004G010200 and Phvul.004G010300).

### Halo blight resistance from PI 150414 on Pv04 confers race-nonspecific resistance

**Figure 4:**
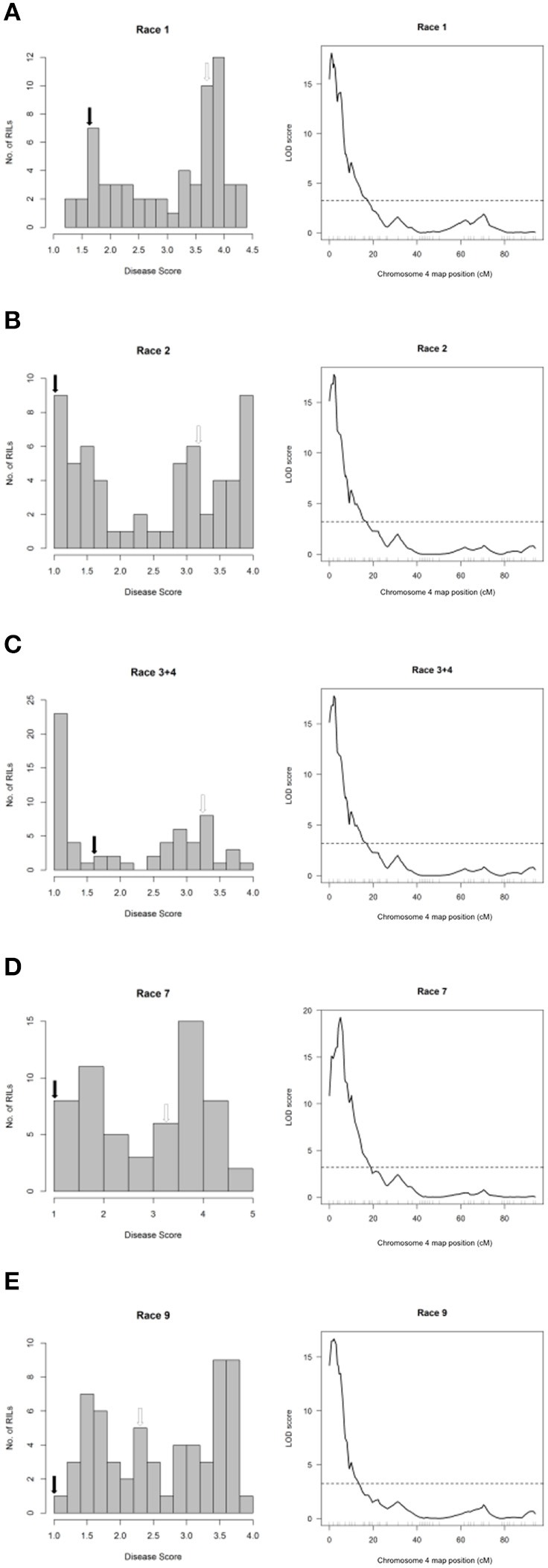
Resistance mapping in the *Phaseolus vulgaris* Canadian Wonder × PI 150414 (CP) recombinant inbred population following separate inoculations with **(A)** Race 1, **(B)** Race 2, **(C)** Race 3 + Race 4 (mixture), **(D)** Race 7, or **(E)** Race 9 of *Pseudomonas syringae* pv. *phaseolicola* detects co-localizing major-effect QTL on Pv04. **(Left graphs)** Distribution of interaction phenotypes exhibited by CP inbreds in glasshouse experiments. The phenotype scale ranges from highly resistant (score 1.0) to fully susceptible (score 5.0). White arrows denote the mean disease score for Canadian Wonder and black arrows denote the mean disease score for PI 150414. **(Right graphs)** LOD profiles obtained by one-dimensional and two-dimensional genome scans. QTL models were fitted and refined using the multiple imputation method. Dashed lines denote significance at the 0.05 probability level.

**Figure 5:**
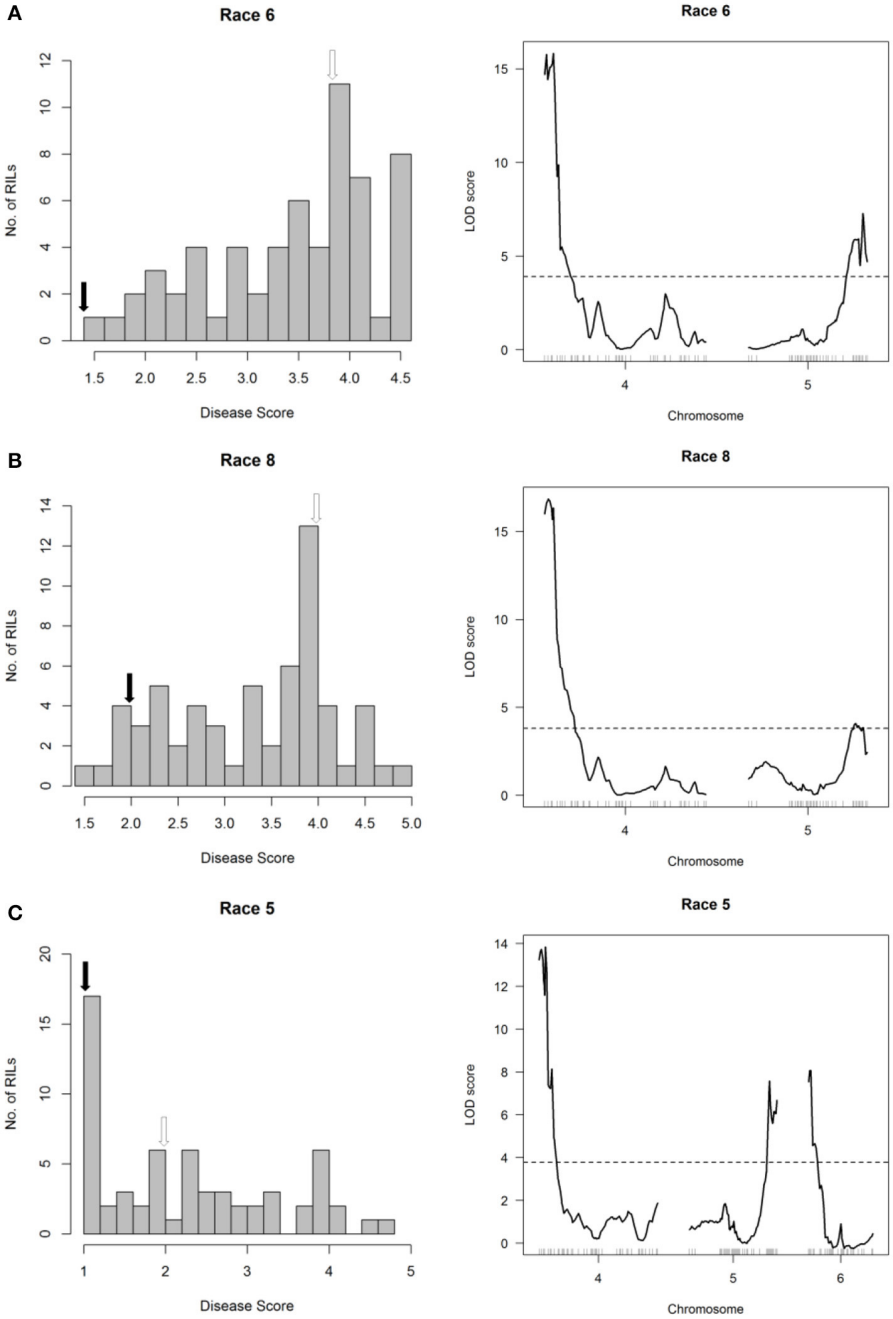
Resistance mapping in the *Phaseolus vulgaris* Canadian Wonder × PI 150414 (CP) recombinant inbred population following separate inoculations with **(A)** Race 6, **(B)** Race 8, or **(C)** Race 5 of *Pseudomonas syringae* pv. *phaseolicola* detects QTL on Pv04, Pv05, and Pv06. **(Left graphs)** Distribution of interaction phenotypes exhibited by CP inbreds in glasshouse experiments. The phenotype scale ranges from highly resistant (score 1.0) to fully susceptible (score 5.0). White arrows denote the mean disease score for Canadian Wonder and black arrows denote the mean disease score for PI 150414. **(Right graphs)** LOD profiles obtained by one-dimensional and two-dimensional genome scans. QTL models were fitted and refined using the multiple imputation method. Dashed lines denote significance at the 0.05 probability level.

The major-effect resistance from PI 150414 was further investigated with glasshouse evaluations of the Canadian Wonder × PI 150414 (CP) population of 60 RILs for reaction to representative isolates of the nine *Psph* races. Despite the small size of this population, one- and two-dimensional genome scans revealed that the major-effect QTL HB4.2 derived from PI 150414 (as detected in the SE RIL population) confers resistance to all eight of the races tested (Figure 3 and Figures 4, 5, right graphs; Table S3). Linkage analyses detected only one QTL following inoculations with six isolates representing Races 1, 2, 3, 4, 7, and 9 (Figure 4, right graphs), whereas an additional minor-effect QTL was detected on Pv05 (HB5.1) following inoculations with Races 5, 6, and 8 (Figure 5, right graphs). Significant interactions between HB4.2 and HB5.1 were detected for these three races (Figures S3, S4). HB5.1 was also detected by linkage mapping in the RC population (Table S4) and by association mapping in the ADP following inoculation with Race 6 (Table S7; described below). Evidently HB5.1 was not transferred from PI 150414 to Edmund because it was undetected in the SE RIL population.

An additional minor-effect QTL (HB6.1, previously mapped by Trabanco et al., 2014) was detected in the telomeric region of the short arm of Pv06 (0.175–15.787 Mb) following inoculation with Race 5, which also interacts with HB4.2 (Figure 5C, right graph; Table S3; Figure S4). This QTL overlaps and may correspond to the major-effect gene *Pse-4* for race-specific resistance to Race 5 described by Teverson (1991).

### Mapping of halo blight resistance from the RC population identifies race-specific alleles on five chromosome arms

**Figure 6:**
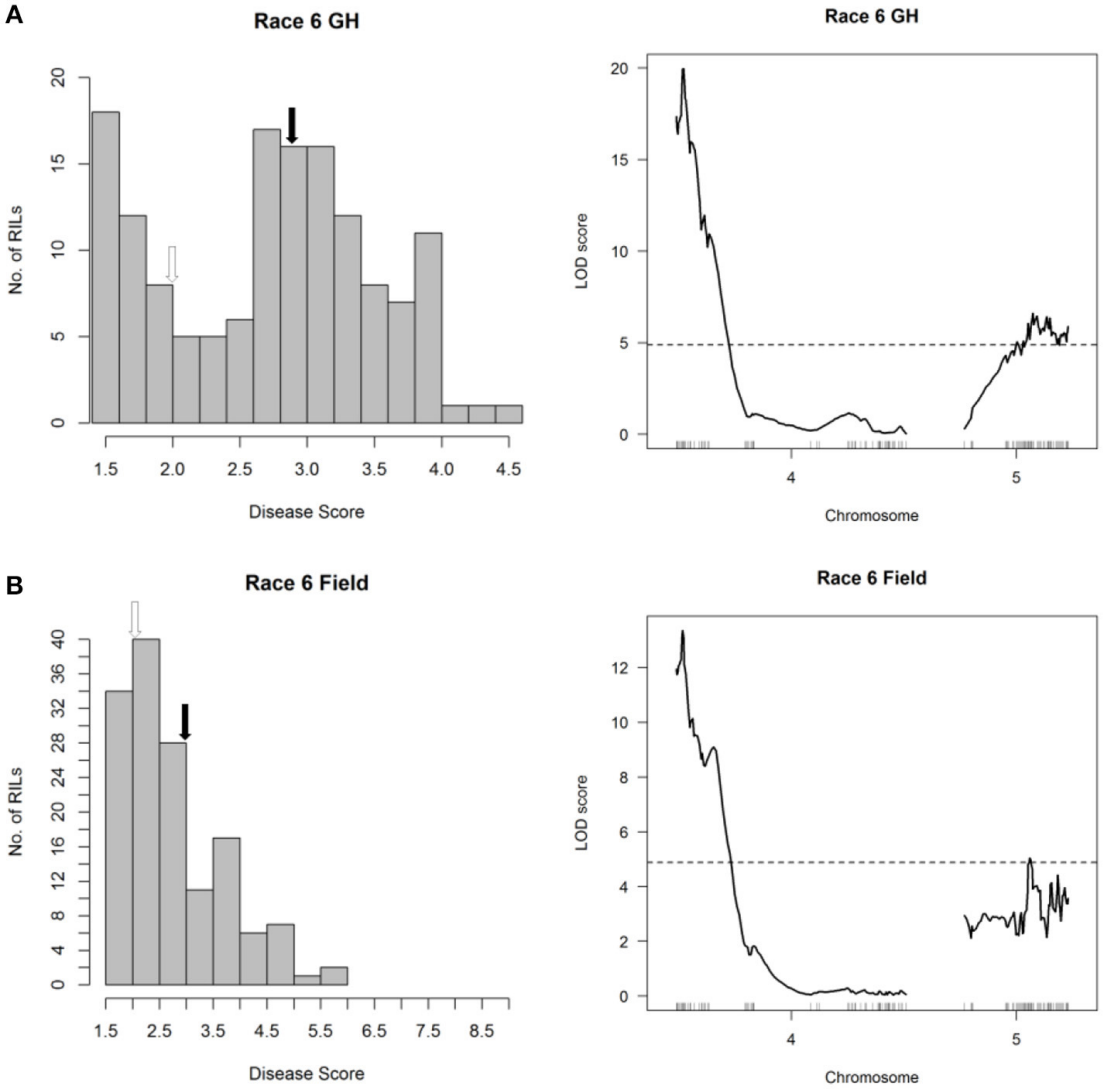
Resistance mapping in the *Phaseolus vulgaris* Rojo × CAL 143 (RC) recombinant inbred population following inoculation with Race 6 of *Pseudomonas syringae* pv. *phaseolicola* **(A)** in the glasshouse and **(B)** in the field detects co-localizing QTL on Pv04 and Pv05. **(Left graphs)** Distribution of interaction phenotypes exhibited by RC inbreds in glasshouse and field experiments. The phenotype scale ranges from highly resistant (score 1.0) to fully susceptible (score 5.0 for glasshouse experiments; score 9.0 for field experiments). White arrows denote the mean disease score for CAL 143 and black arrows denote the mean disease score for Rojo. **(Right graphs)** LOD profiles obtained by one-dimensional and two-dimensional genome scans. QTL models were fitted and refined using the multiple imputation method. Dashed lines denote significance at the 0.05 probability level.

Mapping of halo blight resistance in the RC population identified a major-effect QTL on Pv04 from CAL 143 and minor-effect QTL on Pv05 derived from Rojo following inoculations with Race 6 under both glasshouse and field conditions (Figure 3 and Figure 6, right graphs). HB4.2 and HB5.1 each contribute additively to Race 6 resistance (Figure S5). It is noteworthy that the seedling assay in the glasshouse was as useful as the field test for detecting HB4.2 and HB5.1 in the RC population, validating the utility of the rapid seedling assay for detecting field resistance to Race 6.

**Figure 7:**
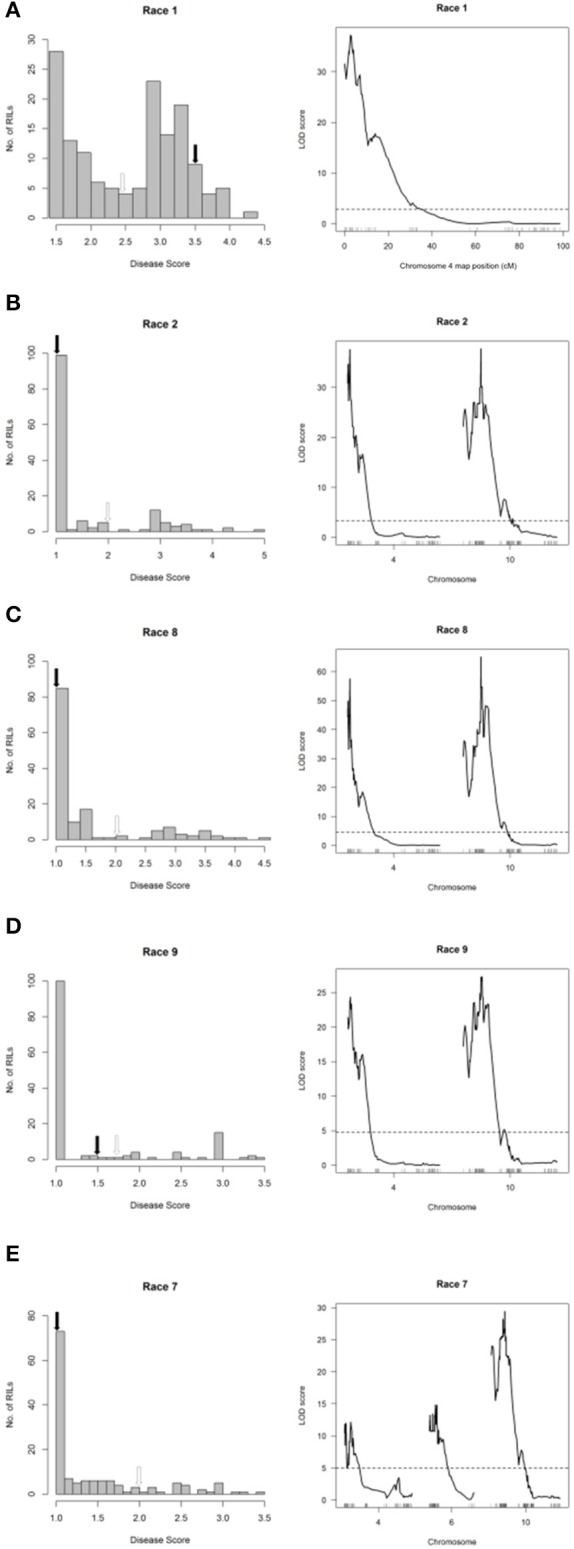
Resistance mapping in the *Phaseolus vulgaris* Rojo × CAL 143 (RC) recombinant inbred population following separate inoculations with **(A)** Race 1, **(B)** Race 2, **(C)** Race 8, **(D)** Race 9, or **(E)** Race 7 of *Pseudomonas syringae* pv. *phaseolicola* detects QTL on Pv04, Pv10, and Pv06. **(Left graphs)** Distribution of interaction phenotypes exhibited by RC inbreds in glasshouse experiments. The phenotype scale ranges from highly resistant (score 1.0) to fully susceptible (score 5.0). White arrows denote the mean disease score for CAL 143 and black arrows denote the mean disease score for Rojo. **(Right graphs)** LOD profiles obtained by one-dimensional and two-dimensional genome scans. QTL models were fitted and refined using the multiple imputation method. Dashed lines denote significance at the 0.05 probability level.

The major-effect QTL HB4.2 described above for the SE and CP populations was detected in the RC population following inoculations with Races 1, 2, 7, 8, and 9 (Figure 3 and Figure 7, right graphs). No minor-effect QTL were detected for resistance to Race 1 (Figure 7A, right graph). However, a significant locus was detected on Pv10 following inoculation with the other four races (Figures 7B–E, right graphs). Additionally, a third minor-effect QTL derived from Rojo was detected at the telomeric end of the short arm of Pv06 (0.346–14.964 Mb, also containing *V*, which contributes to flower and seed pigmentation) with Race 7 (Figure 7E, right graph). This QTL overlaps the HB6.2 QTL identified by Trabanco et al. (2014) which similarly confers resistance to Race 7, and thus will receive the same name.

**Figure 8:**
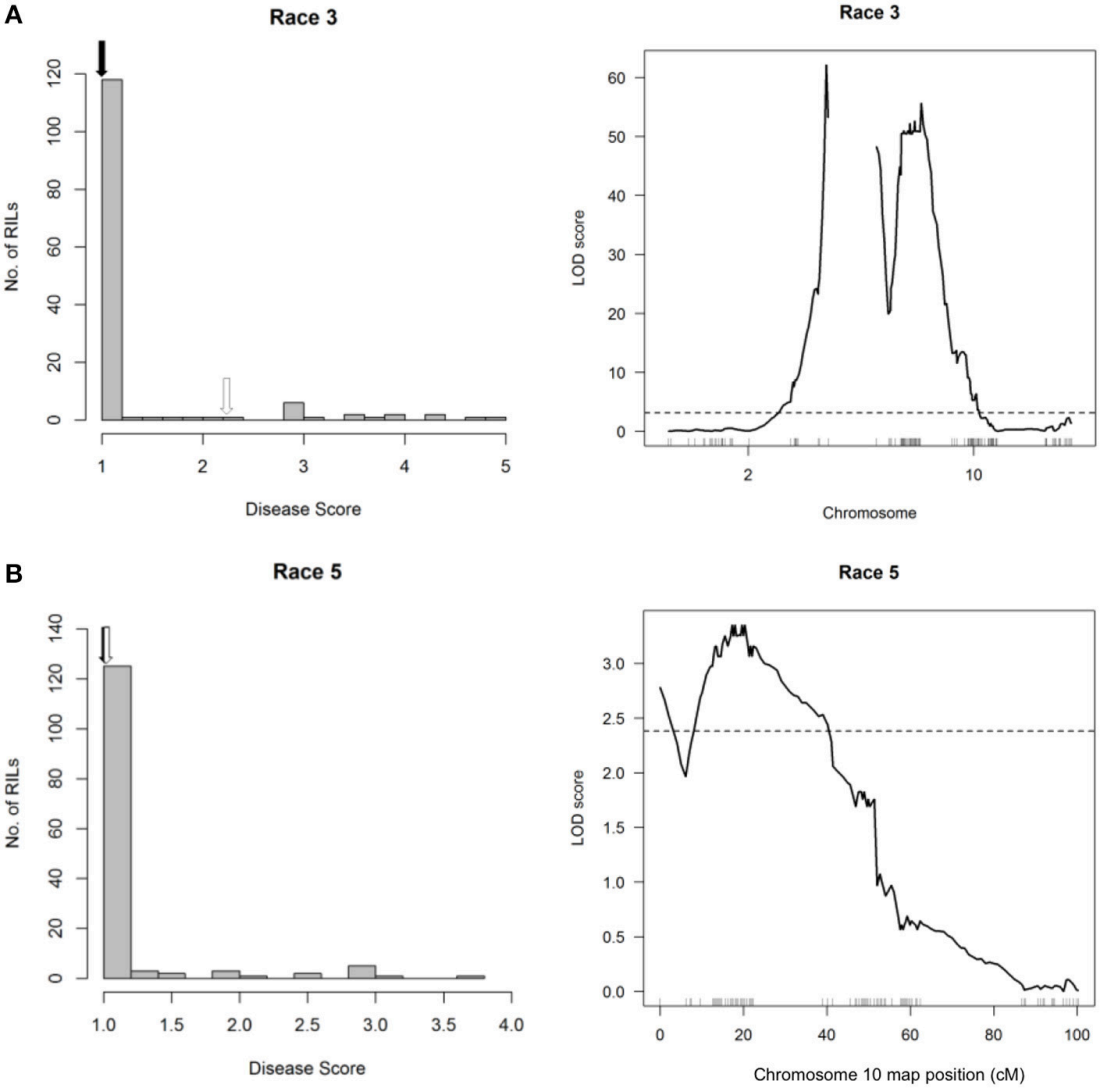
Resistance mapping in the *Phaseolus vulgaris* Rojo × CAL 143 (RC) recombinant inbred population following separate inoculations with **(A)** Race 3 or **(B)** Race 5 of *Pseudomonas syringae* pv. *phaseolicola* does not detect a QTL interval on Pv04. **(Left graphs)** Distribution of interaction phenotypes exhibited by RC inbreds in glasshouse experiments. The phenotype scale ranges from highly resistant (score 1.0) to fully susceptible (score 5.0). White arrows denote the mean disease score for CAL 143 and black arrows denote the mean disease score for Rojo. **(Right graphs)** LOD profiles obtained by one-dimensional and two-dimensional genome scans. QTL models were fitted and refined using the multiple imputation method. Dashed lines denote significance at the 0.05 probability level.

The broad-spectrum resistance conditioned by HB4.2 in CAL 143 is not conclusive because this QTL was not detected following inoculation of the RC population with Races 3 and 5 (Figure 8). Instead, these races mapped the same QTL on Pv10 (3.411–3.457 Mb) that was detected with Races 2, 7, 8, and 9, while an additional QTL was detected with Race 3 on Pv02 (47.368–48.296 Mb). These QTL correspond to race-specific loci *Pse-2* (conferring resistance to Races 2, 3, 4, 5, 7, 8, and 9) and *Pse-3* (conferring resistance to Races 3 and 4) that were previously mapped in other bean lines (Miklas et al., 2011). This may indicate that CAL 143 contains an alternative race-specific resistance allele in the HB4.2 mapping interval that is tightly linked but functionally distinct from the race-nonspecific allele in PI 150414. However, the expected resistance in CAL 143 to Races 3 and 5 appears to be unmapped in the RC population, and may be hypostatic in the presence of the major *Pse-2* and *Pse-3* genes inherited from the Rojo parent.

### Genome-wide association study using an andean diversity panel detects one major-effect locus on Pv05

A severe and uniform level of halo blight pressure (no detectable replication effects; coefficient of variation = 14%) was observed in the ADP field trial following multiple inoculations with Race 6. The mean disease score ranged from 1.7 (ADP-0121, Kranskop-HR 1) to 9 (ADP-310, ADP-349, ADP-0598, and ADP-623), with an overall mean of 5.9 (Table S5). Typical halo blight symptoms were observed on leaves. Many ADP accessions with severe symptoms also exhibited generalized systemic chlorosis, leaf yellowing, and malformation. Severe halo blight was also observed on pods, and included water-soaked round lesions with reddish spots and bacterial ooze. Yield decreased as the severity of symptoms increased (*r* = −0.50, *P* < 0.0001, *n* = 384) (Figure S6). Yield loss was estimated at 67% by comparing yield for the 23 ADP accessions with the highest levels of resistance (1,210 kg ha^−1^; score ≤ 3) to 45 of the most susceptible accessions (400 kg ha^−1^; score ≥8).

Race 6 inoculation in this field trial was confirmed by the susceptible reactions on the host differentials (Table S6), with the exception of Guatemala 196-B which had a score of 1. This was attributed to delayed maturity caused by poor vigor and lack of flowering due to photoperiod sensitivity (subsequent glasshouse tests with the same isolate used for field inoculation showed it to be susceptible). Interestingly, all of the differentials with at least one gene for resistance to halo blight were less susceptible than the universal susceptible check and differential cultivar Canadian Wonder, which lacks any genes for resistance. Differential lines with genes having broader effect (e.g., ZAA 12 with *Pse-2* for resistance to seven races, and UI-3 with *Pse-1* for resistance to four races) were less susceptible than the differentials ZAA 54 with *Pse-4* for Race 5 resistance, Tendergreen with *Pse-3* for resistance to Races 3 and 4, and ZAA 55 with both *Pse-3* and *Pse-4*. This suggests that presence of the major *R* genes for qualitative halo blight resistance may influence the quantitative response to Race 6 under field conditions.

**Figure 9:**
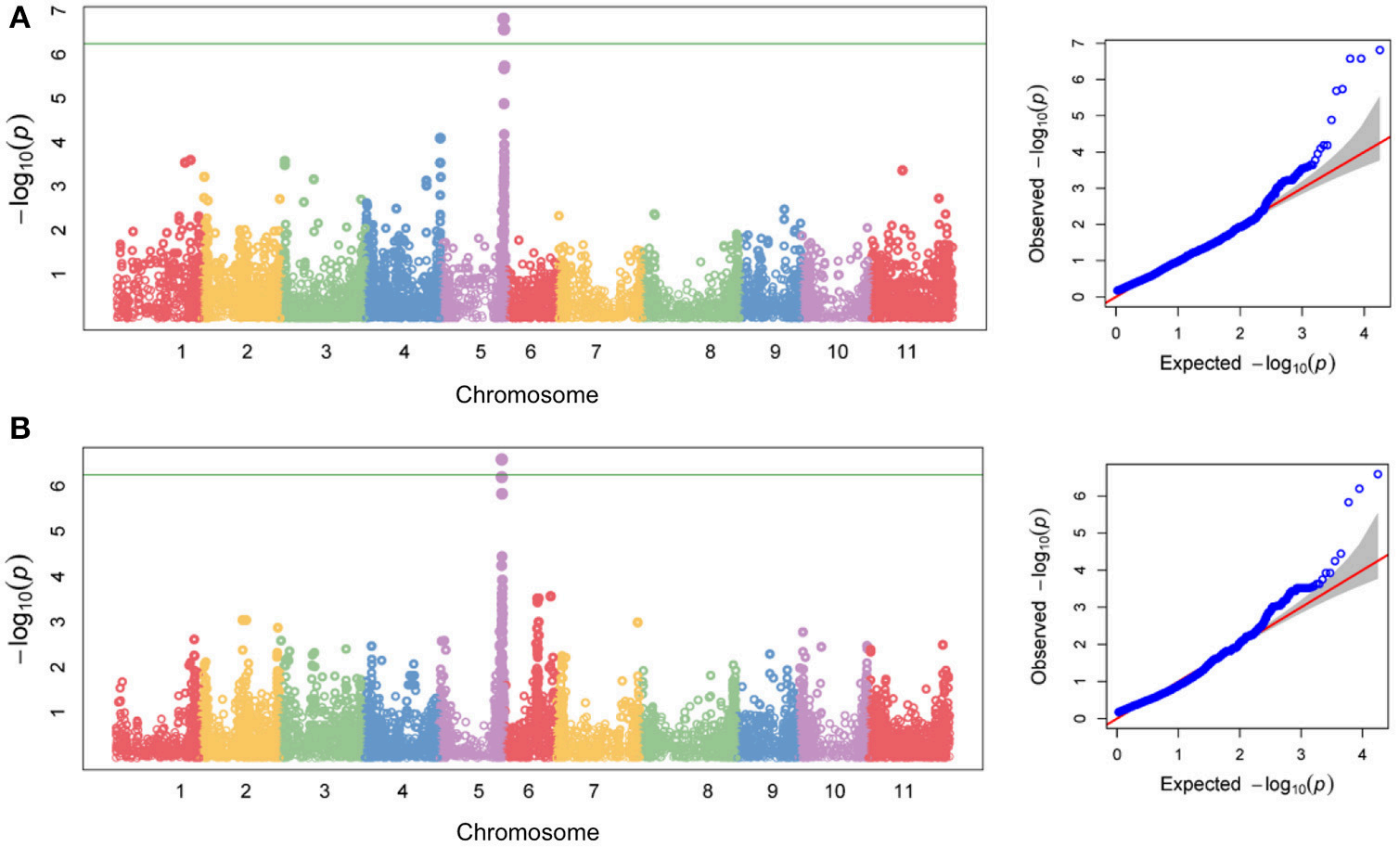
Genome-wide association mapping detects markers on Pv05 associated with field resistance to Race 6 of *Pseudomonas syringae* pv. *phaseolicola* in an Andean Diversity Panel of *Phaseolus vulgaris*. Chromosomal distribution of −log_10_(*P*) values for 17,759 SNP associations, and accompanying Q-Q plots for **(A)** disease score and **(B)** yield recorded using the Andean Diversity Panel under severe halo blight pressure following field inoculation with Race 6. Horizontal lines on Manhattan plots denote a significance threshold of *P* = 0.05.

**Figure 10:**
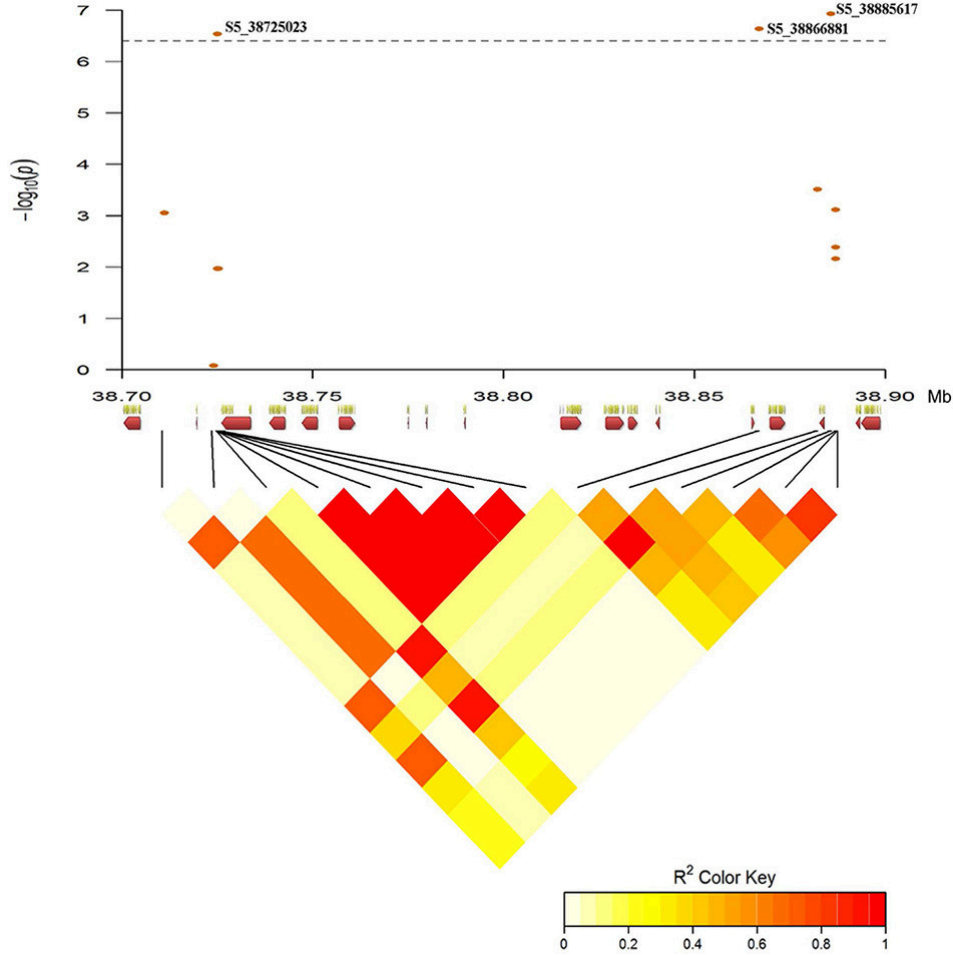
Pairwise linkage disequilibrium between significant SNP markers from GWAS defining HB5.1 QTL region on Pv05 was generated using the LDheatmap package in R v3.2. The x-axis depicts the 13 candidate genes (red arrows, Table S9) within the 162-kb region.

Association mapping identified a single QTL (named HB5.1) for Race 6 resistance on Pv05 (Figure 9A). The peak physical position (SNP S5_38725023) for this QTL is between 38.725 and 38.887 Mb (Table S7). The 162-kb interval of significance for this region is affected by high linkage disequilibrium (Figure 10). A locus significantly associated with yield was found at the same physical position (Figure 9B; Table S7), and confirms the importance of HB5.1 for reducing yield loss under severe halo blight pressure. On average, disease score was 23% higher and yield was 34% lower for accessions that lack the HB5.1 haplotype in the ADP (Table S8). The HB5.1 QTL explained 54% of phenotypic variation for disease score and 33% for yield.

Thirteen genes are located in the 162-kb interval of HB5.1 in the common bean reference genome (Schmutz et al., 2014). Four of these genes are not annotated and nine have predicted functional annotations related to leucine-rich repeat (LRR) protein kinases (Table S9; Goodstein et al., 2012).

## Discussion

This study provides evidence to enable marker-assisted breeding for more durable halo blight resistance in common bean, including protection against the most broadly virulent and globally distributed Race 6 of *Psph*, by combining alleles of race-nonspecific resistance (HB4.2 from PI 150414) and race-specific resistance (HB5.1 from cv. Rojo). Linkage mapping defined HB4.2 as the major-effect locus of 500 kb for race-nonspecific resistance at the telomeric end of the short arm of Pv04, and combined evidence from linkage and association mapping defined HB5.1 as a major-effect locus of 162 kb on Pv05 that confers resistance to at least three Races (5, 6, and 8). Resistance from a third major-effect locus (*Pse-2*) on Pv10 would provide additional protection against all *Psph* Races except Races 1 and 6.

### Candidate genes within the HB4.2 mapping interval

The 500-kb mapping interval for HB4.2 spans a cluster of 13 genes in the reference genome of *P. vulgaris* predicted to encode proteins with nucleotide-binding site and leucine-rich repeat domains, often referred to as NLR proteins (Table S2; Goodstein et al., 2012). NLRs are the most abundant class of receptor-like *R* proteins in plant genomes that confer resistance by enabling pathogen detection and defense signaling (Dangl and Jones, 2001; Dangl et al., 2013). NLRs generally confer dominant expression of race (pathogen genotype)-specific detection of corresponding avirulence (elicitor) proteins that are released into the host cytoplasm by obligate biotrophic or hemi-biotrophic pathogens (Glazebrook, 2005; Jones and Dangl, 2006; Mengiste, 2012). Single NLR genes that confer broad-spectrum (race-nonspecific) resistance have been described (Borhan et al., 2008, 2010). However, broad-spectrum resistance at HB4.2 could be explained by two or more NLR copies within the locus that provide combined resistance to all known *Psph* variants, including Race 6. Different resistance alleles at the *Pse-6* locus with alternative specificities for dominant, race-specific resistance have previously been mapped to HB4.2 (Miklas et al., 2014).

The race-nonspecific resistance at HB4.2 was originally described by Taylor et al. (1978) as being recessive or exhibiting weak dominance, depending on which susceptible parent was crossed with PI 150414. In the current study, symptoms of resistant inbreds from the SE population that were predicted to possess only resistance derived from PI 150414 did not exhibit hypersensitive cell death that is typically associated with NLR proteins, such as the phenotype shown in Figure 1A of a race-specific resistant differential host following inoculation with Race 1. Instead, resistance from PI 150414 was exhibited by necrosis at the sites of bacterial infiltration that was less pronounced and surrounded by a faint halo of water-soaked tissue following inoculation with Race 6 (Figure 1B).

Interestingly, numerous investigations of recessive disease resistance in other crop pathosystems have identified non-NLR proteins that confer dominant susceptibility to a pathogen (Collmer et al., 2000; Kang et al., 2005; Iyer-Pascuzzi and McCouch, 2007; Orjuela et al., 2013; Wang et al., 2013). In one case, a non-NLR gene and two tightly linked NLR genes in barley (*Hordeum vulgare* L.) were all determined to be essential components of resistance to a fungal rust (Wang et al., 2013).

A similar configuration of functional genes may explain halo blight resistance in PI 150414, because a non-NLR gene with polymorphic alleles (Phvul.004G007600) that encodes a predicted RNA-binding protein (RBP) is also located next to the NLR cluster within HB4.2 (Table S2). RBPs are a functional class of proteins that have been implicated in post-transcriptional regulation of plant immunity at various steps of RNA processing (Qi et al., 2010; Woloshen et al., 2011; Staiger et al., 2013). They contain characteristic conserved motifs that are predicted to facilitate binding of RNA targets required for execution of RNA-processing functions (Woloshen et al., 2011). Post-transcriptional gene regulation, partly enabled by RBPs, can promote rapid responses to biotic and abiotic stimuli. Further research is needed to confirm whether this gene and/or NLRs in the HB4.2 locus play a role in conferring halo blight resistance. However, polymorphism in the RBP gene will be extremely useful in future bean breeding as a co-segregating marker for the race-nonspecific resistance.

### Candidate race-specific genes within the HB5.1 mapping interval

The 162-kb mapping interval for HB5.1 includes a cluster of at least nine genes predicted to encode transmembrane receptor-like genes with a leucine-rich repeat domain and a cytoplasmic kinase, often referred to as RLKs (Table S9; Goodstein et al., 2012). RLKs are a large class of proteins that provide numerous roles for cell-to-cell communication in plant development (Shiu and Bleecker, 2001). As transmembrane proteins, they can also enable detection of extracellular microbial elicitors called pathogen-associated molecular patterns (PAMPs) such as bacterial flagellin or fungal chitin (Sanabria et al., 2008; Dodds and Rathjen, 2010). PAMPs are generally highly conserved among diverse microbial species (Boyd et al., 2013). Two RLK examples have been described which confer non-host resistance to *Psph* in *A. thaliana*, including *FLS2* which enables detection of flagellin (Forsyth et al., 2010; Ahmad et al., 2011). In *P. vulgaris*, RLKs on Pv01 and Pv08 have been identified as candidates for race-specific resistance to anthracnose caused by *Colletotrichum lindemuthianum* (Burt et al., 2015; Zuiderveen et al., 2016).

### Association mapping detects a single locus for *Psph* race 6 resistance

Genome-wide association mapping is a powerful approach for identifying multiple polymorphisms that underlie natural variation within a species. If there is sufficient diversity within a sample population (e.g., a species diversity panel), then this approach can provide higher resolution mapping, greater allelic diversity, and improved efficiency and accuracy in estimating marker effects for quantitative traits than bi-parental linkage mapping (Flint-Garcia et al., 2003; Myles et al., 2009). For instance, this approach has been used to identify markers associated with complex inheritance of resistance to fungal diseases of *P. vulgaris* including anthracnose and angular leaf spot (Perseguini et al., 2016; Zuiderveen et al., 2016).

In the current study, only one locus (HB5.1) was significantly associated with resistance to Race 6 in the ADP. This is not surprising given the low occurrence of halo blight resistance in an extensive testing of Andean and African germplasm by Taylor et al. (1996b). Other loci identified using bi-parental linkage mapping populations, such as HB4.2, are presumably too rare for significant detection by association in the ADP. Ghising et al. (2016) previously identified regions on Pv04 (1.2 Mb) and Pv05 (39.4 Mb) associated with resistance to Race 6 using a USDA-ARS National Plant Germplasm System (NPGS) core collection of 383 Mesoamerican and Andean bean accessions. Ten accessions were highly resistant. However, the associated markers identified on Pv04 and Pv05 accounted for low percentages of phenotypic variation for Race 6 resistance (each ≤ 8%).

### Marker-assisted breeding strategy for halo blight resistance

Taylor et al. (1996b) proposed that a breeding strategy for durable halo blight resistance should combine race-nonspecific (derived from PI 150414) and race-specific resistance that matches the predominant *Psph* races in a particular region. For the latter, the predicted *Pse-3* (syn. *R3*) gene was lacking in East and Central African bean varieties that were used in the study, and was therefore recommended for breeding efforts to provide additional control of the common Race 4 for smallholder farmers in the region. Although the broadly virulent Race 6 can be used to select for race-nonspecific resistance, given the recessive nature of this resistance, co-segregating molecular markers such as the RBP polymorphisms that delimit HB4.2 are still necessary to improve breeding efficiency for combining this resistance with one or more alleles of race-specific resistance. Given that resistances to other pathogens have been mapped to this region of Pv04 (Miklas et al., 2006, and references therein), efforts to introgress HB4.2 will need to avoid displacement of *R* genes that are in repulsion phase linkage with this new breeding target.

This study demonstrates how genotyping-by-sequencing can provide an efficient platform for fine mapping of halo blight resistance in *P. vulgaris* even with relatively small inbred populations. Importantly, using this method for combined linkage and association mapping delineated race-nonspecific and race-specific resistance loci distributed over five chromosome arms, as summarized in Table 1. The detection of major-effect loci HB4.2 and HB5.1 on Pv04 and Pv05 provide molecular markers to assist breeding for resistance to *Psph* Race 6, and combining this resistance with other alleles conferring race-specific resistance. Specifically, SNPs spanning HB4.2 and HB5.1 regions from PI 150414 provide an exceptional resource to facilitate marker-assisted introgression of resistance into common beans of Middle American origin, whereas SNPs spanning HB4.2 from CAL 143 and HB5.1 from Rojo will be useful for introgression of resistance to Race 6 into lines of Andean origin. Another important resource from this study will be RC RILs that combine both HB4.2 and HB5.1 QTL with both qualitative *R* genes (*Pse-2* and *Pse-3*) in different market classes and with the best agronomic traits (currently under evaluation). These will be officially released as germplasm lines to promote global availability of pyramided resistance to halo blight.

**Table 1:** Interaction phenotypes exhibited by halo blight-resistant *Phaseolus vulgaris* lines of the SOA-BN × Edmund (SE), Canadian Wonder × PI 150414 (CP) and Rojo × CAL 143 (RC) recombinant inbred populations and the Andean Diversity Panel (ADP) following inoculation with different races of *Pseudomonas syringae* pv. *phaseolicola*, with QTL and predicted resistance (*R*) genes indicated.

**Population**	**QTL/*R* gene**	**Chrom**.	**Race**								
			**1**	**2**	**3**	**4**	**5**	**6**	**7**	**8**	**9**
CP, SE, RC	HB4.2	Pv04	−(q)	−(q)	−(q)	−(q)	−(q)	−(q)	−(q)	−(q)	−(q)
CP, RC, ADP	HB5.1	Pv05	+	+	+	+	−(q)	−(q)	+	−(q)	+
CP	HB6.1/*Pse-4*?	Pv06	+	+	+	+	−(q)	+	+	+	+
RC	HB6.2	Pv06	+	+	+	NT	+	+	−(q)	+	+
SE	HB8.1	Pv08	−(q)	NT	+	NT	NT	+	NT	NT	NT
SE	HB8.2	Pv08	+	NT	−(q)	NT	NT	+	NT	NT	NT
SE	HB9.1	Pv09	+	NT	−(q)	NT	NT	+	NT	NT	NT
RC	*Pse-2*	Pv10	+	−	−	NT	−	+	−	−	−
SE, RC	*Pse-3*	Pv02	+	+	−HR	NT	+	+	+	+	+

*Chrom., chromosome; +, apparent susceptible (compatible) reaction; −, apparent resistant (incompatible) reaction; −(q), apparent quantitative resistance; −HR, apparent resistant reaction with severe hypersensitive cell death; NT, not tested; HB6.1 and HB6.2 were first identified in the Xana × Cornell 49-242 RIL population (Trabanco et al., 2014)*.

## Author contributions

AT: population development, data generation, organization, analysis and interpretation, and co-writing and editing of the manuscript. DF: population and data generation, and glasshouse and field inoculations. PW: statistical genetics training and technical support for AT, results interpretation and co-editing of drafts. EH: overall supervision of UK-funded research including study conception, pathology and genetic interpretation, and co-writing and editing of the manuscript. AS: data analysis and interpretation. JV: pathology training and technical support for AT. KC: technical support. MP: phenotyping. QS: SNP assays. TP: ADP population development and phenotyping. JH: library construction and bioinformatics for genotyping-by-sequencing. RV: preliminary QTL analyses. GB: co-supervision of AT's Ph.D. and genomics support. PM: study conception of US-funded research, population and data generation and interpretation, and co-writing and editing of the manuscript.

### Conflict of interest statement

The authors declare that the research was conducted in the absence of any commercial or financial relationships that could be construed as a potential conflict of interest.

## Footnotes

^1^http://arsftfbean.uprm.edu/bean/?p=472

^2^http://arsftfbean.uprm.edu/bean/?p=472
