# Supplementary material for: Genome-Wide Linkage and Association Mapping of Halo Blight Resistance in Common Bean to Race 6 of the Globally Important Bacterial Pathogen
Source: Front Plant Sci. 2017 Jul 7;8:1170. doi: 10.3389/fpls.2017.01170 (PMC5500643; doi:10.3389/fpls.2017.01170)
Supplement: Supplementary file 1 [file DataSheet1.docx]

Supplementary Material

Genome-wide linkage and association mapping of halo blight resistance in common bean to Race 6 of the globally important bacterial pathogen

**Andrew J. Tock^1,2^, Deidré Fourie^3^, Peter G. Walley^4^, Eric B. Holub^1^, Alvaro Soler^5^, Karen A. Cichy^6^, Marcial A. Pastor-Corrales^7^, Qijian Song^7^, Timothy G. Porch^8^, John P. Hart^8^, Renato C. C. Vasconcellos^9^, Joana G. Vicente^1^, Guy C. Barker^1^ and Phillip N. Miklas^5,^***

^1^ School of Life Sciences, Faculty of Science, University of Warwick, Wellesbourne, UK

^2^ Department of Plant Sciences, Faculty of Biology, University of Cambridge, Cambridge, UK

^3^ ARC-Grain Crops Institute, Potchefstroom, South Africa

^4^ Functional and Comparative Genomics, Institute of Integrative Biology, University of Liverpool, Liverpool, UK

^5^ USDA-ARS, Grain Legume Genetics and Physiology Research Unit, Prosser, WA, USA

^6^ USDA-ARS, Sugarbeet and Bean Research Unit, East Lansing, MI, USA

^7^ USDA-ARS, Soybean Genomics and Improvement Laboratory, Beltsville, MD, USA

^8^ USDA-ARS, Tropical Agriculture Research Station, Mayagüez, Puerto Rico

^9^ Department of Biology, Federal University of Lavras, Lavras, MG, Brazil

*** Correspondence:**Phillip N. Miklas
[phil.miklas@ars.usda.gov](mailto:phil.miklas@ars.usda.gov); +1-509-786-9258

# Supplementary Figures


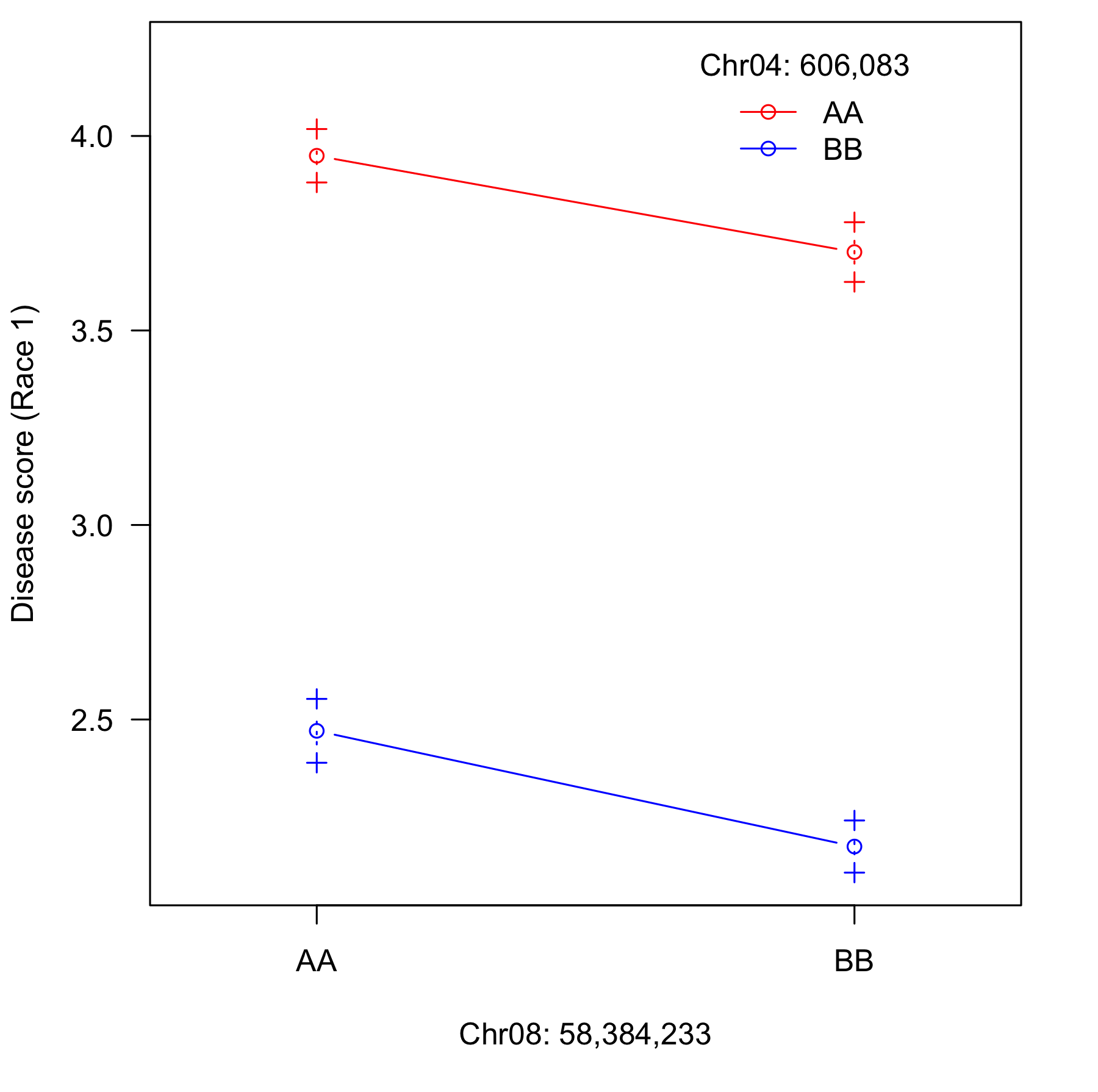


**Figure S1.**  Additive relationship between the major-effect QTL on Pv04 and the minor-effect QTL on Pv08 conditioning resistance to *Pseudomonas syringae* pv. *phaseolicola* Race 1 in the *Phaseolus vulgaris* SOA-BN × Edmund recombinant inbred population. AA: SOA-BN (susceptible) parental genotype; BB: Edmund (resistant) parental genotype.

**A**


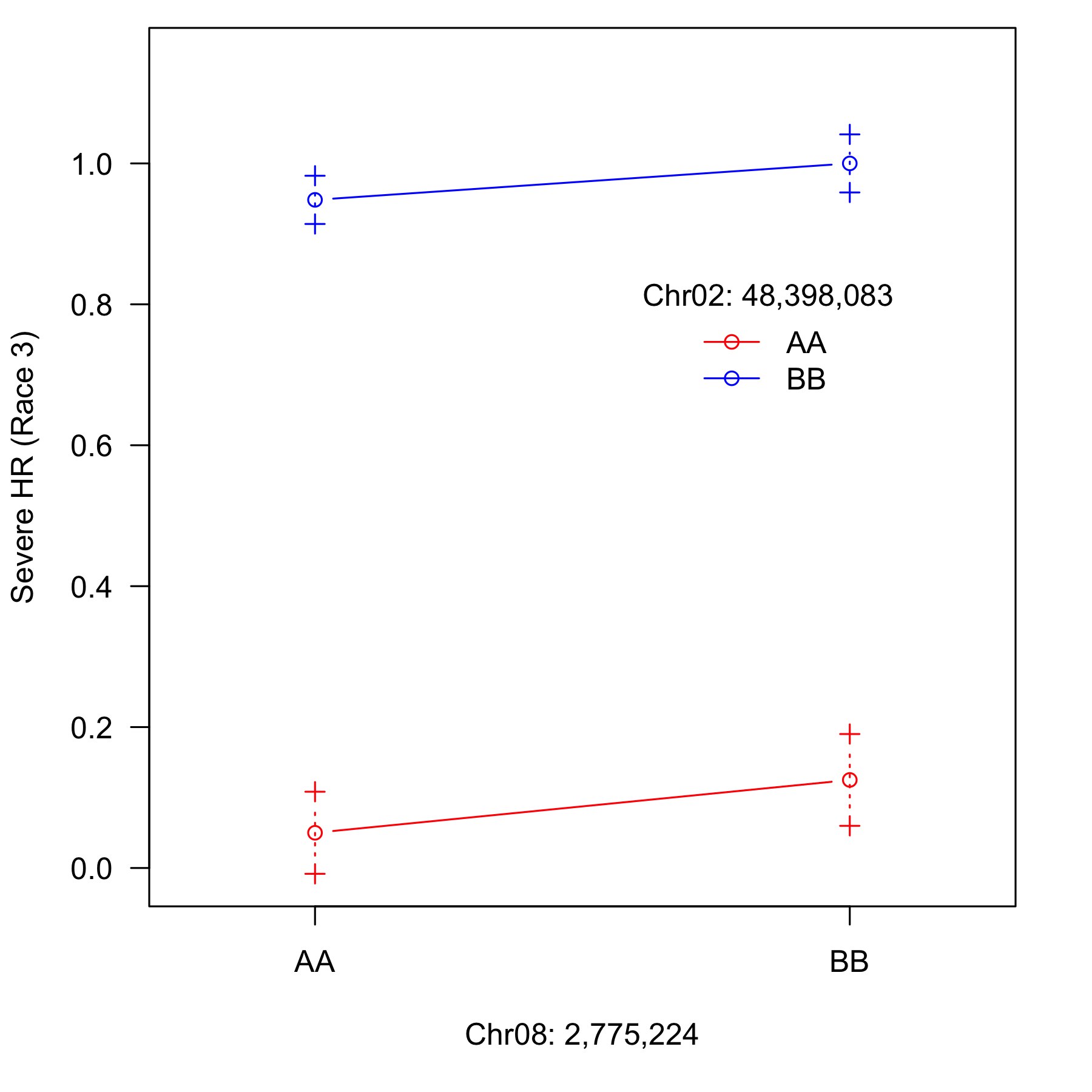


**B**


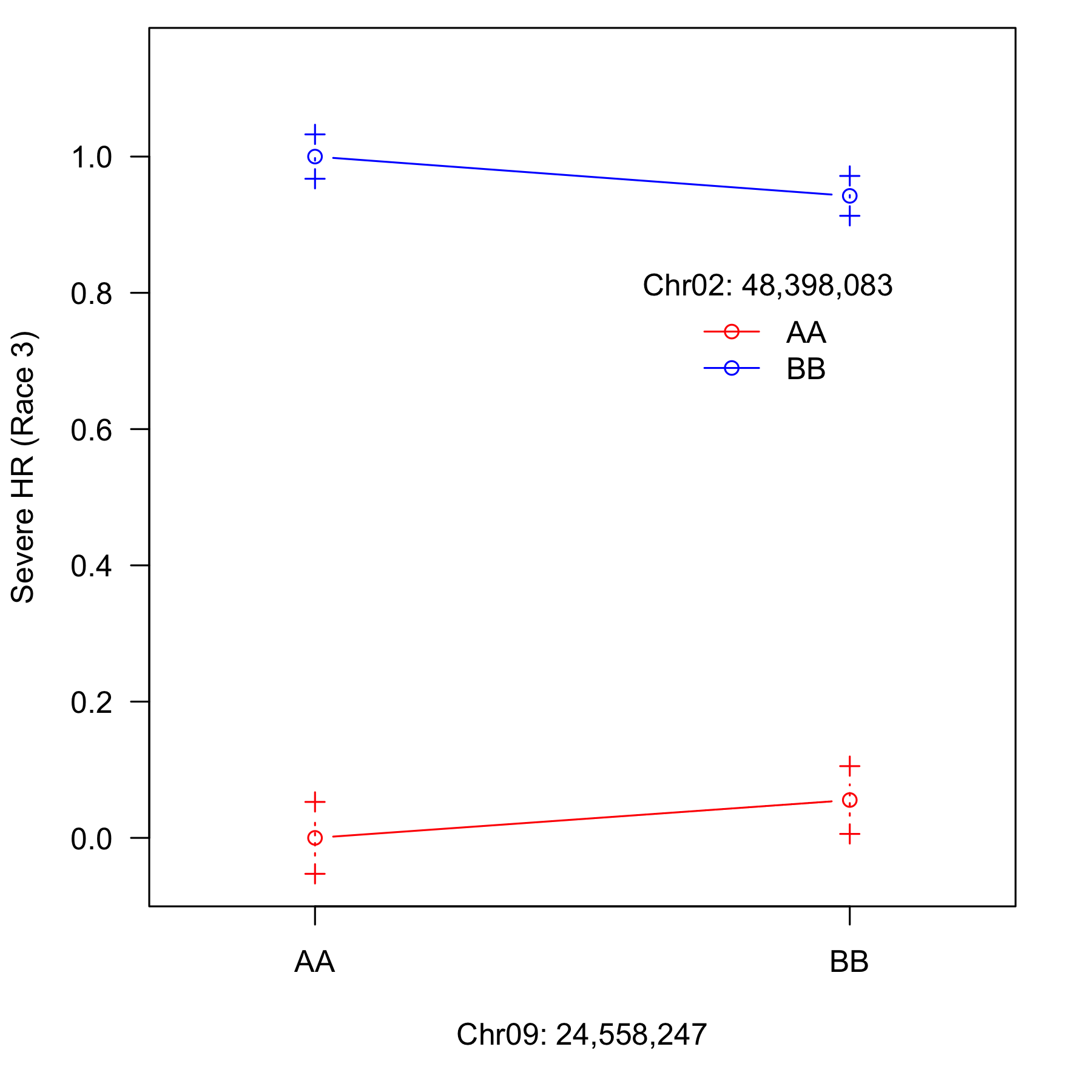


**Figure S2.** Additive and interactive relationships between the major-effect QTL on Pv02 and the minor-effect QTL on **(A)** Pv08 and **(B)** Pv09 conditioning resistance to *Pseudomonas syringae* pv. *phaseolicola* Race 3 in the *Phaseolus vulgaris* SOA-BN × Edmund recombinant inbred population. AA: SOA-BN (susceptible) parental genotype; BB: Edmund (resistant) parental genotype. HR: hypersensitive response.

**A**

**
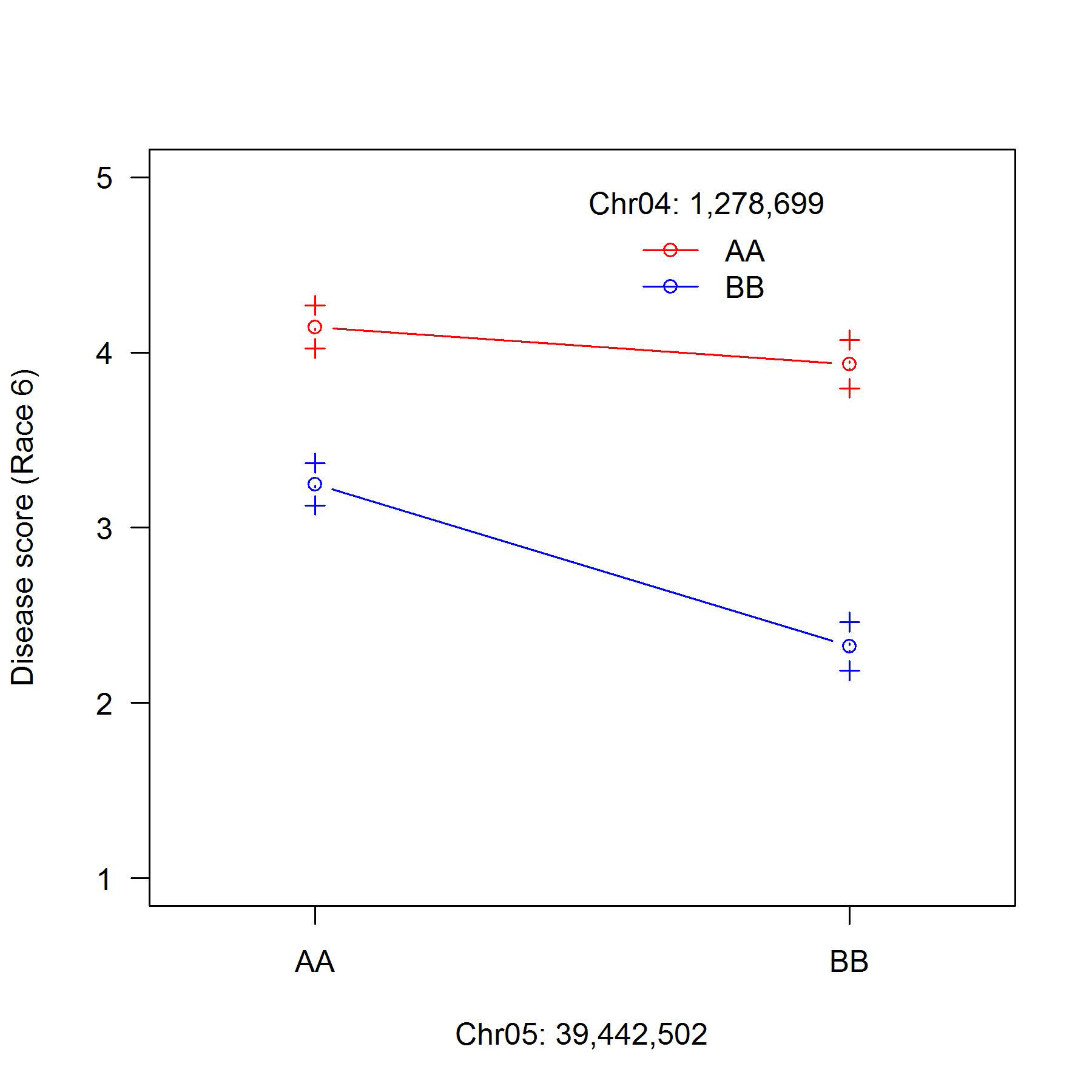
**

**B**


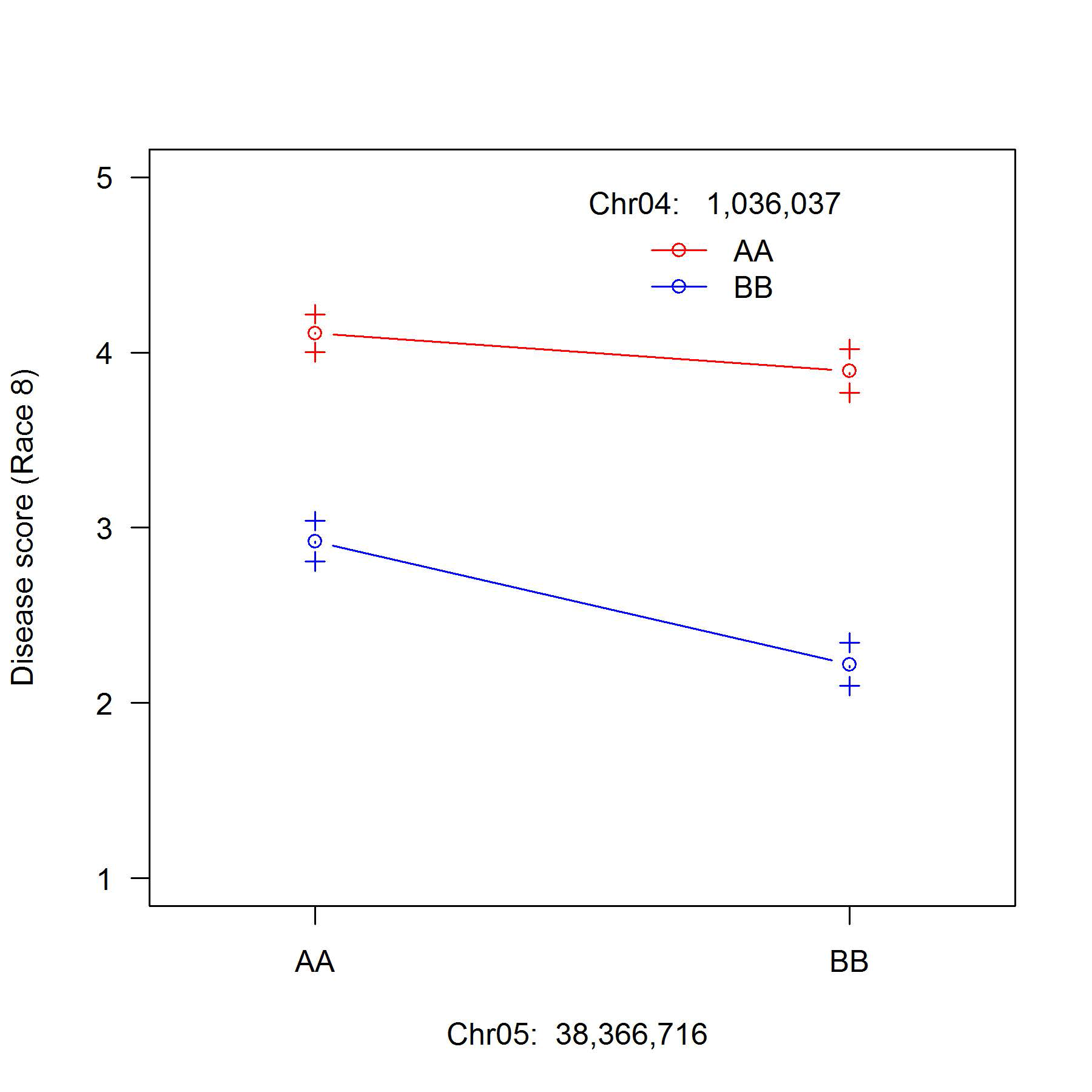


**Figure S3.** Additive and interactive relationships between the major-effect QTL on Pv04 and the minor-effect QTL on Pv05 conditioning resistance to **(A)** Race 6 and **(B)** Race 8 of *Pseudomonas syringae* pv. *phaseolicola* in the *Phaseolus vulgaris* Canadian Wonder × PI 150414 recombinant inbred population. AA: Canadian Wonder (susceptible) parental genotype; BB: PI 150414 (resistant) parental genotype.

**A**


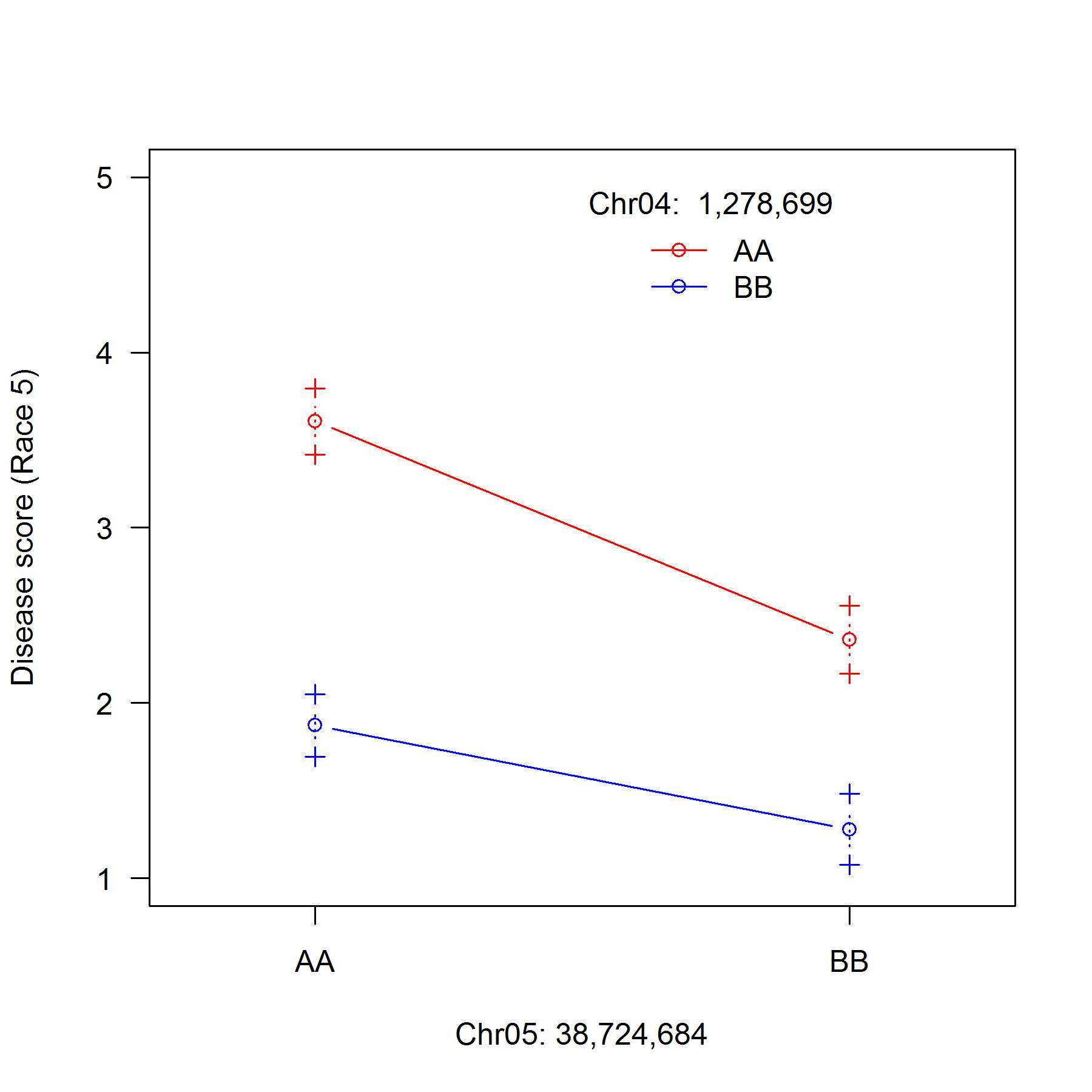


**B**


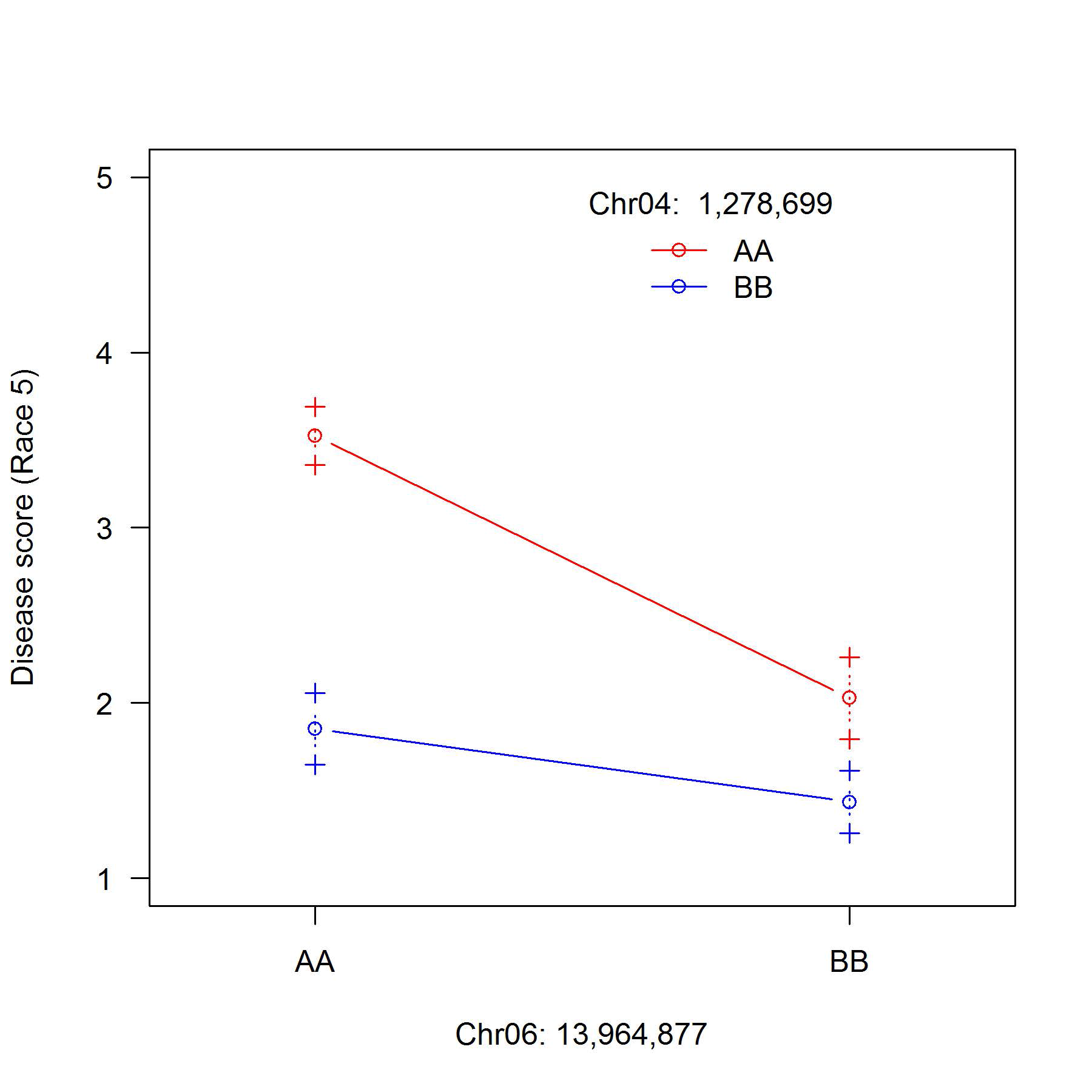


**Figure S4.** Additive and interactive relationships between the major-effect QTL on Pv04 and the minor-effect QTL on **(A)** Pv05 and **(B)** Pv06 conditioning resistance to *Pseudomonas syringae* pv. *phaseolicola* Race 5 in the *Phaseolus vulgaris* Canadian Wonder × PI 150414 recombinant inbred population. AA: Canadian Wonder (susceptible) parental genotype; BB: PI 150414 (resistant) parental genotype.

**A**


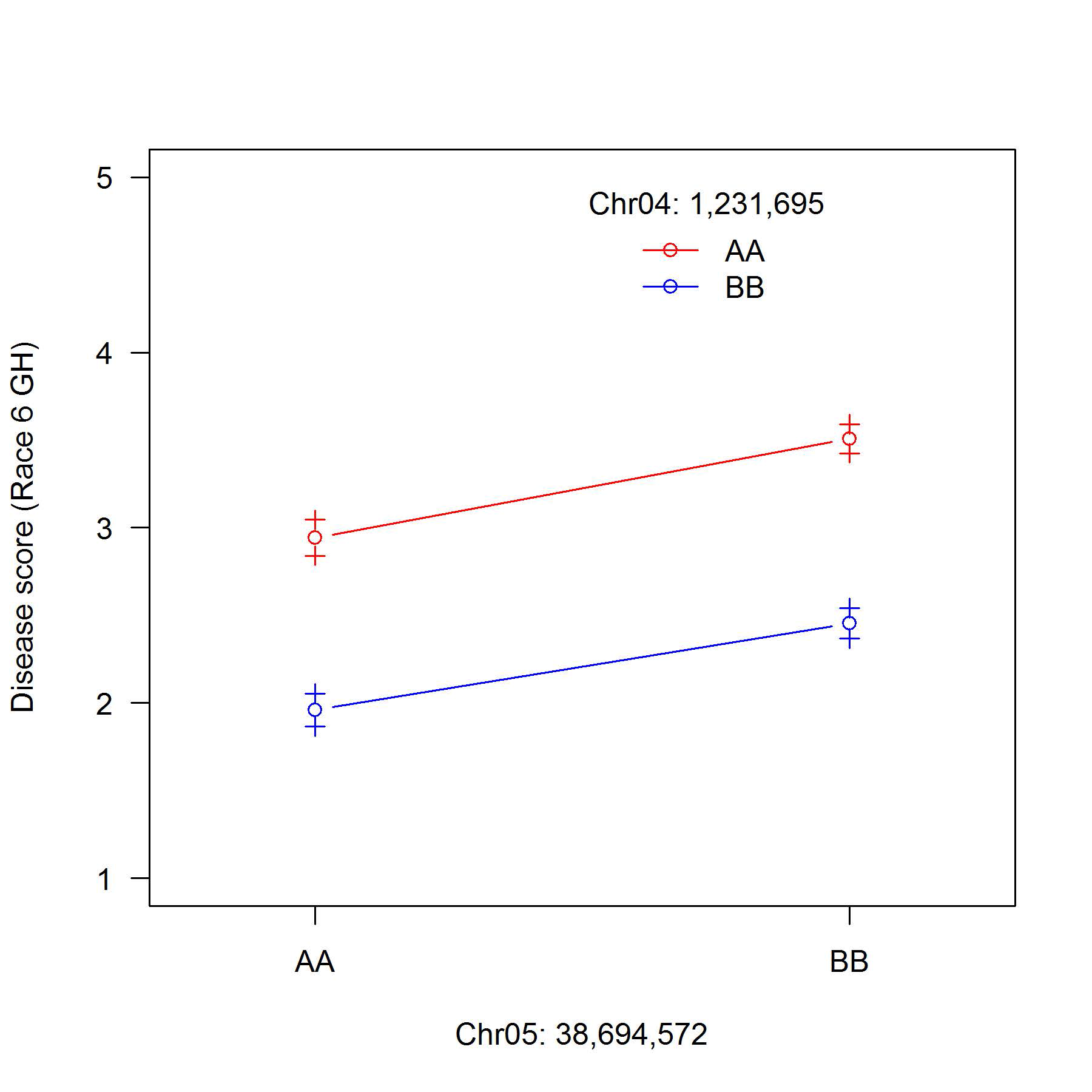


**B**


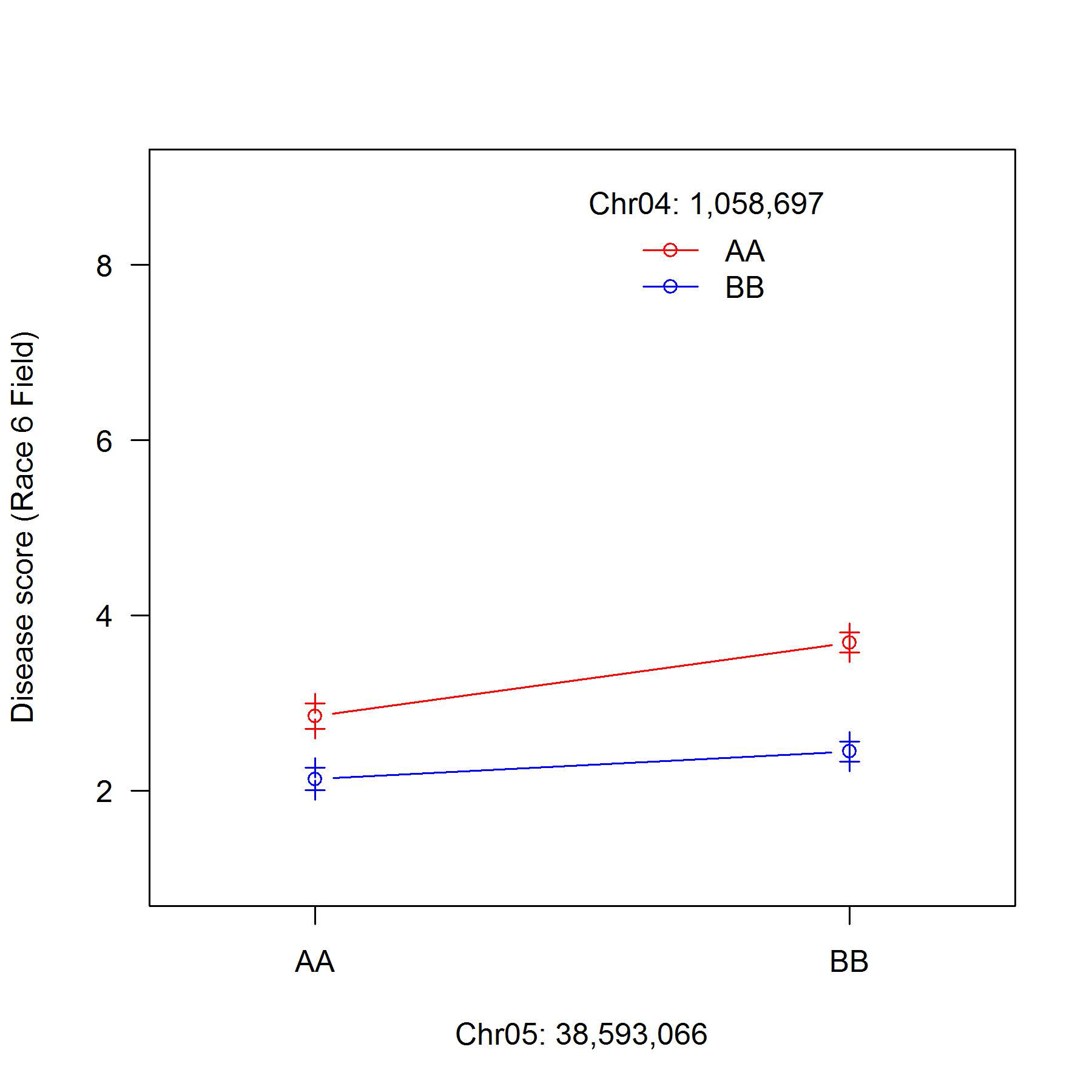


**Figure S5.** Additive relationships between the major-effect QTL on Pv04 (CAL 143-derived) and the minor-effect QTL on Pv05 (Rojo-derived) conditioning **(A)** greenhouse resistance and **(B)** field resistance to *Pseudomonas syringae* pv. *phaseolicola* Race 6 in the *Phaseolus vulgaris* Rojo × CAL 143 recombinant inbred population. AA: Rojo parental genotype; BB: CAL 143 parental genotype.


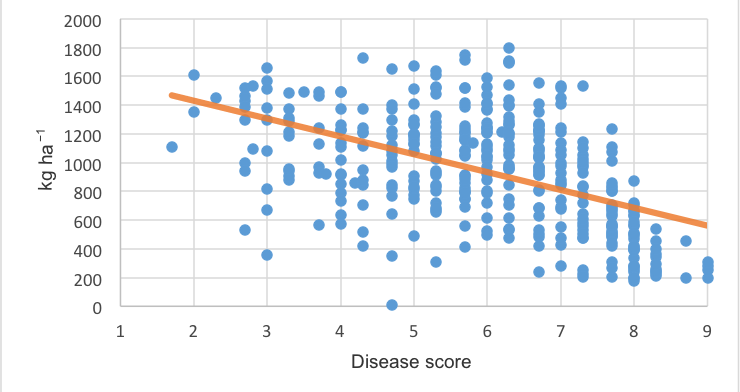


**Figure S6.** Correlation between yield and reaction to *Pseudomonas syringae* pv. *phaseolicola* Race 6 observed in the Andean Diversity Panel of *Phaseolus vulgaris* grown in the field in Potchefstroom, South Africa, during the 2014 growing season (*R* = −0.50; *n* = 384; *P* < 0.0001; *y* = 1608 + −124*x*).

# Supplementary Tables

**Table S1.** Single- and multi-QTL models fitted and refined by multiple imputation for reactions to *Pseudomonas syringae* pv. *phaseolicola* Races 6, 1 and 3 in the *Phaseolus vulgaris* SOA-BN × Edmund recombinant inbred population.

| *Psph* Race | Chr.^a^ | QTL/ gene^b^ | Start | End | Size (Mb) | DF^c^ | SS^d^ | LOD^e^ | *R*^2^ (%)^f^ | Probability (*F*) | | Additive effect |
| --- | --- | --- | --- | --- | --- | --- | --- | --- | --- | --- | --- | --- |
| 6 | 4 | HB4.2 | 599,801 | 1,104,545 | 0.50 | 2 | 47.75 | 29.61 | 82.99 | 0 | *** | -0.78 |
| 1 | 4 | HB4.2 | 599,801 | 1,104,545 | 0.50 | 2 | 43.59 | 38.86 | 83.31 | < 2E-16 | *** | -0.75 |
|  | 8 | HB8.1 | 57,955,365 | 58,844,117 | 0.89 | 2 | 1.57 | 4.88 | 3.00 | 2.78E-05 | *** | -0.13 |
| 3 | 2 | *Pse-3* | 48,398,083 | 48,533,537 | 0.14 | 6 | 12.86 | 53.43 | 95.62 | < 2E-16 | *** | 0.45 |
|  | 9 | HB9.1 | 22,811,364 | 25,010,084 | 2.20 | 6 | 2.69 | 30.79 | 20.00 | < 2E-16 | *** | -0.02 |
|  | 8 | HB8.2 | 2,574,918 | 3,054,024 | 0.48 | 2 | 1.47 | 23.05 | 10.92 | < 2E-16 | *** | 0.02 |
|  | 2 × 9 | *Pse-3* × HB9.1 | N/A | N/A | N/A | 4 | 2.57 | 30.18 | 19.11 | < 2E-16 | *** | N/A |

^a^Chrom.: chromosome; ^b^1.8-LOD QTL support interval in the common bean reference genome (Schmutz et al., 2014); ^c^DF: degrees of freedom; ^d^SS: sum of squares; ^e^LOD: logarithm (base 10) of the odds score; ^f^*R*^2^ (%): percentage of phenotypic variation explained by each locus. ***: significant at the 0.001 probability level.

**Table S2.** Thirty-eight candidate genes located within the 500-kb mapping interval defined for HB4.2 QTL conferring quantitative resistance to multiple races of *Pseudomonas syringae* pv. *phaseolicola* on Pv04 of the *Phaseolus vulgaris* v1.0 reference genome (G19833).

| Annotated gene ID | Location (Phytozome) | Predicted function^a^ | *Arabidopsis thaliana* homolog (BLASTP) |
| --- | --- | --- | --- |
| Phvul.004G007500 | Chr04: 597278–601006 | Protein/domain of unknown function (DUF1421) | AT5G14540 – Protein/domain of unknown function (DUF1421) |
| Phvul.004G007600 | Chr04: 605953–613920 | RNA recognition motif (RRM)-containing protein | AT3G27700 (< 50%) – Zinc finger (CCCH-type) family protein / RNA recognition motif (RRM)-containing protein |
| Phvul.004G007700 | Chr04: 623268–626839 | Disease resistance protein (nucleotide-binding site–leucine-rich repeat protein with putative coiled-coil N-terminal domain, CC–NLR) | AT3G14470 (< 50%) – Nucleotide-binding adapter shared by APAF-1, Resistance proteins, and CED-4 (NB-ARC) domain-containing disease resistance proteins |
| Phvul.004G007800 | Chr04: 643296–647667 | No annotations | AT5G46260 (< 50%) – Disease resistance protein with Toll and interleukin-1 receptor-like N-terminal domain (TIR–NLR) |
| Phvul.004G007900 | Chr04: 672127–676700 | Disease resistance protein (CC–NLR); pseudogene in reference genome | AT3G14470 (< 50%) – NB-ARC domain-containing disease resistance protein |
| Phvul.004G008000 | Chr04: 678195–680885 | Disease resistance protein (CC–NLR); pseudogene in reference genome | AT3G14470 (< 50%) – NB-ARC domain-containing disease resistance protein |
| Phvul.004G008100 | Chr04: 688650–693324 | Disease resistance protein (CC–NLR); pseudogene in reference genome | AT3G14470 (< 50%) – NB-ARC domain-containing disease resistance protein |
| Phvul.004G008200 | Chr04: 707064–709398 | Disease resistance protein (CC–NLR); pseudogene in reference genome | AT3G14470 (< 50%) – NB-ARC domain-containing disease resistance protein |
| Phvul.004G008300 | Chr04: 734268–740298 | Disease resistance protein (CC–NLR) | AT3G14470 (< 50%) – NB-ARC domain-containing disease resistance protein |
| Phvul.004G008400 | Chr04: 750857–752692 | Disease resistance protein (CC–NLR) | AT3G14470 (< 50%) – NB-ARC domain-containing disease resistance protein |
| Phvul.004G008500 | Chr04: 759676–761499 | No annotations | AT5G40400 – Pentatricopeptide repeat (PPR)-containing protein |
| Phvul.004G008600 | Chr04: 765416–768590 | Phospholipid-translocating ATPase | AT3G27870 – ATPase E1-E2 type family |
| Phvul.004G008700 | Chr04: 792116–795536 | Disease resistance protein (CC–NLR) | AT3G14470 (< 50%) – NB-ARC domain-containing disease resistance protein |
| Phvul.004G008800 | Chr04: 824480–828899 | Disease resistance protein (CC–NLR) | AT3G14470 (< 50%) – NB-ARC domain-containing disease resistance protein |
| Phvul.004G008900 | Chr04: 838998–842360 | Disease resistance protein (CC–NLR) | AT3G14470 (< 50%) – NB-ARC domain-containing disease resistance protein |
| Phvul.004G009000 | Chr04: 852372–853227 | No annotations | AT3G27670 – Armadillo/β-catenin-like repeat protein |
| Phvul.004G009100 | Chr04: 853293–853832 | Leucine-rich repeat (LRR)-containing protein | AT3G14470 (< 50%) – NB-ARC domain-containing disease resistance protein |
| Phvul.004G009200 | Chr04: 856940–868944 | Protein/domain of unknown function (DUF3730) | AT3G27670 – Armadillo/β-catenin-like repeat protein |
| Phvul.004G009300 | Chr04: 873737–877733 | Disease resistance protein (CC–NLR) | AT3G14470 (< 50%) – NB-ARC domain-containing disease resistance protein |
| Phvul.004G009400 | Chr04: 892792–894806 | E3 UBIQUITIN-PROTEIN LIGASE 6 (UPL6) | AT3G17205 – E3 UBIQUITIN-PROTEIN LIGASE 6 (UPL6) |
| Phvul.004G009500 | Chr04: 896498–901075 | Disease resistance protein (CC–NLR) | AT3G14470 (< 50%) – NB-ARC domain-containing disease resistance protein |
| Phvul.004G009600 | Chr04: 906782–909895 | No annotations | AT3G27870 – ATPase E1-E2 type family |
| Phvul.004G009700 | Chr04: 945326–955745 | No annotations | AT3G27670 – Armadillo/β-catenin-like repeat protein |
| Phvul.004G009800 | Chr04: 966792–971111 | Disease resistance protein (CC–NLR) | AT3G14470 (< 50%) – NB-ARC domain-containing disease resistance protein |
| Phvul.004G009900 | Chr04: 976122–979478 | PPR-containing protein | AT5G40400 – PPR-containing protein |
| Phvul.004G010000 | Chr04: 982581–992206 | Protein/domain of unknown function (DUF3730) | AT3G27670 – Armadillo/β-catenin-like repeat protein |
| Phvul.004G010100 | Chr04: 999365–1001208 | LATERAL ORGAN BOUNDARIES DOMAIN-CONTAINING PROTEIN 25 (LBD25) | AT3G27650 – LATERAL ORGAN BOUNDARIES DOMAIN-CONTAINING PROTEIN 25 (LBD25) |
| Phvul.004G010200 | Chr04: 1006379–1006744 | Plant self-incompatibility protein S1 | AT4G16195 (< 50%) – Plant self-incompatibility protein S1 |
| Phvul.004G010300 | Chr04: 1010279–1010683 | Plant self-incompatibility protein S1 | AT4G16195 (< 50%) – Plant self-incompatibility protein S1 |
| Phvul.004G010400 | Chr04: 1015240–1019248 | Mitogen-activated protein kinase kinase (MAPKK); Serine/threonine-protein kinase STE7 | AT5G40440 – Mitogen-activated protein kinase kinase 3 (MKK3) |
| Phvul.004G010500 | Chr04: 1019873–1024420 | WD40 REPEAT-CONTAINING PROTEIN L2DTL | AT3G27640 (< 50%) – Transducin/WD40 repeat-like superfamily protein |
| Phvul.004G010600 | Chr04: 1025421–1029236 | CLEAVAGE/POLYADENYLATION FACTOR 1 (CLP1) | AT5G11010 – Pre-mRNA cleavage complex II protein family |
| Phvul.004G010700 | Chr04: 1035453–1036874 | Cyclin-dependent protein kinase inhibitor (SMR2-related) | AT5G40460 – Protein/domain of unknown function |
| Phvul.004G010800 | Chr04: 1053217–1056560 | Cobalamin (vitamin B12) biosynthesis (CbiX) protein | AT1G50170 – SIROHYDROCHLORIN FERROCHELATASE B |
| Phvul.004G010900 | Chr04: 1057410–1060763 | No annotations | AT1G12700 (< 50%) – RNA PROCESSING FACTOR 1 (RPF1, a PPR); ATP binding; nucleic acid binding; helicases |
| Phvul.004G011000 | Chr04: 1084516–1089721 | CYSTEINE-RICH RECEPTOR-LIKE PROTEIN KINASE 42 (CRK42); salt stress response/antifungal; protein tyrosine kinase; serine/threonine protein kinase | AT5G40380 – CYSTEINE-RICH RECEPTOR-LIKE PROTEIN KINASE 42 (CRK42) |
| Phvul.004G011100 | Chr04: 1093516–1103190 | Polyribonucleotide nucleotidyltransferase | AT5G14580 – Polyribonucleotide nucleotidyltransferase |
| Phvul.004G011200 | Chr04: 1104370–1113109 | Nicotinamide adenine dinucleotide phosphate (NADP)-specific isocitrate dehydrogenase (IDH) | AT5G14590 – Isocitrate/isopropylmalate dehydrogenase (IDH/IMDH) family protein |

^a^Predicted protein functional annotation in the *Phaseolus vulgaris* v1.0 reference genome (genotype G19833) as identified by Phytozome version 11.0 (PANTHER/Pfam/EuKaryotic Orthologous Groups/National Center for Biotechnology Information) (Goodstein *et al.*, 2012).

**Table S3.** Single- and multi-QTL models fitted and refined by multiple imputation for reactions to nine races of *Pseudomonas syringae* pv. *phaseolicola* in the *Phaseolus vulgaris* Canadian Wonder × PI 150414 recombinant inbred population. Abbreviations are as described for Table S1.

| *Psph* Race | Chr. | QTL/ gene | Start | End | Size (Mb) | DF | SS | LOD | *R*^2^ (%) | Probability (*F*) | | Additive effect |
| --- | --- | --- | --- | --- | --- | --- | --- | --- | --- | --- | --- | --- |
| 1 | 4 | HB4.2 | 218,204 | 1,134,467 | 0.92 | 1 | 38.23 | 17.66 | 74.21 | 0 | *** | -0.78 |
| 2 | 4 | HB4.2 | 487,659 | 1,134,467 | 0.65 | 1 | 50.92 | 18.49 | 76.96 | 0 | *** | -0.92 |
| 3+4 | 4 | HB4.2 | 1,036,037 | 1,134,467 | 0.1 | 1 | 56.12 | 28.52 | 88.79 | 0 | *** | -0.93 |
| 5 | 4 | HB4.2 | 218,204 | 1,485,804 | 1.27 | 3 | 29.28 | 13.84 | 39.48 | 1.70E−12 | *** | -0.59 |
|  | 5 | HB5.1 | 38,245,197 | 40,400,058 | 2.15 | 2 | 12.19 | 7.57 | 16.44 | 1.50E−07 | *** | -0.40 |
|  | 6 | HB6.1 | 174,972 | 15,786,595 | 15.61 | 2 | 13.26 | 8.06 | 17.88 | 5.50E−08 | *** | -0.41 |
|  | 4 × 5 | HB4.2 × HB5.1 | N/A | N/A | N/A | 1 | 1.25 | 1.01 | 1.68 | 4.20E-02 | * | 0.11 |
|  | 4 × 6 | HB4.2 × HB6.1 | N/A | N/A | N/A | 1 | 4.21 | 3.14 | 5.68 | 3.30E-04 | *** | 0.24 |
| 6 | 4 | HB4.2 | 218,204 | 1,485,804 | 1.27 | 2 | 26.78 | 15.82 | 64.65 | 1.70E-15 | *** | -0.65 |
|  | 5 | HB5.1 | 21,052 | 40,078,248 | 40.06 | 2 | 8.45 | 7.27 | 20.41 | 1.60E-07 | *** | -0.31 |
|  | 4 × 5 | HB4.2 × HB5.1 | N/A | N/A | N/A | 1 | 2.66 | 2.76 | 6.43 | 6.10E-04 | *** | -0.20 |
| 7 | 4 | HB4.2 | 1,134,467 | 1,485,804 | 0.35 | 1 | 55.97 | 19.26 | 79.49 | 0 | *** | -0.98 |
| 8 | 4 | HB4.2 | 218,204 | 1,485,804 | 1.27 | 2 | 29.83 | 16.86 | 67.44 | 2.20E-16 | *** | -0.71 |
|  | 5 | HB5.1 | 21,052 | 40,400,058 | 40.38 | 2 | 4.11 | 4.08 | 9.28 | 1.50E-04 | *** | -0.23 |
|  | 4 × 5 | HB4.2 × HB5.1 | N/A | N/A | N/A | 1 | 0.91 | 1.02 | 2.06 | 3.60E-02 | * | -0.12 |
| 9 | 4 | HB4.2 | 487,659 | 1,134,467 | 0.65 | 1 | 31.89 | 16.7 | 72.85 | 0 | *** | -0.73 |

**Table S4.** Single and multi-QTL models fitted and refined by multiple imputation for reaction to eight races of *Pseudomonas syringae* pv. *phaseolicola* in the *Phaseolus vulgaris* Rojo × CAL 143 RIL population. Abbreviations are as described for Table S1.

| *Psph* Race | Chr. | QTL/ gene | Start | End | Size (Mb) | DF | SS | LOD | *R*^2^ (%) | Probability (*F*) | | Additive effect | |
| --- | --- | --- | --- | --- | --- | --- | --- | --- | --- | --- | --- | --- | --- |
| 1 | 4 | HB4.2 | 225,825 | 1,308,175 | 1.08 | 1 | 63.10 | 63.10 | 37.20 | 0 | *** | | -0.66 |
| 2 | 4 | HB4.2 | 225,825 | 1,058,697 | 0.83 | 2 | 73.60 | 36.60 | 54.90 | < 2E-16 | *** | | -0.53 |
|  | 10 | *Pse-2* | 3,411,531 | 3,457,673 | 0.05 | 2 | 74.10 | 36.70 | 55.30 | < 2E-16 | *** | | 0.57 |
|  | 4 × 10 | HB4.2 × *Pse-2* | N/A | N/A | N/A | 1 | 34.00 | 22.10 | 25.40 | < 2E-16 | *** | | -0.47 |
| 3 | 2 | *Pse-3* | 47,368,363 | 48,295,243 | 0.93 | 2 | 85.90 | 60.90 | 79.20 | < 2E-16 | *** | | 0.58 |
|  | 10 | *Pse-2* | 5,151,238 | 8,601,504 | 3.45 | 2 | 64.30 | 53.40 | 59.30 | < 2E-16 | *** | | 0.56 |
|  | 2 × 10 | *Pse-3* × *Pse-2* | N/A | N/A | N/A | 1 | 50.50 | 47.50 | 46.60 | < 2E-16 | *** | | 0.57 |
| 5 | 10 | *Pse-2* | 3,562,199 | 39,267,273 | 35.71 | 1 | 3.87 | 3.87 | 3.350 | 9.87E-05 | *** | | 0.16 |
| 6 (GH) | 4 | HB4.2 | 225,825 | 1,468,815 | 1.24 | 2 | 37.50 | 19.90 | 40.60 | < 2E-16 | *** | | -0.51 |
|  | 5 | HB5.1 | 37,438,027 | 40,521,638 | 3.08 | 2 | 9.80 | 6.60 | 10.6 | 3.80E-07 | *** | | 0.27 |
| 6  (Field) | 4 | HB4.2 | 54,325 | 1,468,815 | 1.41 | 2 | 40.80 | 13.30 | 30.60 | 1.03E-13 | *** | | -0.49 |
|  | 5 | HB5.1 | 34,671,030 | 40,521,638 | 5.85 | 2 | 13.40 | 5.00 | 10.00 | 1.25E-05 | *** | | 0.29 |
| 7 | 4 | HB4.2 | 54,325 | 2,112,520 | 2.06 | 2 | 8.70 | 11.40 | 15.00 | 1.06E-11 | *** | | -0.17 |
|  | 6 | HB6.2 | 346,023 | 14,964,963 | 14.62 | 2 | 11.10 | 13.90 | 19.10 | 4.40E-14 | *** | | 0.21 |
|  | 10 | *Pse-2* | 2,808,356 | 3,595,005 | 0.79 | 3 | 30.20 | 28.80 | 51.70 | < 2E-16 | *** | | 0.37 |
|  | 4 × 10 | HB4.2 × HB6.2 | N/A | N/A | N/A | 1 | 4.70 | 6.70 | 8.10 | 5.17E-08 | *** | | -0.18 |
|  | 6 × 10 | HB6.2 × *Pse-2* | N/A | N/A | N/A | 1 | 4.21 | 6.00 | 7.20 | 2.67E-07 | *** | | 0.16 |
| 8 | 4 | HB4.2 | 225,825 | 1,058,697 | 0.83 | 2 | 65.10 | 61.70 | 57.60 | < 2E-16 | *** | | -0.46 |
|  | 10 | *Pse-2* | 3,411,531 | 3,457,673 | 0.05 | 2 | 85.70 | 69.30 | 75.80 | < 2E-16 | *** | | 0.63 |
|  | 4 × 10 | HB4.2 × *Pse-2* | N/A | N/A | N/A | 1 | 34.50 | 45.50 | 30.50 | < 2E-16 | *** | | -0.45 |
| 9 | 4 | HB4.2 | 225,825 | 1,468,815 | 1.24 | 2 | 34.00 | 24.00 | 41.90 | < 2E-16 | *** | | -0.37 |
|  | 10 | *Pse-2* | 3,411,531 | 4,416,547 | 1.01 | 2 | 40.50 | 27.00 | 49.90 | < 2E-16 | *** | | 0.44 |
|  | 4 × 10 | HB4.2 × *Pse-2* | N/A | N/A | N/A | 1 | 15.30 | 13.20 | 18.80 | 1.71E-14 | *** | | -0.33 |

**Table S5.**  Three hundred and eighty-four accessions of the *Phaseolus vulgaris* Andean Diversity Panel sorted by disease score (rated on a 1.0 to 9.0 scale) following inoculation with *Pseudomonas syringae* pv. *phaseolicola* Race 6 in the field in Potchefstroom, South Africa, in 2014.

| ADP ID | Genotype | Type | Habit | Origin | HB score 1–9 (least squares mean) | Yield (kg ha^−1^) | I gene | HB5.1 SNP S5_38725023 |
| --- | --- | --- | --- | --- | --- | --- | --- | --- |
| ADP0121 | Kranskop HR-1 | cran | Vine | Africa | 1.7 | 1111 | I gene | G |
| ADP0118 | Werna | cran | Vine | Africa | 2.0 | 1356 | I gene | G |
| ADP0797 | RS 7 | CRAN | Vine | HBB resistant | 2.0 | 1611 | I gene | G |
| ADP0716 | SUGAR 131 | CRAN | Vine | E Africa | 2.3 | 1450 | I gene | G |
| ADP0084 | KABLANKETI NDEFU | drk purp spec | Ag.Climber | Africa | 2.7 | 994 | No I gene | G |
| ADP0125 | CHEUPE | white | Ag. Climber | Africa | 2.7 | 533 | ND | G |
| ADP0255 | G 10994 | tan | Ag.Climber | CIAT core | 2.7 | 1428 | No I gene | G |
| ADP0276 | G 13654 | tan | Vine | CIAT core | 2.7 | 939 | No I gene | G |
| ADP0454 | INIAP 429 | red mottled | Ag.Climber | Ecuador | 2.7 | 1467 | No I gene | G |
| ADP0723 | KAB 06 F2.8-69 | CRAN | Vine | E Africa | 2.7 | 1389 | ND | G |
| ADP0734 | GCI-CAL-272-AR-1 | RD MOTT | Bush | E Africa | 2.7 | 1522 | ND | G |
| ADP0526 | CAL 143 | red mottled | Bush | CIAT Africa | 2.7 | 1294 | ND | T |
| ADP0428 | Colorado del Pais | pink cran | Bush | Carribean | 2.8 | 1094 | ND | G |
| ADP0737 | KG 27-8 | RD MOTT | Vine | E Africa | 2.8 | 1533 | ND | G |
| ADP0080 | KABLANKETI | purp spec | Vine | Africa | 3.0 | 1567 | No I gene | G |
| ADP0114 | OPS-RS1 | cran | Vine | Africa | 3.0 | 1378 | I gene | G |
| ADP0120 | Tygerberg | cran | Vine | Africa | 3.0 | 672 | I gene | G |
| ADP0432 | PR0637-134 | red mottled | Bush | Carribean | 3.0 | 1078 | NS | G |
| ADP0566 | G5686 | yellow mottled | Vine | CIAT Africa | 3.0 | 817 | No I gene | G |
| ADP0592 | AND 1005 | red mottled | Vine | CIAT Africa | 3.0 | 1656 | ND | G |
| ADP0798 | Kamiesberg | CRAN | Vine | Rust resistant | 3.0 | 356 | I gene | G |
| ADP0731 | BILFA UYOLE | RD MOTT | Bush | E Africa | 3.0 | 1294 | ND | T |
| ADP0740 | CIM-RM05-ALS-82 | RD MOTT | Vine | E Africa | 3.0 | 1511 | ND | T |
| ADP0122 | Kranskop | cran | Vine | Africa | 3.3 | 1183 | I gene | G |
| ADP0123 | Jenny | cran | Vine | Africa | 3.3 | 1289 | I gene | G |
| ADP0379 | PI 203934 | cream | Bush | US core | 3.3 | 878 | No I gene | G |
| ADP0508 | Calembe | manteca | Vine | Angola | 3.3 | 1311 | No I gene | G |
| ADP0537 | AFR 619 | red mottled | Bush | CIAT Africa | 3.3 | 1483 | No I gene | G |
| ADP0572 | NUA56 | d red mottled | Vine | CIAT Africa | 3.3 | 939 | No I gene | G |
| ADP0728 | GCI -LRK-291-RAR | LRK | Vine | E Africa | 3.3 | 906 | No I gene | G |
| ADP0736 | KG 15-26 | RD MOTT | Vine | E Africa | 3.3 | 1372 | ND | G |
| ADP0739 | UYOLE 03 | TAN/PUR | Bush | E Africa | 3.3 | 1211 | ND | G |
| ADP0732 | GCI-CAL-270-AR-3 | RD MOTT | Bush | E Africa | 3.3 | 956 | ND | T |
| ADP0730 | GCI-CAL-272-AR-2 | PUR MOTT | Vine | E Africa | 3.5 | 1494 | ND | T |
| ADP0082 | KABLANKETI | purp spec | Ag.Climber | Africa | 3.7 | 567 | ND | G |
| ADP0433 | PR9745-232 | red mottled | Bush | Carribean | 3.7 | 967 | I gene | G |
| ADP0541 | CIM 9314-36 |  | Bush | CIAT Africa | 3.7 | 1461 | No I gene | G |
| ADP0597 | G23829 | black | Vine | CIAT Africa | 3.7 | 1133 | seg | G |
| ADP0721 | DRK 57 | DRK | Vine | E Africa | 3.7 | 1242 | ND | G |
| ADP0735 | KG 27-13 | RD MOTT | Vine | E Africa | 3.7 | 1489 | ND | G |
| ADP0555 | BRB191 | red mottled | Bush | CIAT Africa | 3.7 | 928 | ND | T |
| ADP0654 | USDK-4 | DRK | Bush | N American | 3.8 | 922 | NS | T |
| ADP0008 | Nyayo | red mottled | Bush | Africa | 4.0 | 850 | No I gene | G |
| ADP0030 | RH No. 6 | blk | Bush | Africa | 4.0 | 917 | I gene | G |
| ADP0055 | KABUKU | red spec | Vine | Africa | 4.0 | 1226 | No I gene | G |
| ADP0109 | Kablanketi | pur mot | Vine | Africa | 4.0 | 572 | No I gene | G |
| ADP0113 | OPS-RS4 | cran | Vine | Africa | 4.0 | 789 | I gene | G |
| ADP0119 | A193 | spec red | Vine | Africa | 4.0 | 1017 | No I gene | G |
| ADP0192 | G 2377 | tan | Vine | CIAT core | 4.0 | 1372 | No I gene | G |
| ADP0272 | G 13336 | purp cran | Ag.Climber | CIAT core | 4.0 | 1261 | No I gene | G |
| ADP0395 | PI 310511 | red mottled | Bush | US core | 4.0 | 922 | No I gene | G |
| ADP0430 | PR1013-3 | pink mottled | Bush | Carribean | 4.0 | 1228 | No I gene | G |
| ADP0442 | Larga Comercial | red mottled | Vine | Carribean | 4.0 | 1106 | ND | G |
| ADP0531 | AND 620 | d red mottled | Bush | CIAT Africa | 4.0 | 733 | I gene | G |
| ADP0574 | RADICAL CERINZA |  | Bush | CIAT Africa | 4.0 | 633 | ND | G |
| ADP0632 | TARS HT 1 | DRK | Bush | N American | 4.0 | 1139 | I gene | G |
| ADP0117 | A483 | pur mot | Vine | Africa | 4.0 | 1489 | I gene | T |
| ADP0738 | KG 27-26 | RD MOTT | Vine | E Africa | 4.0 | 1494 | ND | T |
| ADP0510 | Ohlio de perdiz | Jacobs Cattle | Vine | Angola | 4.2 | 861 | No I gene | T |
| ADP0061 | Maulasi | brn cran | Bush | Africa | 4.3 | 1727 | No I gene | G |
| ADP0081 | KABLANKETI | purp spec | Vine | Africa | 4.3 | 844 | No I gene | G |
| ADP0089 | KABLANKETI | purp spec | Vine | Africa | 4.3 | 422 | No I gene | G |
| ADP0211 | G 4780 | red mottled | Bush | CIAT core | 4.3 | 517 | No I gene | G |
| ADP0520 | Chumbo, Cela | green | Vine | Angola | 4.3 | 1211 | No I gene | G |
| ADP0551 | AFR 612 | d. red mottled | Bush | CIAT Africa | 4.3 | 706 | ND | G |
| ADP0590 | SEQ11 | d. purple mottled | Vine | CIAT Africa | 4.3 | 1372 | ND | G |
| ADP0647 | Red Kanner | LRK | Bush | N American | 4.3 | 950 | I gene | G |
| ADP0719 | NUA 59 | PUR MOTT | Vine | E Africa | 4.3 | 878 | No I gene | G |
| ADP0720 | PAN 148 | CRAN | Vine | E Africa | 4.3 | 1244 | I gene | G |
| ADP0794 | Sederberg | CRAN | Vine |  | 4.3 | 1117 | I gene | G |
| ADP0796 | RS 6 | CRAN | Vine |  | 4.3 | 1206 | ND | G |
| ADP0009 | Maalasa | red mottled | Bush | Africa | 4.7 | 1083 | No I gene | G |
| ADP0035 | Kokola | red mottled | Bush | Africa | 4.7 | 1056 | No I gene | G |
| ADP0036 | Lyamungu 85 | red mottled | Bush | Africa | 4.7 | 1189 | No I gene | G |
| ADP0056 | SOYA | purp spec | Vine | Africa | 4.7 | 1122 | No I gene | G |
| ADP0088 | KABLANKETI | purp spec | Vine | Africa | 4.7 | 639 | No I gene | G |
| ADP0115 | Bonus | cran | Vine | Africa | 4.7 | 844 | No I gene | G |
| ADP0427 | Badillo | red kidney | Same as ADP-626 | Carribean | 4.7 | 1011 | I gene | G |
| ADP0462 | IZ 117 | dull yellow | Ag.Climber | East Africa | 4.7 | 350 | No I gene | G |
| ADP0545 | PAN 127 | CRAN | Vine | CIAT Africa | 4.7 | 1222 | No I gene | G |
| ADP0631 | OAC Inferno | LRK | Bush | N American | 4.7 | 767 | ND | G |
| ADP0722 | KAB 06 F2.8-144 | CRAN | Vine | E Africa | 4.7 | 1372 | I gene | G |
| ADP0726 | INTER LAB 06 | CRAN | Vine | E Africa | 4.7 | 1650 | I gene | G |
| ADP0751 | Kabanima Nyeupe | red mottled | Bush | E Africa | 4.7 | 822 | ND | G |
| ADP0127 | SELIAN 06 | pink | Ag. Climber | Africa | 4.7 | 11 | No I gene | T |
| ADP0527 | POA 2 | red mottled | Bush | CIAT Africa | 4.7 | 1400 | No I gene | T |
| ADP0660 | Krimson | CRAN | Bush | N American | 4.7 | 1300 | I gene | T |
| ADP0665 | USWK-CBB-17 | WK | Bush | N American | 4.7 | 989 | I gene | T |
| ADP0748 | Rozi Koko | pink mottled | Vine | E Africa | 4.7 | 972 | ND | T |
| ADP0001 | ROZI KOKO | red spec | Bush | Africa | 5.0 | 1671 | No I gene | G |
| ADP0063 | Soya | purp. Spec | Ag.Climber | Africa | 5.0 | 489 | No I gene | G |
| ADP0076 | KABLANKETI | purp spec | Vine | Africa | 5.0 | 814 | No I gene | G |
| ADP0110 | SUG-131 | cran | Vine | Africa | 5.0 | 906 | No I gene | G |
| ADP0225 | G 6415 | LRK | Vine ? Should be bush | CIAT core | 5.0 | 772 | I gene | G |
| ADP0337 | G 21303 |  | Ag.Climber | CIAT core | 5.0 | 1200 | ND | G |
| ADP0455 | INIAP 430 | red mottled | Bush | Ecuador | 5.0 | 1133 | No I gene | G |
| ADP0465 | Bosha | cream | Vine | East Africa | 5.0 | 1200 | seg | G |
| ADP0513 | Canario | yellow | Vine | Angola | 5.0 | 1178 | No I gene | G |
| ADP0615 | Litekid | LRK | Bush | N American | 5.0 | 1083 | No I gene | G |
| ADP0621 | Jalo EEP558 | Yellow | Vine | N American | 5.0 | 1161 | No I gene | G |
| ADP0636 | Montcalm | DRK | Bush | N American | 5.0 | 1194 | I gene | G |
| ADP0649 | Kamiakin | LRK | Bush | N American | 5.0 | 1411 | I gene | G |
| ADP0744 | RH No 14 | pink mottled | Vine | E Africa | 5.0 | 1261 | ND | G |
| ADP0086 | NYAMHONGA MWEKUNDU | purp spec | Ag.Climber | Africa | 5.0 | 744 | No I gene | T |
| ADP0166 | NABE 4 | red mottled | Bush | Africa | 5.0 | 1300 | No I gene | T |
| ADP0434 | PR0737-1 | red mottled | Vine | Carribean | 5.0 | 1267 | I gene | T |
| ADP0530 | SELIAN 94 | pink mottled | Vine | CIAT Africa | 5.0 | 1128 | No I gene | T |
| ADP0544 | PVA 773 | red mottled | Bush | CIAT Africa | 5.0 | 872 | No I gene | T |
| ADP0717 | VTTT 924/4-4 | CRAN | Vine | E Africa | 5.0 | 833 | ND | T |
| ADP0724 | INTER LAB 04 | DRK | Vine | E Africa | 5.0 | 1511 | ND | T |
| ADP0041 | MRONDO | red | Vine | Africa | 5.3 | 1117 | No I gene | G |
| ADP0051 | RH No. 3 | purp spec | Vine | Africa | 5.3 | 672 | No I gene | G |
| ADP0052 | RH No. 9 | purp spec | Ag.Climber | Africa | 5.3 | 656 | ND | G |
| ADP0060 | CANADA | red | Vine | Africa | 5.3 | 1067 | No I gene | G |
| ADP0062 | MAULASI | red spec | Vine | Africa | 5.3 | 1178 | No I gene | G |
| ADP0087 | KABLANKETI | purp spec | Vine | Africa | 5.3 | 911 | No I gene | G |
| ADP0096 | Rojo | red | Bush | Africa | 5.3 | 939 | I gene | G |
| ADP0346 | G 22246 | red mottled | Vine | CIAT core | 5.3 | 800 | ND | G |
| ADP0368 | G 23093 | white pinkish | Vine | CIAT core | 5.3 | 1283 | No I gene | G |
| ADP0435 | RM-05-07 | red mottled | Bush | Carribean | 5.3 | 844 | NS | G |
| ADP0438 | 46-1 | red mottled | Vine | Carribean | 5.3 | 717 | ND | G |
| ADP0457 | INIAP 481 | red mottled | Ag.Climber | Ecuador | 5.3 | 683 | No I gene | G |
| ADP0481 | Roko | red/white mottled | Bush | East Africa | 5.3 | 1517 | No I gene | G |
| ADP0523 | Canario, Cela | yellow | Vine | Angola | 5.3 | 1528 | No I gene | G |
| ADP0532 | A 197 | light tan | Bush | CIAT Africa | 5.3 | 1611 | I gene | G |
| ADP0580 | SAB626 | CRAN | Bush | CIAT Africa | 5.3 | 1328 | No I gene | G |
| ADP0586 | SAB691 | CRAN | Bush | CIAT Africa | 5.3 | 1639 | ND | G |
| ADP0626 | Badillo | LRK | Vine | N American | 5.3 | 1028 | I gene | G |
| ADP0639 | Chinook 2000 | LRK | Bush | N American | 5.3 | 928 | I gene | G |
| ADP0651 | K-59 | LRK | Bush | N American | 5.3 | 1206 | I gene | G |
| ADP0741 | RH No 19 | brown | Bush | E Africa | 5.3 | 1061 | ND | G |
| ADP0753 | Magpie | black and white | Bush | E Africa | 5.3 | 829 | ND | G |
| ADP0795 | RS 5 | CRAN | Bush |  | 5.3 | 1478 | No I gene | G |
| ADP0279 | G 14423 | J. cattle | Ag.Climber | CIAT core | 5.3 | 1067 | ND | T |
| ADP0453 | INIAP 428 |  | Vine | Ecuador | 5.3 | 311 | ND | T |
| ADP0564 | AMENDOIN | pink mottled | Vine | CIAT Africa | 5.3 | 1144 | No I gene | T |
| ADP0729 | UYOLE 94 | STRIP PUR | Vine | E Africa | 5.3 | 1250 | ND | T |
| ADP0022 | KISAPURI | red | Bush | Africa | 5.7 | 861 | No I gene | G |
| ADP0045 | RH No. 12 | purp spec | Vine | Africa | 5.7 | 794 | No I gene | G |
| ADP0064 | W6 16500 | yellow | Vine | Africa | 5.7 | 1389 | No I gene | G |
| ADP0069 | SOYA | purp spec | Vine | Africa | 5.7 | 561 | NS | G |
| ADP0074 | KABLANKETI | purp spec | Vine | Africa | 5.7 | 750 | No I gene | G |
| ADP0092 | MORO | yellow | Vine | Africa | 5.7 | 1089 | No I gene | G |
| ADP0476 | Hutterite | yellow | Vine | East Africa | 5.7 | 1050 | No I gene | G |
| ADP0482 | Kibariki | Purple mottled | Bush | East Africa | 5.7 | 1044 | ND | G |
| ADP0516 | Mantega, Kibala | manteca | Vine | Angola | 5.7 | 1517 | No I gene | G |
| ADP0518 | Mantega blanca, Kibala | manteca | Bush | Angola | 5.7 | 911 | No I gene | G |
| ADP0535 | ARA 4 | red mottled | Bush | CIAT Africa | 5.7 | 1156 | No I gene | G |
| ADP0538 | RWR 221 | LRK | Bush | CIAT Africa | 5.7 | 1750 | I gene | G |
| ADP0540 | AFR 708 | red mottled | Bush | CIAT Africa | 5.7 | 1206 | No I gene | G |
| ADP0554 | AND279 | red mottled | Bush | CIAT Africa | 5.7 | 1211 | No I gene | G |
| ADP0556 | BRB194 | d. red | Bush | CIAT Africa | 5.7 | 744 | No I gene | G |
| ADP0562 | DAB387 | CRAN | Bush | CIAT Africa | 5.7 | 1178 | ND | G |
| ADP0581 | SAB629 | CRAN | Bush | CIAT Africa | 5.7 | 806 | I gene | G |
| ADP0582 | SAB630 | CRAN | Bush | CIAT Africa | 5.7 | 1256 | I gene | G |
| ADP0585 | SAB686 | CRAN | Bush | CIAT Africa | 5.7 | 1517 | No I gene | G |
| ADP0599 | Isles | red | Bush | N American | 5.7 | 822 | I gene | G |
| ADP0605 | 1132-V96 | LRK | Bush | N American | 5.7 | 1233 | I gene | G |
| ADP0614 | ND061106 | LRK | Bush | N American | 5.7 | 906 | I gene | G |
| ADP0644 | Fox Fire | LRK | Bush | N American | 5.7 | 1417 | I gene | G |
| ADP0680 | Clouseau | LRK | Bush | N American | 5.7 | 1156 | seg | G |
| ADP0727 | INTER LAB 23 | TAN | Bush | E Africa | 5.7 | 1716 | I gene | G |
| ADP0058 | CANADA | red | Vine | Africa | 5.7 | 1078 | No I gene | T |
| ADP0083 | W6 16547 | purp spec | Ag.Climber | Africa | 5.7 | 694 | No I gene | T |
| ADP0105 | Sewani 97 | red | Bush | Africa | 5.7 | 883 | No I gene | T |
| ADP0471 | IZ 102 | yellow/ brown | Vine | East Africa | 5.7 | 411 | ND | T |
| ADP0570 | NATALSUGAR | CRAN | Vine | CIAT Africa | 5.7 | 789 | ND | T |
| ADP0094 | LUSHALA | yellow | Vine | Africa | 5.8 | 1139 | No I gene | T |
| ADP0007 | BUKOBA | yellow | Bush | Africa | 6.0 | 922 | No I gene | G |
| ADP0012 | W6 16489 | red | Bush | Africa | 6.0 | 1411 | No I gene | G |
| ADP0018 | SODAN | red | Bush | Africa | 6.0 | 1372 | No I gene | G |
| ADP0042 | MKOKOLA | red | Vine | Africa | 6.0 | 1039 | No I gene | G |
| ADP0043 | BWANA SHAMBA | red | Vine | Africa | 6.0 | 1367 | No I gene | G |
| ADP0057 | KIJIVU | red | Vine | Africa | 6.0 | 1278 | No I gene | G |
| ADP0059 | Poto | purp. Spec | Ag.Climber | Africa | 6.0 | 522 | No I gene | G |
| ADP0065 | W6 16501 | red | Vine | Africa | 6.0 | 1017 | No I gene | G |
| ADP0100 | EG 21 | pur mot | Bush | Africa | 6.0 | 1472 | No I gene | G |
| ADP0106 | Zawadi | pur mot | Bush | Africa | 6.0 | 1117 | No I gene | G |
| ADP0205 | G 4494 | red mottled | Vine | CIAT core | 6.0 | 1211 | No I gene | G |
| ADP0458 | INIAP 483 | red mottled | Bush | Ecuador | 6.0 | 967 | No I gene | G |
| ADP0475 | Col.No 4670 , No2 | yellow | Vine | East Africa | 6.0 | 878 | No I gene | G |
| ADP0478 | G19310 | green | Bush | East Africa | 6.0 | 806 | ND | G |
| ADP0528 | LYAMUNGO 85 | red mottled | Bush | CIAT Africa | 6.0 | 1094 | No I gene | G |
| ADP0529 | LYAMUNGO 90 | d red mottled | Bush | CIAT Africa | 6.0 | 1239 | ND | G |
| ADP0553 | AND277 | red mottled | Bush | CIAT Africa | 6.0 | 1250 | No I gene | G |
| ADP0567 | ICA PALMAR | d. red mottled | Bush | CIAT Africa | 6.0 | 1589 | No I gene | G |
| ADP0571 | NUA45 | d red mottled | Bush | CIAT Africa | 6.0 | 1417 | No I gene | G |
| ADP0575 | SAB259 | CRAN | Bush | CIAT Africa | 6.0 | 1083 | No I gene | G |
| ADP0612 | ICA Quimbaya | red mottle | Bush | N American | 6.0 | 1128 | ND | G |
| ADP0635 | OAC Redstar | DRK | Bush | N American | 6.0 | 789 | I gene | G |
| ADP0637 | Isabella | LRK | Bush | N American | 6.0 | 1044 | I gene | G |
| ADP0643 | Cardinal | CRAN | Bush | N American | 6.0 | 1233 | I gene | G |
| ADP0646 | Myasi | Yellow | Bush | N American | 6.0 | 1528 | No I gene | G |
| ADP0687 | Pink Panther | LRK | Bush | N American | 6.0 | 1206 | ND | G |
| ADP0077 | NAMWANGA | purp spec | Ag.Climber | Africa | 6.0 | 617 | No I gene | T |
| ADP0447 | INIAP 414 | red mottled | Bush | Ecuador | 6.0 | 717 | No I gene | T |
| ADP0479 | IZ83 | yellow/ brown | Vine | East Africa | 6.0 | 494 | No I gene | T |
| ADP0521 | Cebo, Cela | manteca | Bush | Angola | 6.2 | 1217 | No I gene | T |
| ADP0005 | KABUKU | red | Bush | Africa | 6.3 | 1050 | No I gene | G |
| ADP0011 | KIBOROLONI | red | Bush | Africa | 6.3 | 982 | No I gene | G |
| ADP0032 | RH No. 21 | brn | Bush | Africa | 6.3 | 1261 | I gene | G |
| ADP0054 | W6 16447 | cran | Vine | Africa | 6.3 | 844 | No I gene | G |
| ADP0075 | MABUKU | brn | Vine | Africa | 6.3 | 1317 | No I gene | G |
| ADP0090 | KASUKANYWELE | zebra | Vine | Africa | 6.3 | 528 | No I gene | G |
| ADP0099 | Bwana Shamba | red | Bush | Africa | 6.3 | 1539 | No I gene | G |
| ADP0168 | KANYEBWA | pink cran | Vine | Africa | 6.3 | 1400 | ND | G |
| ADP0206 | G 4499 | white | Vine | CIAT core | 6.3 | 1083 | No I gene | G |
| ADP0208 | G 4644 | red mottled | Bush | CIAT core | 6.3 | 1178 | No I gene | G |
| ADP0345 | G 22147 | red | Bush | CIAT core | 6.3 | 744 | No I gene | G |
| ADP0353 | G 22455 | grey/green | Vine | CIAT core | 6.3 | 1094 | No I gene | G |
| ADP0470 | IZ10 | CRAN | Bush | East Africa | 6.3 | 1200 | No I gene | G |
| ADP0536 | CAL 96 | D. Red mottled | Bush | CIAT Africa | 6.3 | 1694 | No I gene | G |
| ADP0576 | SAB618 | Red mottled | Bush | CIAT Africa | 6.3 | 1800 | No I gene | G |
| ADP0584 | SAB659 | red mottled | Bush | CIAT Africa | 6.3 | 1272 | No I gene | G |
| ADP0603 | Wallace 773-V98 | LRK | Bush | N American | 6.3 | 1122 | I gene | G |
| ADP0641 | Capri | CRAN | Bush | N American | 6.3 | 1394 | I gene | G |
| ADP0663 | USCR-CBB-20 | CRAN | Bush | N American | 6.3 | 706 | I gene | G |
| ADP0678 | Hooter | CRAN | Bush | N American | 6.3 | 1700 | I gene | G |
| ADP0685 | Chianti | CRAN | Vine | N American | 6.3 | 1711 | I gene | G |
| ADP0725 | INTER LAB 02 | CRAN | Bush | E Africa | 6.3 | 1439 | I gene | G |
| ADP0048 | W6 16534 | red | Ag.Climber | Africa | 6.3 | 539 | No I gene | T |
| ADP0085 | KABLANKETI | purp spec | Ag.Climber | Africa | 6.3 | 956 | No I gene | T |
| ADP0098 | Selian 97 | red | Bush | Africa | 6.3 | 967 | No I gene | T |
| ADP0213 | G 5034 | grey | Vine | CIAT core | 6.3 | 478 | No I gene | T |
| ADP0468 | IZ114 | yellow | Bush | East Africa | 6.3 | 617 | No I gene | T |
| ADP0561 | DAB246 | red mottled | Bush | CIAT Africa | 6.3 | 1289 | No I gene | T |
| ADP0629 | H9659-27-10 | LRK | Vine | N American | 6.3 | 956 | I gene | T |
| ADP0655 | Fiero | DRK | Bush | N American | 6.3 | 1206 | I gene | T |
| ADP0006 | W6 16465 | red | Bush | Africa | 6.7 | 1211 | No I gene | G |
| ADP0017 | W6 16529 | red | Bush | Africa | 6.7 | 1356 | No I gene | G |
| ADP0047 | MSOLINI | brn | Vine | Africa | 6.7 | 1228 | No I gene | G |
| ADP0095 | CANADA | zebra | Vine | Africa | 6.7 | 1222 | No I gene | G |
| ADP0103 | Pesa | red | Bush | Africa | 6.7 | 806 | No I gene | G |
| ADP0232 | G 7930 | white | Bush | CIAT core | 6.7 | 1033 | No I gene | G |
| ADP0431 | Gurabo 5 | pink cran | Bush | Carribean | 6.7 | 1094 | No I gene | G |
| ADP0450 | INIAP 422 | white | Bush | Ecuador | 6.7 | 917 | ND | G |
| ADP0559 | DAB 555 |  | Bush | CIAT Africa | 6.7 | 683 | seg | G |
| ADP0560 | DAB230 | red mottled | Bush | CIAT Africa | 6.7 | 1267 | ND | G |
| ADP0588 | SAP 1 | d. red mottled | Bush | CIAT Africa | 6.7 | 1228 | No I gene | G |
| ADP0595 | MAYOCOBA | yellow | Bush | CIAT Africa | 6.7 | 1261 | ND | G |
| ADP0648 | Red Kloud | LRK | Bush | N American | 6.7 | 1556 | I gene | G |
| ADP0658 | Blush | LRK | Bush | N American | 6.7 | 1050 | I gene | G |
| ADP0023 | MSHORONYLONI | red | Bush | Africa | 6.7 | 1100 | No I gene | T |
| ADP0025 | RUHONDELA | purp spec | Bush | Africa | 6.7 | 811 | No I gene | T |
| ADP0050 | SALUNDE | gray | Vine | Africa | 6.7 | 683 | No I gene | T |
| ADP0053 | MAHARAGE MAKUBWA | red | Ag.Climber | Africa | 6.7 | 483 | No I gene | T |
| ADP0070 | Msafiri | red | Vine | Africa | 6.7 | 422 | No I gene | T |
| ADP0108 | Njano | yellow | Vine | Africa | 6.7 | 1194 | No I gene | T |
| ADP0112 | Uyole 96 | red | Vine | Africa | 6.7 | 1078 | No I gene | T |
| ADP0212 | G 4970 | tan | Bush | CIAT core | 6.7 | 628 | No I gene | T |
| ADP0224 | G 6239 | j. cattle; tan on white | Vine | CIAT core | 6.7 | 239 | No I gene | T |
| ADP0267 | G 12689 | purp cran | Bush | CIAT core | 6.7 | 933 | No I gene | T |
| ADP0367 | G 23086 | purp cran | Vine | CIAT core | 6.7 | 506 | No I gene | T |
| ADP0436 | JB-178 | red mottled | Bush | Carribean | 6.7 | 533 | No I gene | T |
| ADP0456 | INIAP 480 |  | Vine | Ecuador | 6.7 | 972 | ND | T |
| ADP0483 | No III | yellow | Vine | East Africa | 6.7 | 1156 | No I gene | T |
| ADP0534 | G 22501 | lt tan grey | Bush | CIAT Africa | 6.7 | 956 | No I gene | T |
| ADP0628 | H9659-27-7 | LRK | Vine | N American | 6.7 | 944 | I gene | T |
| ADP0630 | H9659-23-1 | LRK | Vine | N American | 6.7 | 1039 | I gene | T |
| ADP0633 | TARS-HT2 | LRK | Bush | N American | 6.7 | 1039 | No I gene | T |
| ADP0666 | USWK-6 | WK | Bush | N American | 6.7 | 1256 | NS | T |
| ADP0667 | VA-19 | LRK | Bush | N American | 6.7 | 972 | I gene | T |
| ADP0746 | PI661774 | dark pur | Vine | E Africa | 6.7 | 917 | ND | T |
| ADP0752 | Mahargi Red | red | Vine | E Africa | 6.7 | 1411 | ND | T |
| ADP0003 | KIDUNGU | red | Bush | Africa | 7.0 | 1050 | No I gene | G |
| ADP0016 | GOLOLI | red | Bush | Africa | 7.0 | 1139 | No I gene | G |
| ADP0101 | Witrood | cran | Bush | Africa | 7.0 | 428 | ND | G |
| ADP0449 | INIAP 420 | yellow | Bush | Ecuador | 7.0 | 283 | seg | G |
| ADP0525 | KAT B9 | red | Bush | CIAT Africa | 7.0 | 1411 | seg | G |
| ADP0601 | Camelot | red | Bush | N American | 7.0 | 994 | ND | G |
| ADP0624 | Dolly | CRAN | Bush | N American | 7.0 | 1450 | I gene | G |
| ADP0652 | Lisa | WK | Bush | N American | 7.0 | 550 | I gene | G |
| ADP0653 | USDK-CBB-15 | DRK | Bush | N American | 7.0 | 878 | I gene | G |
| ADP0742 | Canada | pur red | Vine | E Africa | 7.0 | 1239 | ND | G |
| ADP0745 | Super | dark pur | Vine | E Africa | 7.0 | 1217 | ND | G |
| ADP0747 | RH No 8 | red | Vine | E Africa | 7.0 | 694 | ND | G |
| ADP0028 | Sisi | yellow | Bush | Africa | 7.0 | 678 | No I gene | T |
| ADP0029 | RH No. 2 | red | Bush | Africa | 7.0 | 472 | No I gene | T |
| ADP0034 | KIJIVU | purp spec | Bush | Africa | 7.0 | 1089 | No I gene | T |
| ADP0046 | RH No. 4 | red | Ag.Climber | Africa | 7.0 | 844 | No I gene | T |
| ADP0392 | PI 309701 | cran | Vine | US core | 7.0 | 861 | No I gene | T |
| ADP0437 | PC-50 | red mottled | Bush | Carribean | 7.0 | 739 | No I gene | T |
| ADP0558 | DAB 528 | red | Bush | CIAT Africa | 7.0 | 928 | ND | T |
| ADP0682 | UI-686 | CRAN | Vine | N American | 7.0 | 978 | seg | T |
| ADP0683 | IJR | pink mottle | Vine | N American | 7.0 | 1533 | No I gene | T |
| ADP0684 | Majesty | DRK | Vine | N American | 7.0 | 1517 | I gene | T |
| ADP0015 | W6 16495 | red | Bush | Africa | 7.3 | 836 | No I gene | G |
| ADP0027 | Incomparable | brn | Bush | Africa | 7.3 | 983 | No I gene | G |
| ADP0031 | RH No. 11 | red | Bush | Africa | 7.3 | 1144 | No I gene | G |
| ADP0183 | G 994 | cream | Vine | CIAT core | 7.3 | 472 | ND | G |
| ADP0472 | IZ 102 | beige | Vine | East Africa | 7.3 | 533 | ND | G |
| ADP0583 | SAB650 | red mottled | Bush | CIAT Africa | 7.3 | 1533 | ND | G |
| ADP0014 | KIANGWE | yellow | Bush | Africa | 7.3 | 972 | No I gene | T |
| ADP0033 | KIJIVU | purp spec | Bush | Africa | 7.3 | 1050 | No I gene | T |
| ADP0044 | KIJIVU | purp spec | Vine | Africa | 7.3 | 1100 | No I gene | T |
| ADP0066 | NJANO | yellow | Vine | Africa | 7.3 | 644 | No I gene | T |
| ADP0102 | Jesca | pur mot | Bush | Africa | 7.3 | 1022 | No I gene | T |
| ADP0186 | G 1368 | red | Vine | CIAT core | 7.3 | 717 | NS | T |
| ADP0190 | G 1678 | purp spec | Vine | CIAT core | 7.3 | 256 | No I gene | T |
| ADP0203 | G 4472 |  | Vine | CIAT core | 7.3 | 478 | ND | T |
| ADP0354 | G 22502 | purp spec | Vine | CIAT core | 7.3 | 228 | No I gene | T |
| ADP0383 | PI 209486 | red mottled | Vine | US core | 7.3 | 578 | No I gene | T |
| ADP0413 | PI 319595 | cran | Ag.Climber | US core | 7.3 | 206 | No I gene | T |
| ADP0549 | RWR 10 | red | Vine | CIAT Africa | 7.3 | 1006 | No I gene | T |
| ADP0611 | Pompadour B | red mottle | Bush | N American | 7.3 | 556 | No I gene | T |
| ADP0620 | UCD0405 | red spec | Bush | N American | 7.3 | 506 | No I gene | T |
| ADP0640 | Beluga | WK | Bush | N American | 7.3 | 778 | I gene | T |
| ADP0674 | UCD 0704 | WK | Bush | N American | 7.3 | 828 | seg | T |
| ADP0686 | UCD 707 | Yellow | Bush | N American | 7.3 | 606 | I gene | T |
| ADP0743 | RH No 22 | d. red | Vine | E Africa | 7.3 | 928 | ND | T |
| ADP0021 | MBULAMTWE | yellow | Bush | Africa | 7.7 | 806 | No I gene | G |
| ADP0037 | W6 16488 | brn | Bush | Africa | 7.7 | 1072 | No I gene | G |
| ADP0303 | G 17913 | cream | Bush | CIAT core | 7.7 | 1008 | No I gene | G |
| ADP0464 | G39308 | Purple | Bush | East Africa | 7.7 | 706 | ND | G |
| ADP0524 | KAT B1 | green yellow | Bush | CIAT Africa | 7.7 | 822 | ND | G |
| ADP0657 | Kardinal | LRK | bush | N American | 7.7 | 422 | I gene | G |
| ADP0004 | KILOMBERO | yel brn zebra | Bush | Africa | 7.7 | 511 | No I gene | T |
| ADP0026 | Black Wonder | blk | Bush | Africa | 7.7 | 800 | No I gene | T |
| ADP0049 | W6 16546 | red | Vine | Africa | 7.7 | 667 | No I gene | T |
| ADP0078 | W6 16535 | maroon spec | Ag.Climber | Africa | 7.7 | 506 | No I gene | T |
| ADP0214 | G 5087 | black | Vine | CIAT core | 7.7 | 489 | No I gene | T |
| ADP0242 | G 9013 | cran | Bush | CIAT core | 7.7 | 839 | No I gene | T |
| ADP0247 | G 9975 | cran | Climber | CIAT core | 7.7 | 483 | No I gene | T |
| ADP0269 | G 13092 | white | Bush | CIAT core | 7.7 | 206 | No I gene | T |
| ADP0391 | PI 308894 | red kidney | Bush | US core | 7.7 | 439 | No I gene | T |
| ADP0460 | G19323 | Purple mottled | Bush | East Africa | 7.7 | 522 | No I gene | T |
| ADP0466 | Mwezi Moia | Purple speckled | Bush | East Africa | 7.7 | 1233 | No I gene | T |
| ADP0467 | Mweiumwe | Purple speckled | Vine | East Africa | 7.7 | 1111 | No I gene | T |
| ADP0469 | IZ63 | white | Bush | East Africa | 7.7 | 311 | seg | T |
| ADP0480 | Kikaa | Purple mottled | Bush | East Africa | 7.7 | 639 | No I gene | T |
| ADP0596 | G16104E | pur/black | Bush | CIAT Africa | 7.7 | 267 | No I gene | T |
| ADP0610 | G-122 | CRAN | Bush | N American | 7.7 | 628 | No I gene | T |
| ADP0613 | 02-385-14 | LRK | Bush | N American | 7.7 | 561 | I gene | T |
| ADP0642 | Taylor Hort | CRAN | Bush | N American | 7.7 | 550 | No I gene | T |
| ADP0656 | Royal Red | DRK | Bush | N American | 7.7 | 556 | I gene | T |
| ADP0664 | Silver Cloud | WK | Bush | N American | 7.7 | 572 | I gene | T |
| ADP0668 | Cran-09 | CRAN | Bush | N American | 7.7 | 694 | No I gene | T |
| ADP0670 | AC Calmont | DRK | Bush | N American | 7.7 | 861 | I gene | T |
| ADP0750 | Kwasa Mangube | dark red | Bush | E Africa | 7.7 | 678 | ND | T |
| ADP0067 | NJANO | yellow | Ag.Climber | Africa | 8.0 | 489 | No I gene | G |
| ADP0662 | USCR-9 | CRAN | Bush | N American | 8.0 | 389 | NS | G |
| ADP0010 | CANADA | red | Bush | Africa | 8.0 | 667 | No I gene | T |
| ADP0013 | KIBUMBULA | red | Bush | Africa | 8.0 | 467 | No I gene | T |
| ADP0038 | Moono | red | Bush | Africa | 8.0 | 633 | No I gene | T |
| ADP0180 | G 433 | cran | Bush | CIAT core | 8.0 | 333 | ND | T |
| ADP0220 | G 5625 | red | Vine | CIAT core | 8.0 | 283 | No I gene | T |
| ADP0271 | G 13167 | white | Bush | CIAT core | 8.0 | 239 | ND | T |
| ADP0277 | G 13778 | purp mottled | Bush | CIAT core | 8.0 | 400 | No I gene | T |
| ADP0280 | G 14440 | white | Vine | CIAT core | 8.0 | 250 | No I gene | T |
| ADP0288 | G 16110A | Red | Ag.Climber | CIAT core | 8.0 | 267 | No I gene | T |
| ADP0519 | Katarina, Cela | CRAN | Bush | Angola | 8.0 | 706 | seg | T |
| ADP0543 | G 16157 | LRK | Bush | CIAT Africa | 8.0 | 392 | No I gene | T |
| ADP0546 | RED CANADIAN WONDER | dark red-pur | Vine | CIAT Africa | 8.0 | 511 | No I gene | T |
| ADP0557 | COS16 | CRAN | Bush | CIAT Africa | 8.0 | 875 | I gene | T |
| ADP0569 | MDRK | dark red | Bush | CIAT Africa | 8.0 | 661 | ND | T |
| ADP0577 | SAB620 |  | Bush | CIAT Africa | 8.0 | 178 | ND | T |
| ADP0579 | SAB623 |  | Bush | CIAT Africa | 8.0 | 422 | ND | T |
| ADP0587 | SAB712 | white | Bush | CIAT Africa | 8.0 | 200 | No I gene | T |
| ADP0591 | VELAZCO LARGO | LRK | Bush | CIAT Africa | 8.0 | 556 | No I gene | T |
| ADP0604 | 1062-V98 | LRK | Bush | N American | 8.0 | 200 | I gene | T |
| ADP0638 | Red Hawk | DRK | Bush | N American | 8.0 | 722 | I gene | T |
| ADP0645 | Lassen | WK | Bush | N American | 8.0 | 411 | No I gene | T |
| ADP0650 | K-42 | LRK | Bush | N American | 8.0 | 506 | I gene | T |
| ADP0672 | CDRK | DRK | Bush | N American | 8.0 | 567 | No I gene | T |
| ADP0675 | UCD 0801 | CRAN | Bush | N American | 8.0 | 283 | I gene | T |
| ADP0677 | Etna | CRAN | Bush | N American | 8.0 | 628 | I gene | T |
| ADP0679 | Red Rover | DRK | Bush | N American | 8.0 | 589 | I gene | T |
| ADP0376 | PI 189408 | red | Bush | US core | 8.3 | 261 | No I gene | T |
| ADP0390 | PI 307808 | red | Bush | US core | 8.3 | 222 | No I gene | T |
| ADP0417 | PI 451906 | red kidney | Bush | US core | 8.3 | 211 | No I gene | T |
| ADP0459 | G19323 | CRAN | Bush | East Africa | 8.3 | 239 | No I gene | T |
| ADP0607 | NY 105 | LRK | Bush | N American | 8.3 | 244 | I gene | T |
| ADP0609 | K-407 | DRK | Bush | N American | 8.3 | 344 | I gene | T |
| ADP0622 | UCD 0701 | Jacobs cattle | Bush | N American | 8.3 | 539 | I gene | T |
| ADP0634 | UC Red Kidney | LRK | Bush | N American | 8.3 | 294 | No I gene | T |
| ADP0659 | USLK-1 | LRK | Bush | N American | 8.3 | 456 | NS | T |
| ADP0673 | UC Nichols | DRK | Bush | N American | 8.3 | 361 | seg | T |
| ADP0676 | CELRK | DRK | Bush | N American | 8.3 | 400 | I gene | T |
| ADP0207 | G 4564 | j. cattle | Bush | CIAT core | 8.7 | 456 | No I gene | T |
| ADP0606 | NY 104 | LRK | Bush | N American | 8.7 | 194 | I gene | T |
| ADP0310 | G 18356 | cran | Bush | CIAT core | 9.0 | 194 | No I gene | T |
| ADP0349 | G 22357 | red mottled | Bush | CIAT core | 9.0 | 278 | ND | T |
| ADP0598 | Charlevoix | DRK | Bush | N American | 9.0 | 256 | ND | T |
| ADP0623 | Drake | DRK | Bush | N American | 9.0 | 306 | I gene | T |

**Table S6.** Halo blight host differentials rated 1 to 9 for reaction to inoculation with *Pseudomonas syringae* pv. *phaseolicola* Race 6 in the field in Potchefstroom, South Africa, in 2014.

| **Host differential** |  |  |  |  |  |
| --- | --- | --- | --- | --- | --- |
|  | **Rep 1** | **Rep 2** | **Rep 3** | **Mean** | ***R* genes** |
| Guatemala 196-B | 1 | 1 | 1 | 1.0 | *Pse-1, -3, -4* |
| A43 (ZAA 12) | 4 | 3 | 3 | 3.3 | *Pse-2, -3, -4* |
| A53 (ZAA 55) | 5 | 6 | 5 | 5.3 | *Pse-3, -4* |
| Red Mexican UI 3 | 5 | 3 | 5 | 4.3 | *Pse-1, -4* |
| A52 (ZAA 54) | 5 | 5 | 7 | 5.7 | *Pse-4* |
| Tendergreen | 6 | 7 | 7 | 6.7 | *Pse-3* |
| Canadian Wonder | 9 | 9 | 9 | 9.0 | None |

**Table S7.** Marker–trait associations for QTL HB5.1 for disease score and yield in the *Phaseolus vulgaris* Andean Diversity Panel under severe halo blight pressure following field inoculation with *Pseudomonas syringae* pv. *phaseolicola* Race 6.

| Trait | SNP | Chromosome | Position | Probability | −log_10_(*p*) | MAF |
| --- | --- | --- | --- | --- | --- | --- |
| Disease score | S5_38725023 | 5 | 38,725,023 | 1.53E−07 | 6.817 | 0.417 |
| Disease score | S5_38885617 | 5 | 38,885,617 | 2.64E−07 | 6.579 | 0.414 |
| Disease score | S5_38866881 | 5 | 38,866,881 | 2.64E−07 | 6.579 | 0.417 |
| Yield | S5_38725023 | 5 | 38,725,023 | 2.57E−07 | 6.590 | 0.417 |
| Yield | S5_38866881 | 5 | 38,866,881 | 6.33E−07 | 6.199 | 0.417 |

Associations were assessed with the mixed linear model (MLM) using the Genome Association and Prediction Integrated Tool (GAPIT) while controlling for population structure. MAF: minor allele frequency.

| **Table S8.** HB5.1 SNP haplotypes. | | |  | SNP genotypes for HB5.1 QTL on Pv05 | | |  | Haplotype compared with Rojo ADP_0096 | | | |
| --- | --- | --- | --- | --- | --- | --- | --- | --- | --- | --- | --- |
| ADP  # |  | HBB Mean Score to Race 6 | S5_38725023 | S5_38866881 | S5_38885617 | S5_38928670 |  | S5_38725023 | S5_38866881 | S5_38885617 | S5_38928670 |
|  |  |  | G/T | T/G | A/T | C/T |  | G/T | T/G | A/T | C/T |
| ADP0121 | Kranskop HR-1 | 1.7 | G | T | A | C |  | A | A | A | A |
| ADP0118 | Werna | 2 | G | T | A | C |  | A | A | A | A |
| ADP0797 | cranberry | 2 | G | T | A | C |  | A | A | A | A |
| ADP0716 | CRAN | 2.3 | G | T | A | C |  | A | A | A | A |
| ADP0084 | KABLANKETI NDEFU | 2.7 | G | T | * | C |  | A | A | * | A |
| ADP0125 | CHEUPE | 2.7 | G | T | A | C |  | A | A | A | A |
| ADP0255 | G 10994 | 2.7 | G | T | * | C |  | A | A | * | A |
| ADP0276 | G 13654 | 2.7 | G | T | A | C |  | A | A | A | A |
| ADP0454 | INIAP 429 | 2.7 | G | T | A | C |  | A | A | A | A |
| ADP0526 | CAL 143 | 2.7 | T | G | T | T |  | B | B | B | B |
| ADP0723 | CRAN | 2.7 | G | T | A | C |  | A | A | A | A |
| ADP0734 | RD MOTT | 2.7 | * | * | * | C |  | * | * | * | A |
| ADP0428 | Colorado del Pais | 2.8 | G | T | A | C |  | A | A | A | A |
| ADP0737 | RD MOTT | 2.8 | G | T | A | C |  | A | A | A | A |
| ADP0080 | KABLANKETI | 3 | G | T | A | C |  | A | A | A | A |
| ADP0114 | OPS-RS1 | 3 | G | T | A | C |  | A | A | A | A |
| ADP0120 | Tygerberg | 3 | G | T | A | C |  | A | A | A | A |
| ADP0432 | PR0637-134 | 3 | G | T | A | C |  | A | A | A | A |
| ADP0566 | G5686 | 3 | G | T | * | C |  | A | A | * | A |
| ADP0592 | AND 1005 | 3 | G | T | A | C |  | A | A | A | A |
| ADP0731 | RD MOTT | 3 | T | G | T | T |  | B | B | B | B |
| ADP0740 | RD MOTT | 3 | T | G | T | T |  | B | B | B | B |
| ADP0798 | cranberry | 3 | G | T | A | C |  | A | A | A | A |
| ADP0122 | Kranskop | 3.3 | G | T | A | C |  | A | A | A | A |
| ADP0123 | Jenny | 3.3 | G | T | A | C |  | A | A | A | A |
| ADP0379 | PI 203934 | 3.3 | G | T | * | C |  | A | A | * | A |
| ADP0508 | Calembe | 3.3 | G | T | A | C |  | A | A | A | A |
| ADP0537 | AFR 619 | 3.3 | G | T | A | C |  | A | A | A | A |
| ADP0572 | NUA56 | 3.3 | G | T | A | C |  | A | A | A | A |
| ADP0728 | LRK | 3.3 | G | T | A | C |  | A | A | A | A |
| ADP0732 | RD MOTT | 3.3 | T | G | * | T |  | B | B | * | B |
| ADP0736 | RD MOTT | 3.3 | G | T | A | C |  | A | A | A | A |
| ADP0739 | TAN/PUR | 3.3 | G | T | A | C |  | A | A | A | A |
| ADP0730 | PUR MOTT | 3.5 | T | G | T | T |  | B | B | B | B |
| ADP0082 | KABLANKETI | 3.7 | G | T | A | C |  | A | A | A | A |
| ADP0433 | PR9745-232 | 3.7 | G | T | * | C |  | A | A | * | A |
| ADP0541 | CIM 9314-36 | 3.7 | G | T | A | C |  | A | A | A | A |
| ADP0555 | BRB191 | 3.7 | T | G | T | T |  | B | B | B | B |
| ADP0597 | G23829 | 3.7 | G | T | A | C |  | A | A | A | A |
| ADP0721 | DRK | 3.7 | G | T | A | C |  | A | A | A | A |
| ADP0735 | RD MOTT | 3.7 | G | T | A | C |  | A | A | A | A |
| ADP0654 | USDK-4 | 3.8 | T | G | * | T |  | B | B | * | B |
| ADP0008 | Nyayo | 4 | * | T | A | C |  | * | A | A | A |
| ADP0030 | RH No. 6 | 4 | G | T | A | C |  | A | A | A | A |
| ADP0055 | KABUKU | 4 | G | T | A | C |  | A | A | A | A |
| ADP0109 | Kablanketi | 4 | G | T | A | C |  | A | A | A | A |
| ADP0113 | OPS-RS4 | 4 | G | T | A | C |  | A | A | A | A |
| ADP0117 | A483 | 4 | T | G | T | T |  | B | B | B | B |
| ADP0119 | A193 | 4 | G | T | A | C |  | A | A | A | A |
| ADP0192 | G 2377 | 4 | * | T | A | * |  | * | A | A | * |
| ADP0272 | G 13336 | 4 | G | T | A | C |  | A | A | A | A |
| ADP0395 | PI 310511 | 4 | G | T | A | C |  | A | A | A | A |
| ADP0430 | PR1013-3 | 4 | G | T | A | C |  | A | A | A | A |
| ADP0442 | Larga Comercial | 4 | G | T | A | C |  | A | A | A | A |
| ADP0531 | AND 620 | 4 | G | T | A | C |  | A | A | A | A |
| ADP0574 | RADICAL CERINZA | 4 | G | T | A | C |  | A | A | A | A |
| ADP0632 | TARS HT 1 | 4 | G | T | A | C |  | A | A | A | A |
| ADP0738 | RD MOTT | 4 | T | G | T | T |  | B | B | B | B |
| ADP0510 | Ohlio de perdiz | 4.2 | T | G | T | T |  | B | B | B | B |
| ADP0061 | Maulasi | 4.3 | G | T | A | C |  | A | A | A | A |
| ADP0081 | KABLANKETI | 4.3 | G | T | A | C |  | A | A | A | A |
| ADP0089 | KABLANKETI | 4.3 | G | T | A | C |  | A | A | A | A |
| ADP0211 | G 4780 | 4.3 | G | T | A | C |  | A | A | A | A |
| ADP0520 | Chumbo, Cela | 4.3 | G | * | * | C |  | A | * | * | A |
| ADP0551 | AFR 612 | 4.3 | G | T | A | C |  | A | A | A | A |
| ADP0590 | SEQ11 | 4.3 | G | T | * | C |  | A | A | * | A |
| ADP0647 | Red Kanner | 4.3 | G | T | A | C |  | A | A | A | A |
| ADP0719 | PUR MOTT | 4.3 | G | T | A | C |  | A | A | A | A |
| ADP0720 | CRAN | 4.3 | G | T | A | C |  | A | A | A | A |
| ADP0794 | cranberry | 4.3 | G | T | A | C |  | A | A | A | A |
| ADP0796 | cranberry | 4.3 | G | T | A | C |  | A | A | A | A |
| ADP0009 | Maalasa | 4.7 | G | T | A | C |  | A | A | A | A |
| ADP0035 | Kokola | 4.7 | G | T | A | C |  | A | A | A | A |
| ADP0036 | Lyamungu 85 | 4.7 | G | T | A | C |  | A | A | A | A |
| ADP0056 | SOYA | 4.7 | G | T | * | C |  | A | A | * | A |
| ADP0088 | KABLANKETI | 4.7 | G | T | A | C |  | A | A | A | A |
| ADP0115 | Bonus | 4.7 | G | T | A | C |  | A | A | A | A |
| ADP0127 | SELIAN 06 | 4.7 | * | G | * | * |  | * | B | * | * |
| ADP0427 | Badillo | 4.7 | G | T | A | C |  | A | A | A | A |
| ADP0462 | PI527540-B | 4.7 | G | T | A | C |  | A | A | A | A |
| ADP0527 | POA 2 | 4.7 | T | G | T | T |  | B | B | B | B |
| ADP0545 | PAN 127 | 4.7 | G | * | A | C |  | A | * | A | A |
| ADP0631 | OAC Inferno | 4.7 | G | T | A | C |  | A | A | A | A |
| ADP0660 | Krimson | 4.7 | T | G | * | T |  | B | B | * | B |
| ADP0665 | USWK-CBB-17 | 4.7 | T | G | * | T |  | B | B | * | B |
| ADP0722 | CRAN | 4.7 | G | T | A | C |  | A | A | A | A |
| ADP0726 | CRAN | 4.7 | G | T | A | C |  | A | A | A | A |
| ADP0748 | pink mottled | 4.7 | T | G | T | T |  | B | B | B | B |
| ADP0751 | red mottled | 4.7 | G | T | A | C |  | A | A | A | A |
| ADP0001 | ROZI KOKO | 5 | G | T | A | C |  | A | A | A | A |
| ADP0063 | Soya | 5 | G | T | A | C |  | A | A | A | A |
| ADP0076 | KABLANKETI | 5 | G | T | A | C |  | A | A | A | A |
| ADP0086 | NYAMHONGA MWEKUNDU | 5 | T | G | T | T |  | B | B | B | B |
| ADP0110 | SUG-131 | 5 | G | T | A | C |  | A | A | A | A |
| ADP0166 | NABE 4 | 5 | T | G | T | T |  | B | B | B | B |
| ADP0225 | G 6415 | 5 | G | T | A | C |  | A | A | A | A |
| ADP0337 | G 21303 | 5 | G | T | A | C |  | A | A | A | A |
| ADP0434 | PR0737-1 | 5 | T | G | T | T |  | B | B | B | B |
| ADP0455 | INIAP 430 | 5 | G | T | A | C |  | A | A | A | A |
| ADP0465 | PI321094-D | 5 | G | T | A | C |  | A | A | A | A |
| ADP0513 | Canario | 5 | G | T | A | C |  | A | A | A | A |
| ADP0530 | SELIAN 94 | 5 | T | G | T | T |  | B | B | B | B |
| ADP0544 | PVA 773 | 5 | T | G | T | T |  | B | B | B | B |
| ADP0615 | Litekid | 5 | G | T | A | C |  | A | A | A | A |
| ADP0621 | Jalo EEP558 | 5 | G | T | A | C |  | A | A | A | A |
| ADP0636 | Montcalm | 5 | G | T | A | C |  | A | A | A | A |
| ADP0649 | Kamiakin | 5 | G | T | A | C |  | A | A | A | A |
| ADP0717 | CRAN | 5 | T | G | T | T |  | B | B | B | B |
| ADP0724 | DRK | 5 | T | G | T | T |  | B | B | B | B |
| ADP0744 | pink mottled | 5 | G | T | A | C |  | A | A | A | A |
| ADP0041 | MRONDO | 5.3 | G | T | A | C |  | A | A | A | A |
| ADP0051 | RH No. 3 | 5.3 | G | T | A | C |  | A | A | A | A |
| ADP0052 | RH No. 9 | 5.3 | G | T | A | C |  | A | A | A | A |
| ADP0060 | CANADA | 5.3 | G | T | A | C |  | A | A | A | A |
| ADP0062 | MAULASI | 5.3 | G | T | A | C |  | A | A | A | A |
| ADP0087 | KABLANKETI | 5.3 | G | T | A | C |  | A | A | A | A |
| ADP0096 | Rojo | 5.3 | G | T | A | C |  | A | A | A | A |
| ADP0279 | G 14423 | 5.3 | T | G | T | T |  | B | B | B | B |
| ADP0346 | G 22246 | 5.3 | G | T | A | C |  | A | A | A | A |
| ADP0368 | G 23093 | 5.3 | G | T | A | C |  | A | A | A | A |
| ADP0435 | RM-05-07 | 5.3 | G | T | A | C |  | A | A | A | A |
| ADP0438 | 46-1 | 5.3 | G | * | * | C |  | A | * | * | A |
| ADP0453 | INIAP 428 | 5.3 | T | G | T | T |  | B | B | B | B |
| ADP0457 | INIAP 481 | 5.3 | G | T | A | C |  | A | A | A | A |
| ADP0481 | PI449428 | 5.3 | G | T | A | C |  | A | A | A | A |
| ADP0523 | Canario, Cela | 5.3 | G | T | A | C |  | A | A | A | A |
| ADP0532 | A 197 | 5.3 | G | T | A | C |  | A | A | A | A |
| ADP0564 | G 5164 | 5.3 | T | G | T | T |  | B | B | B | B |
| ADP0580 | SAB626 | 5.3 | G | T | A | C |  | A | A | A | A |
| ADP0586 | SAB691 | 5.3 | G | T | A | C |  | A | A | A | A |
| ADP0626 | Badillo | 5.3 | G | T | A | C |  | A | A | A | A |
| ADP0639 | Chinook 2000 | 5.3 | G | T | A | C |  | A | A | A | A |
| ADP0651 | K-59 | 5.3 | G | T | A | C |  | A | A | A | A |
| ADP0729 | STRIP PUR | 5.3 | T | G | T | T |  | B | B | B | B |
| ADP0741 | brown | 5.3 | G | T | A | C |  | A | A | A | A |
| ADP0753 | black and white | 5.3 | G | T | A | C |  | A | A | A | A |
| ADP0795 | cranberry | 5.3 | G | T | A | C |  | A | A | A | A |
| ADP0022 | KISAPURI | 5.7 | G | T | A | C |  | A | A | A | A |
| ADP0045 | RH No. 12 | 5.7 | G | T | A | C |  | A | A | A | A |
| ADP0058 | CANADA | 5.7 | T | G | T | T |  | B | B | B | B |
| ADP0064 | W6 16500 | 5.7 | G | T | A | C |  | A | A | A | A |
| ADP0069 | SOYA | 5.7 | G | T | A | C |  | A | A | A | A |
| ADP0074 | KABLANKETI | 5.7 | G | T | A | C |  | A | A | A | A |
| ADP0083 | W6 16547 | 5.7 | T | G | T | T |  | B | B | B | B |
| ADP0092 | MORO | 5.7 | G | T | A | C |  | A | A | A | A |
| ADP0105 | Sewani 97 | 5.7 | T | G | T | T |  | B | B | B | B |
| ADP0471 | PI527537-C | 5.7 | T | G | T | T |  | B | B | B | B |
| ADP0476 | Heirloom | 5.7 | G | T | A | C |  | A | A | A | A |
| ADP0482 | PI209802 | 5.7 | G | T | A | C |  | A | A | A | A |
| ADP0516 | Mantega, Kibala | 5.7 | G | T | A | C |  | A | A | A | A |
| ADP0518 | Mantega blanca, Kibala | 5.7 | G | T | A | C |  | A | A | A | A |
| ADP0535 | ARA 4 | 5.7 | G | T | A | C |  | A | A | A | A |
| ADP0538 | RWR 221 | 5.7 | G | T | A | C |  | A | A | A | A |
| ADP0540 | AFR 708 | 5.7 | G | T | A | C |  | A | A | A | A |
| ADP0554 | AND279 | 5.7 | G | T | A | C |  | A | A | A | A |
| ADP0556 | BRB194 | 5.7 | G | T | A | C |  | A | A | A | A |
| ADP0562 | DAB387 | 5.7 | G | T | A | C |  | A | A | A | A |
| ADP0570 | NATALSUGAR | 5.7 | T | G | T | T |  | B | B | B | B |
| ADP0581 | SAB629 | 5.7 | G | T | A | C |  | A | A | A | A |
| ADP0582 | SAB630 | 5.7 | G | T | A | C |  | A | A | A | A |
| ADP0585 | SAB686 | 5.7 | G | T | A | C |  | A | A | A | A |
| ADP0599 | Isles | 5.7 | G | T | A | C |  | A | A | A | A |
| ADP0605 | 1132-V96 | 5.7 | G | T | A | C |  | A | A | A | A |
| ADP0614 | ND061106 | 5.7 | G | T | A | C |  | A | A | A | A |
| ADP0644 | Fox Fire | 5.7 | G | T | A | C |  | A | A | A | A |
| ADP0680 | Clouseau | 5.7 | G | T | A | C |  | A | A | A | A |
| ADP0727 | TAN | 5.7 | G | T | A | C |  | A | A | A | A |
| ADP0094 | LUSHALA | 5.8 | T | G | T | T |  | B | B | B | B |
| ADP0007 | BUKOBA | 6 | G | T | * | C |  | A | A | * | A |
| ADP0012 | W6 16489 | 6 | G | T | A | C |  | A | A | A | A |
| ADP0018 | SODAN | 6 | G | T | A | C |  | A | A | A | A |
| ADP0042 | MKOKOLA | 6 | G | T | A | C |  | A | A | A | A |
| ADP0043 | BWANA SHAMBA | 6 | G | T | A | C |  | A | A | A | A |
| ADP0057 | KIJIVU | 6 | G | T | A | C |  | A | A | A | A |
| ADP0059 | Poto | 6 | G | T | A | C |  | A | A | A | A |
| ADP0065 | W6 16501 | 6 | G | T | A | C |  | A | A | A | A |
| ADP0077 | NAMWANGA | 6 | T | G | T | T |  | B | B | B | B |
| ADP0100 | EG 21 | 6 | G | T | A | C |  | A | A | A | A |
| ADP0106 | Zawadi | 6 | G | T | A | C |  | A | A | A | A |
| ADP0205 | G 4494 | 6 | G | T | A | C |  | A | A | A | A |
| ADP0447 | INIAP 414 | 6 | T | G | T | T |  | B | B | B | B |
| ADP0458 | INIAP 483 | 6 | G | T | A | C |  | A | A | A | A |
| ADP0475 | PI319706 | 6 | G | T | A | C |  | A | A | A | A |
| ADP0478 | PI353536 | 6 | G | T | A | C |  | A | A | A | A |
| ADP0479 | PI527530 | 6 | T | G | T | T |  | B | B | B | B |
| ADP0528 | LYAMUNGO 85 | 6 | G | T | A | C |  | A | A | A | A |
| ADP0529 | LYAMUNGO 90 | 6 | G | T | A | C |  | A | A | A | A |
| ADP0553 | AND277 | 6 | G | T | A | C |  | A | A | A | A |
| ADP0567 | G 4523 | 6 | G | T | A | C |  | A | A | A | A |
| ADP0571 | NUA45 | 6 | G | T | A | C |  | A | A | A | A |
| ADP0575 | SAB259 | 6 | G | T | A | C |  | A | A | A | A |
| ADP0612 | ICA Quimbaya | 6 | G | T | A | C |  | A | A | A | A |
| ADP0635 | OAC Redstar | 6 | G | T | A | C |  | A | A | A | A |
| ADP0637 | Isabella | 6 | G | T | A | C |  | A | A | A | A |
| ADP0643 | Cardinal | 6 | G | T | A | C |  | A | A | A | A |
| ADP0646 | Myasi | 6 | G | T | A | C |  | A | A | A | A |
| ADP0687 | Pink Panther | 6 | G | T | A | C |  | A | A | A | A |
| ADP0521 | Cebo, Cela | 6.2 | T | G | T | T |  | B | B | B | B |
| ADP0005 | KABUKU | 6.3 | G | T | A | C |  | A | A | A | A |
| ADP0011 | KIBOROLONI | 6.3 | G | T | A | C |  | A | A | A | A |
| ADP0032 | RH No. 21 | 6.3 | G | T | A | C |  | A | A | A | A |
| ADP0048 | W6 16534 | 6.3 | T | G | T | T |  | B | B | B | B |
| ADP0054 | W6 16447 | 6.3 | G | T | A | C |  | A | A | A | A |
| ADP0075 | MABUKU | 6.3 | G | T | A | C |  | A | A | A | A |
| ADP0085 | KABLANKETI | 6.3 | T | G | * | C |  | B | B | * | A |
| ADP0090 | KASUKANYWELE | 6.3 | G | T | A | C |  | A | A | A | A |
| ADP0098 | Selian 97 | 6.3 | T | G | T | T |  | B | B | B | B |
| ADP0099 | Bwana Shamba | 6.3 | G | T | A | C |  | A | A | A | A |
| ADP0168 | KANYEBWA | 6.3 | G | T | A | C |  | A | A | A | A |
| ADP0206 | G 4499 | 6.3 | G | T | A | C |  | A | A | A | A |
| ADP0208 | G 4644 | 6.3 | G | T | A | C |  | A | A | A | A |
| ADP0213 | G 5034 | 6.3 | T | T | A | C |  | B | A | A | A |
| ADP0345 | G 22147 | 6.3 | G | T | A | C |  | A | A | A | A |
| ADP0353 | G 22455 | 6.3 | G | T | A | C |  | A | A | A | A |
| ADP0468 | PI527538 | 6.3 | T | G | T | T |  | B | B | B | B |
| ADP0470 | PI527508 | 6.3 | G | T | A | C |  | A | A | A | A |
| ADP0536 | CAL 96 | 6.3 | G | T | A | C |  | A | A | A | A |
| ADP0561 | DAB246 | 6.3 | T | G | T | T |  | B | B | B | B |
| ADP0576 | SAB618 | 6.3 | G | T | * | C |  | A | A | * | A |
| ADP0584 | SAB659 | 6.3 | G | T | * | C |  | A | A | * | A |
| ADP0603 | Wallace 773-V98 | 6.3 | G | T | * | C |  | A | A | * | A |
| ADP0629 | H9659-27-10 | 6.3 | T | G | T | T |  | B | B | B | B |
| ADP0641 | Capri | 6.3 | G | T | A | C |  | A | A | A | A |
| ADP0655 | Fiero | 6.3 | T | G | * | T |  | B | B | * | B |
| ADP0663 | USCR-CBB-20 | 6.3 | G | T | A | C |  | A | A | A | A |
| ADP0678 | Hooter | 6.3 | G | T | A | C |  | A | A | A | A |
| ADP0685 | Chianti | 6.3 | G | T | * | C |  | A | A | * | A |
| ADP0725 | CRAN | 6.3 | G | T | A | C |  | A | A | A | A |
| ADP0006 | W6 16465 | 6.7 | G | T | A | C |  | A | A | A | A |
| ADP0017 | W6 16529 | 6.7 | G | T | A | C |  | A | A | A | A |
| ADP0023 | MSHORONYLONI | 6.7 | T | G | T | T |  | B | B | B | B |
| ADP0025 | RUHONDELA | 6.7 | T | G | T | T |  | B | B | B | B |
| ADP0047 | MSOLINI | 6.7 | G | T | A | C |  | A | A | A | A |
| ADP0050 | SALUNDE | 6.7 | T | G | T | T |  | B | B | B | B |
| ADP0053 | MAHARAGE MAKUBWA | 6.7 | T | G | T | T |  | B | B | B | B |
| ADP0070 | Msafiri | 6.7 | T | G | T | T |  | B | B | B | B |
| ADP0095 | CANADA | 6.7 | G | T | A | C |  | A | A | A | A |
| ADP0103 | Pesa | 6.7 | G | T | A | C |  | A | A | A | A |
| ADP0108 | Njano | 6.7 | T | G | T | T |  | B | B | B | B |
| ADP0112 | Uyole 96 | 6.7 | T | G | T | T |  | B | B | B | B |
| ADP0212 | G 4970 | 6.7 | T | T | A | C |  | B | A | A | A |
| ADP0224 | G 6239 | 6.7 | T | G | T | T |  | B | B | B | B |
| ADP0232 | G 7930 | 6.7 | G | T | * | C |  | A | A | * | A |
| ADP0267 | G 12689 | 6.7 | T | G | T | T |  | B | B | B | B |
| ADP0367 | G 23086 | 6.7 | T | G | T | T |  | B | B | B | B |
| ADP0431 | Gurabo 5 | 6.7 | G | T | A | C |  | A | A | A | A |
| ADP0436 | JB-178 | 6.7 | T | G | * | T |  | B | B | * | B |
| ADP0450 | INIAP 422 | 6.7 | G | T | * | C |  | A | A | * | A |
| ADP0456 | INIAP 480 | 6.7 | T | G | T | T |  | B | B | B | B |
| ADP0483 | PI209815 | 6.7 | T | G | T | T |  | B | B | B | B |
| ADP0534 | G 22501 | 6.7 | T | G | T | T |  | B | B | B | B |
| ADP0559 | DAB 555 | 6.7 | G | G | T | T |  | A | B | B | B |
| ADP0560 | DAB230 | 6.7 | G | T | A | C |  | A | A | A | A |
| ADP0588 | SAP 1 | 6.7 | G | T | A | C |  | A | A | A | A |
| ADP0595 | G13094 | 6.7 | G | T | A | C |  | A | A | A | A |
| ADP0628 | H9659-27-7 | 6.7 | T | G | T | T |  | B | B | B | B |
| ADP0630 | H9659-23-1 | 6.7 | T | G | T | T |  | B | B | B | B |
| ADP0633 | TARS-HT2 | 6.7 | T | G | T | T |  | B | B | B | B |
| ADP0648 | Red Kloud | 6.7 | G | T | A | C |  | A | A | A | A |
| ADP0658 | Blush | 6.7 | G | T | A | C |  | A | A | A | A |
| ADP0666 | USWK-6 | 6.7 | T | G | * | T |  | B | B | * | B |
| ADP0667 | VA-19 | 6.7 | T | G | T | T |  | B | B | B | B |
| ADP0746 | dark pur | 6.7 | T | G | T | T |  | B | B | B | B |
| ADP0752 | red | 6.7 | T | G | T | T |  | B | B | B | B |
| ADP0003 | KIDUNGU | 7 | G | T | A | C |  | A | A | A | A |
| ADP0016 | GOLOLI | 7 | G | T | A | C |  | A | A | A | A |
| ADP0028 | Sisi | 7 | T | G | * | T |  | B | B | * | B |
| ADP0029 | RH No. 2 | 7 | T | G | T | T |  | B | B | B | B |
| ADP0034 | KIJIVU | 7 | T | G | T | T |  | B | B | B | B |
| ADP0046 | RH No. 4 | 7 | T | G | T | T |  | B | B | B | B |
| ADP0101 | Witrood | 7 | G | T | A | C |  | A | A | A | A |
| ADP0392 | PI 309701 | 7 | T | G | T | T |  | B | B | B | B |
| ADP0437 | PC-50 | 7 | T | G | T | T |  | B | B | B | B |
| ADP0449 | INIAP 420 | 7 | G | T | A | C |  | A | A | A | A |
| ADP0525 | KAT B9 | 7 | G | T | A | C |  | A | A | A | A |
| ADP0558 | DAB 528 | 7 | T | G | T | T |  | B | B | B | B |
| ADP0601 | Camelot | 7 | G | T | A | C |  | A | A | A | A |
| ADP0624 | Dolly | 7 | G | T | A | C |  | A | A | A | A |
| ADP0652 | Lisa | 7 | G | T | A | C |  | A | A | A | A |
| ADP0653 | USDK-CBB-15 | 7 | G | T | A | C |  | A | A | A | A |
| ADP0682 | UI-686 | 7 | T | G | T | T |  | B | B | B | B |
| ADP0683 | IJR | 7 | T | G | T | T |  | B | B | B | B |
| ADP0684 | Majesty | 7 | T | G | T | T |  | B | B | B | B |
| ADP0742 | pur red | 7 | G | T | A | C |  | A | A | A | A |
| ADP0745 | dark pur | 7 | G | T | A | C |  | A | A | A | A |
| ADP0747 | red | 7 | G | T | A | C |  | A | A | A | A |
| ADP0014 | KIANGWE | 7.3 | T | G | T | T |  | B | B | B | B |
| ADP0015 | W6 16495 | 7.3 | G | T | A | C |  | A | A | A | A |
| ADP0027 | Incomparable | 7.3 | G | T | A | C |  | A | A | A | A |
| ADP0031 | RH No. 11 | 7.3 | G | T | A | C |  | A | A | A | A |
| ADP0033 | KIJIVU | 7.3 | T | G | T | T |  | B | B | B | B |
| ADP0044 | KIJIVU | 7.3 | T | G | T | T |  | B | B | B | B |
| ADP0066 | NJANO | 7.3 | T | G | T | T |  | B | B | B | B |
| ADP0102 | Jesca | 7.3 | T | G | T | T |  | B | B | B | B |
| ADP0183 | G 994 | 7.3 | G | T | A | C |  | A | A | A | A |
| ADP0186 | G 1368 | 7.3 | T | G | T | T |  | B | B | B | B |
| ADP0190 | G 1678 | 7.3 | T | G | T | T |  | B | B | B | B |
| ADP0203 | G 4472 | 7.3 | T | G | T | T |  | B | B | B | B |
| ADP0354 | G 22502 | 7.3 | T | G | T | T |  | B | B | B | B |
| ADP0383 | PI 209486 | 7.3 | T | G | T | T |  | B | B | B | B |
| ADP0413 | PI 319595 | 7.3 | T | G | * | T |  | B | B | * | B |
| ADP0472 | PI527537-B | 7.3 | G | T | A | C |  | A | A | A | A |
| ADP0549 | RWR 10 | 7.3 | T | G | T | T |  | B | B | B | B |
| ADP0583 | SAB650 | 7.3 | G | T | A | C |  | A | A | A | A |
| ADP0611 | Pompadour B | 7.3 | T | G | T | T |  | B | B | B | B |
| ADP0620 | UCD0405 | 7.3 | T | G | T | T |  | B | B | B | B |
| ADP0640 | Beluga | 7.3 | T | G | T | T |  | B | B | B | B |
| ADP0674 | UCD 0704 | 7.3 | T | G | T | T |  | B | B | B | B |
| ADP0686 | UCD 707 | 7.3 | T | G | T | T |  | B | B | B | B |
| ADP0743 | d. red | 7.3 | T | G | T | T |  | B | B | B | B |
| ADP0004 | KILOMBERO | 7.7 | T | G | T | T |  | B | B | B | B |
| ADP0021 | MBULAMTWE | 7.7 | G | G | T | T |  | A | B | B | B |
| ADP0026 | Black Wonder | 7.7 | T | G | T | T |  | B | B | B | B |
| ADP0037 | W6 16488 | 7.7 | G | T | A | C |  | A | A | A | A |
| ADP0049 | W6 16546 | 7.7 | T | G | T | T |  | B | B | B | B |
| ADP0078 | W6 16535 | 7.7 | T | G | T | T |  | B | B | B | B |
| ADP0214 | G 5087 | 7.7 | T | G | T | T |  | B | B | B | B |
| ADP0242 | G 9013 | 7.7 | T | G | * | T |  | B | B | * | B |
| ADP0247 | G 9975 | 7.7 | T | G | T | T |  | B | B | B | B |
| ADP0269 | G 13092 | 7.7 | T | G | T | T |  | B | B | B | B |
| ADP0303 | G 17913 | 7.7 | G | T | A | C |  | A | A | A | A |
| ADP0391 | PI 308894 | 7.7 | T | G | * | T |  | B | B | * | B |
| ADP0460 | PI331356-B | 7.7 | T | G | T | T |  | B | B | B | B |
| ADP0464 | PI353534-B | 7.7 | G | T | A | C |  | A | A | A | A |
| ADP0466 | PI449430 | 7.7 | T | G | T | T |  | B | B | B | B |
| ADP0467 | PI209808 | 7.7 | T | G | T | T |  | B | B | B | B |
| ADP0469 | PI527521 | 7.7 | T | G | T | T |  | B | B | B | B |
| ADP0480 | PI209804 | 7.7 | T | G | T | T |  | B | B | B | B |
| ADP0524 | KAT B1 | 7.7 | G | G | T | T |  | A | B | B | B |
| ADP0596 | G16104E | 7.7 | T | G | T | T |  | B | B | B | B |
| ADP0610 | G-122 | 7.7 | T | G | T | T |  | B | B | B | B |
| ADP0613 | 02-385-14 | 7.7 | T | G | T | T |  | B | B | B | B |
| ADP0642 | Taylor Hort | 7.7 | T | G | T | T |  | B | B | B | B |
| ADP0656 | Royal Red | 7.7 | T | G | T | T |  | B | B | B | B |
| ADP0657 | Kardinal | 7.7 | G | T | A | C |  | A | A | A | A |
| ADP0664 | Silver Cloud | 7.7 | T | G | * | T |  | B | B | * | B |
| ADP0668 | Cran-09 | 7.7 | T | G | T | T |  | B | B | B | B |
| ADP0670 | AC Calmont | 7.7 | T | G | T | T |  | B | B | B | B |
| ADP0750 | dark red | 7.7 | T | G | T | T |  | B | B | B | B |
| ADP0010 | CANADA | 8 | T | G | T | T |  | B | B | B | B |
| ADP0013 | KIBUMBULA | 8 | T | G | T | T |  | B | B | B | B |
| ADP0038 | Moono | 8 | T | G | T | T |  | B | B | B | B |
| ADP0067 | NJANO | 8 | G | T | A | C |  | A | A | A | A |
| ADP0180 | G 433 | 8 | T | G | T | T |  | B | B | B | B |
| ADP0220 | G 5625 | 8 | T | G | T | T |  | B | B | B | B |
| ADP0271 | G 13167 | 8 | T | G | T | T |  | B | B | B | B |
| ADP0277 | G 13778 | 8 | T | G | T | T |  | B | B | B | B |
| ADP0280 | G 14440 | 8 | T | G | T | T |  | B | B | B | B |
| ADP0288 | G 16110A | 8 | T | G | T | T |  | B | B | B | B |
| ADP0519 | Katarina, Cela | 8 | T | G | T | T |  | B | B | B | B |
| ADP0543 | G 16157 | 8 | T | G | * | T |  | B | B | * | B |
| ADP0546 | RED CANADIAN WONDER | 8 | T | G | T | T |  | B | B | B | B |
| ADP0557 | COS16 | 8 | T | T | A | C |  | B | A | A | A |
| ADP0569 | MDRK | 8 | T | G | T | T |  | B | B | B | B |
| ADP0577 | SAB620 | 8 | T | G | T | T |  | B | B | B | B |
| ADP0579 | SAB623 | 8 | T | G | T | T |  | B | B | B | B |
| ADP0587 | SAB712 | 8 | T | G | T | T |  | B | B | B | B |
| ADP0591 | VELAZCO LARGO | 8 | T | G | T | T |  | B | B | B | B |
| ADP0604 | 1062-V98 | 8 | T | G | T | T |  | B | B | B | B |
| ADP0638 | Red Hawk | 8 | T | G | T | T |  | B | B | B | B |
| ADP0645 | Lassen | 8 | T | G | T | T |  | B | B | B | B |
| ADP0650 | K-42 | 8 | T | G | * | T |  | B | B | * | B |
| ADP0662 | USCR-9 | 8 | G | T | A | C |  | A | A | A | A |
| ADP0672 | CDRK | 8 | T | G | T | T |  | B | B | B | B |
| ADP0675 | UCD 0801 | 8 | T | G | T | T |  | B | B | B | B |
| ADP0677 | Etna | 8 | T | G | T | T |  | B | B | B | B |
| ADP0679 | Red Rover | 8 | T | G | T | T |  | B | B | B | B |
| ADP0376 | PI 189408 | 8.3 | T | G | T | T |  | B | B | B | B |
| ADP0390 | PI 307808 | 8.3 | T | G | T | T |  | B | B | B | B |
| ADP0417 | PI 451906 | 8.3 | T | G | T | T |  | B | B | B | B |
| ADP0459 | PI331356-C | 8.3 | T | G | T | T |  | B | B | B | B |
| ADP0607 | NY 105 | 8.3 | T | G | T | T |  | B | B | B | B |
| ADP0609 | K-407 | 8.3 | T | G | * | T |  | B | B | * | B |
| ADP0622 | UCD 0701 | 8.3 | T | G | T | T |  | B | B | B | B |
| ADP0634 | UC Red Kidney | 8.3 | T | G | T | T |  | B | B | B | B |
| ADP0659 | USLK-1 | 8.3 | T | G | * | C |  | B | B | * | A |
| ADP0673 | UC Nichols | 8.3 | T | G | T | T |  | B | B | B | B |
| ADP0676 | CELRK | 8.3 | T | G | T | T |  | B | B | B | B |
| ADP0207 | G 4564 | 8.7 | T | G | T | T |  | B | B | B | B |
| ADP0606 | NY 104 | 8.7 | T | G | T | T |  | B | B | B | B |
| ADP0310 | G 18356 | 9 | T | G | T | T |  | B | B | B | B |
| ADP0349 | G 22357 | 9 | T | G | T | T |  | B | B | B | B |
| ADP0598 | Charlevoix | 9 | T | G | T | T |  | B | B | B | B |
| ADP0623 | Drake | 9 | T | G | T | T |  | B | B | * | B |

**Table S9.** Thirteen candidate genes located within the 162-kb mapping interval defined for HB5.1 QTL conferring quantitative resistance to *Pseudomonas syringae* pv. *phaseolicola* Race 6 in the Andean Diversity Panel on Pv05 of the *Phaseolus vulgaris* v1.0 reference genome (G19833).

| Annotated gene ID | Location (Phytozome) | Predicted function^a^ | *Arabidopsis thaliana* homolog (BLASTP) |
| --- | --- | --- | --- |
| Phvul.005G162300 | Chr05: 38726103–38734047 | Leucine-rich repeat (LRR) protein kinase-related | AT1G51800 (< 50%) – Leucine-rich repeat (LRR) protein kinase family protein |
| Phvul.005G162400 | Chr05: 38738787–38743096 | LRR; protein tyrosine kinase; carbohydrate-binding protein of the endoplasmic reticulum (ER) (malectin-like) | AT1G51800 (< 50%) – LRR protein kinase family protein |
| Phvul.005G162500 | Chr05: 38747208–38751502 | LRR); protein tyrosine kinase; carbohydrate-binding protein of the ER (malectin-like) | AT1G51800 (< 50%) – LRR protein kinase family protein |
| Phvul.005G162600 | Chr05: 38756911–38761314 | LRR protein kinase-related | AT1G51800 (< 50%) – LRR protein kinase family protein |
| Phvul.005G162700 | Chr05: 38775063–38775492 | No annotations | None |
| Phvul.005G162800 | Chr05: 38779745–38780205 | No annotations | None |
| Phvul.005G162900 | Chr05: 38789762–38790324 | No annotations | None |
| Phvul.005G163000 | Chr05: 38815045–38820678 | LRR protein kinase-related | AT4G29990 (< 50%) – LRR repeat transmembrane protein kinase protein |
| Phvul.005G163100 | Chr05: 38826801–38831767 | LRR; protein tyrosine kinase; carbohydrate-binding protein of the ER (malectin-like) | AT1G51800 (< 50%) – LRR protein kinase family protein |
| Phvul.005G163200 | Chr05: 38832800–38835402 | LRR protein kinase-related | AT2G19210 (< 50%) – LRR transmembrane protein kinase protein |
| Phvul.005G163300 | Chr05: 38840051–38841174 | No annotations | None |
| Phvul.005G163400 | Chr05: 38865201–38866152 | LRR protein kinase-related | AT2G28970 – LRR protein kinase family protein |
| Phvul.005G163500 | Chr05: 38869901–38874129 | LRR; protein tyrosine kinase; carbohydrate-binding protein of the ER (malectin-like) | AT1G51800 (< 50%) – LRR protein kinase family protein |

^a^Predicted protein functional annotation in the *Phaseolus vulgaris* v1.0 reference genome (genotype G19833) as identified by Phytozome version 11.0 (PANTHER/Pfam/EuKaryotic Orthologous Groups/National Center for Biotechnology Information) (Goodstein *et al.*, 2012).
