# Supplementary material for: Genome-Wide Linkage and Association Mapping of Halo Blight Resistance in Common Bean to Race 6 of the Globally Important Bacterial Pathogen
Source: Front Plant Sci. 2017 Jul 7;8:1170. doi: 10.3389/fpls.2017.01170 (PMC5500643; doi:10.3389/fpls.2017.01170)
Supplement: Supplementary file 2 [file Presentation1.PPTX]

## Slide 1
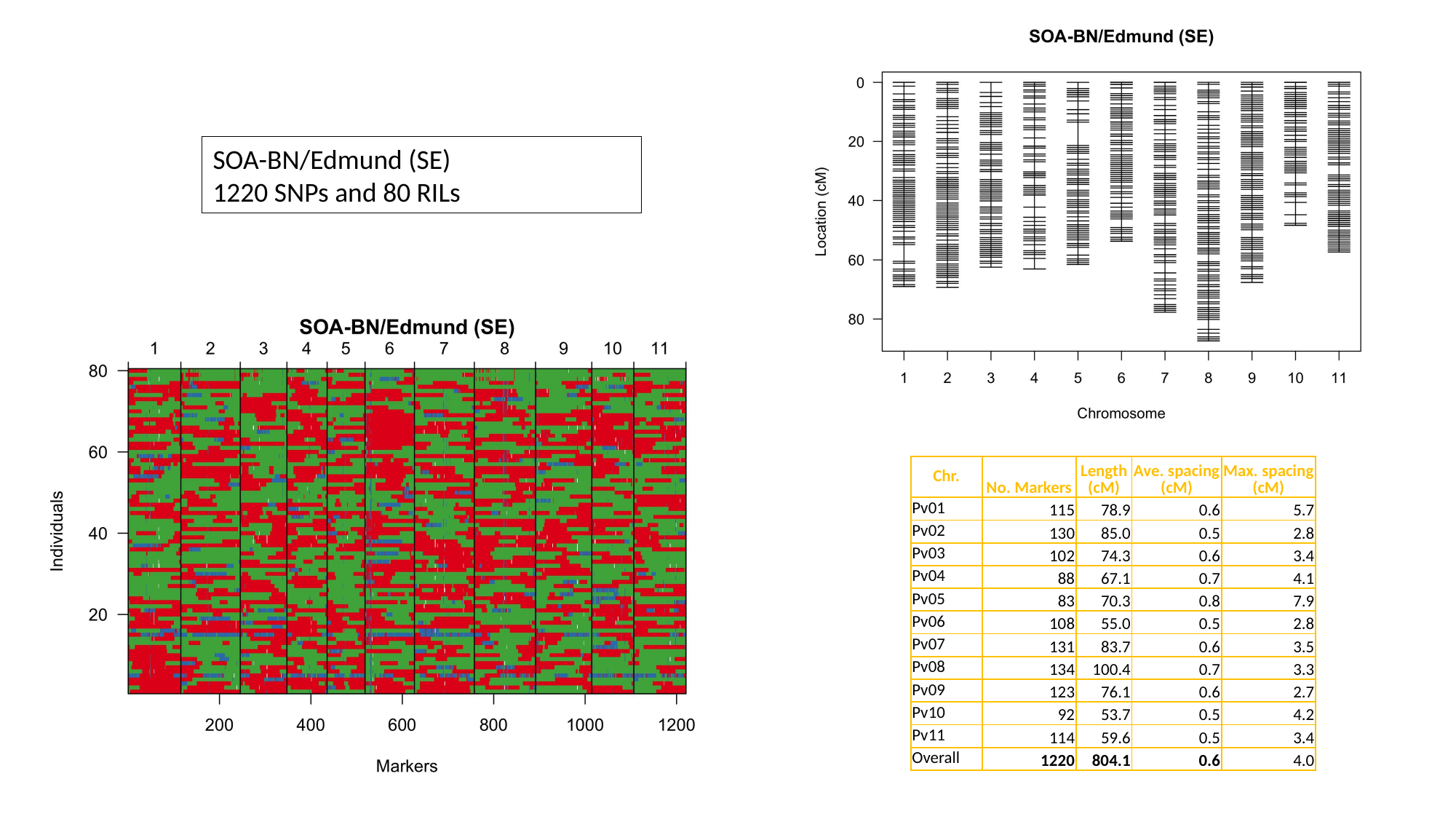

SOA-BN/Edmund (SE)
1220 SNPs and 80 RILs
| Chr. | No. Markers | Length (cM) | Ave. spacing (cM) | Max. spacing (cM) |
| --- | --- | --- | --- | --- |
| Pv01 | 115 | 78.9 | 0.6 | 5.7 |
| Pv02 | 130 | 85.0 | 0.5 | 2.8 |
| Pv03 | 102 | 74.3 | 0.6 | 3.4 |
| Pv04 | 88 | 67.1 | 0.7 | 4.1 |
| Pv05 | 83 | 70.3 | 0.8 | 7.9 |
| Pv06 | 108 | 55.0 | 0.5 | 2.8 |
| Pv07 | 131 | 83.7 | 0.6 | 3.5 |
| Pv08 | 134 | 100.4 | 0.7 | 3.3 |
| Pv09 | 123 | 76.1 | 0.6 | 2.7 |
| Pv10 | 92 | 53.7 | 0.5 | 4.2 |
| Pv11 | 114 | 59.6 | 0.5 | 3.4 |
| Overall | 1220 | 804.1 | 0.6 | 4.0 |

## Slide 2
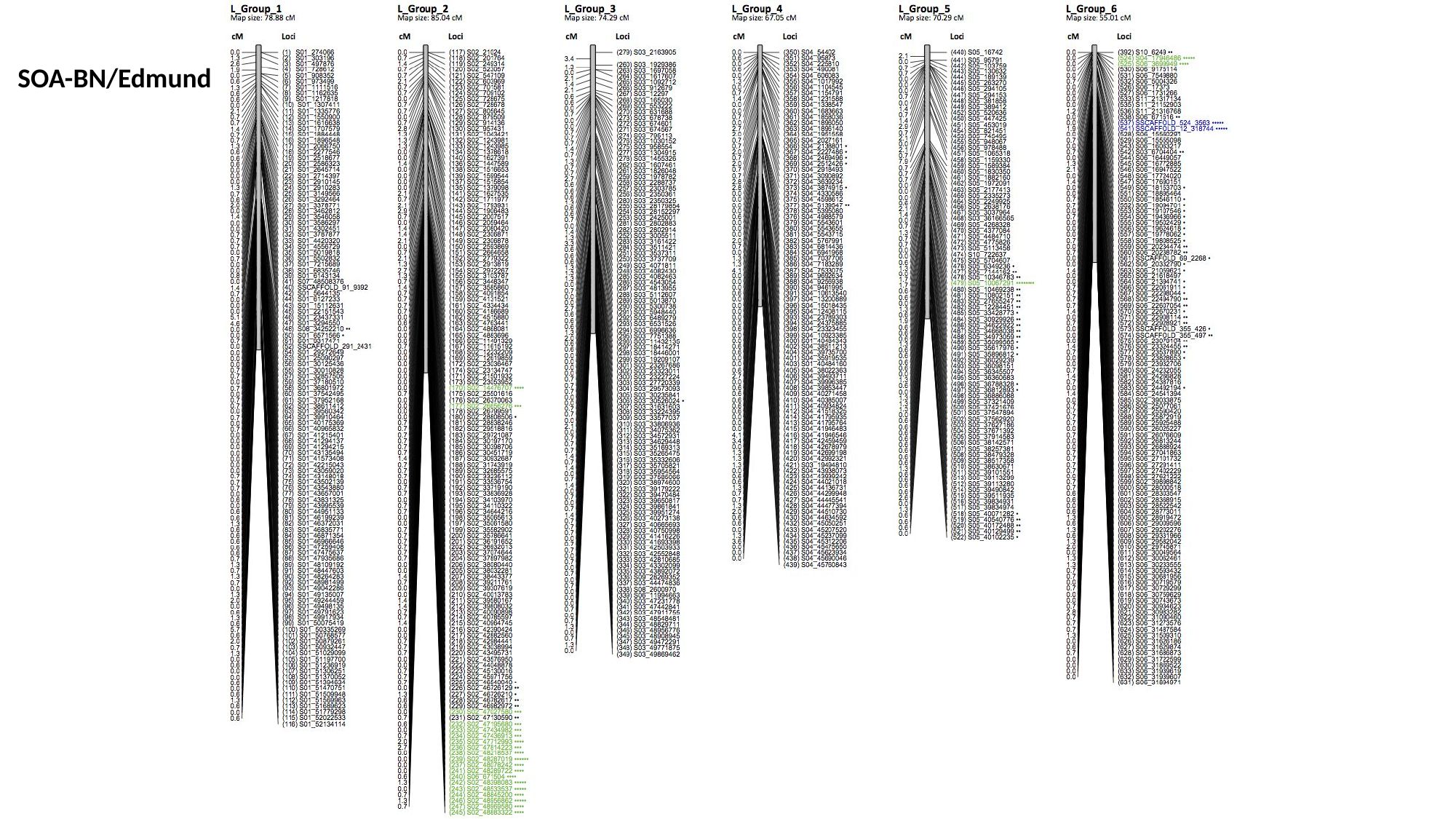

SOA-BN/Edmund

## Slide 3
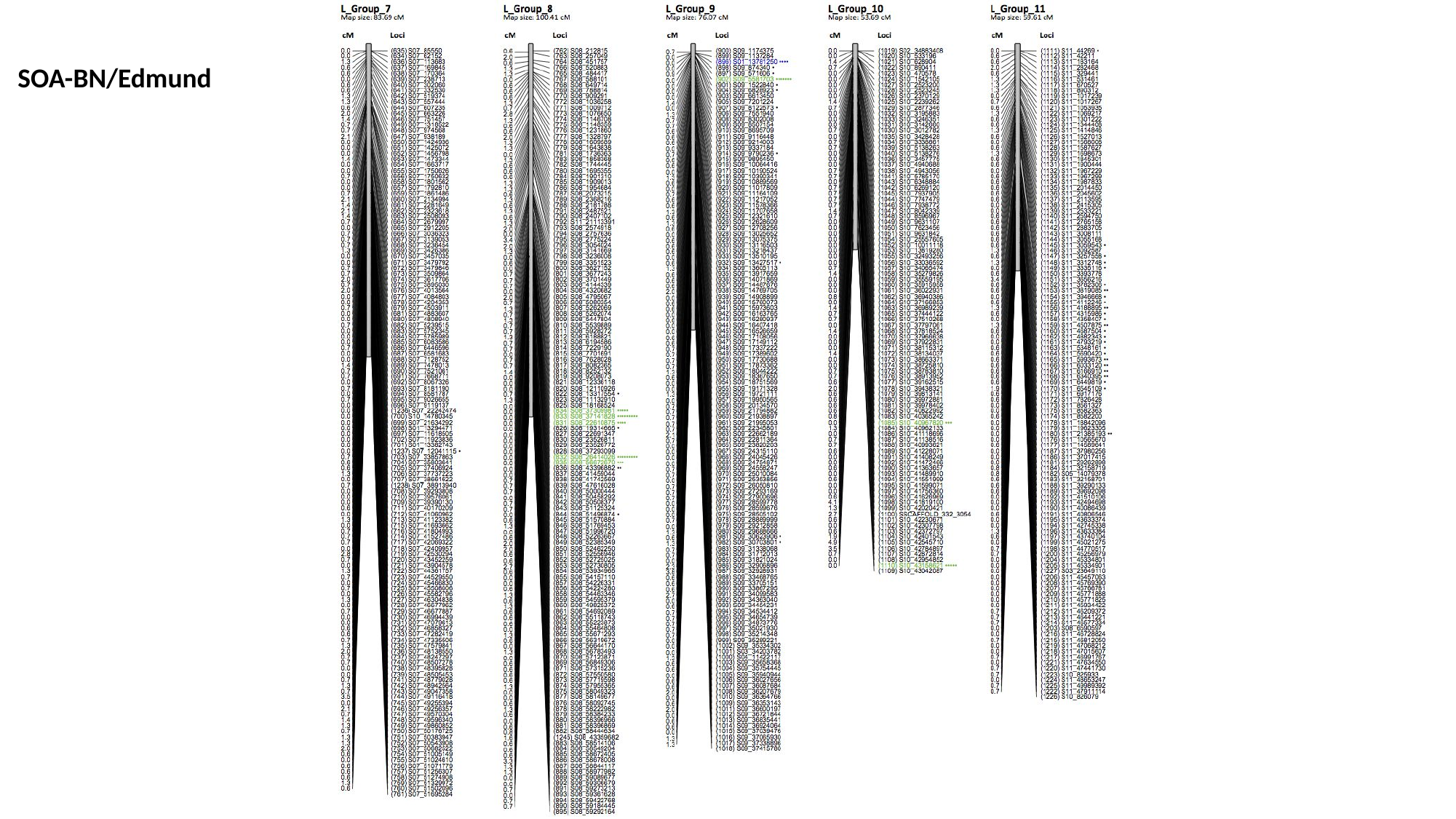

SOA-BN/Edmund

## Slide 4
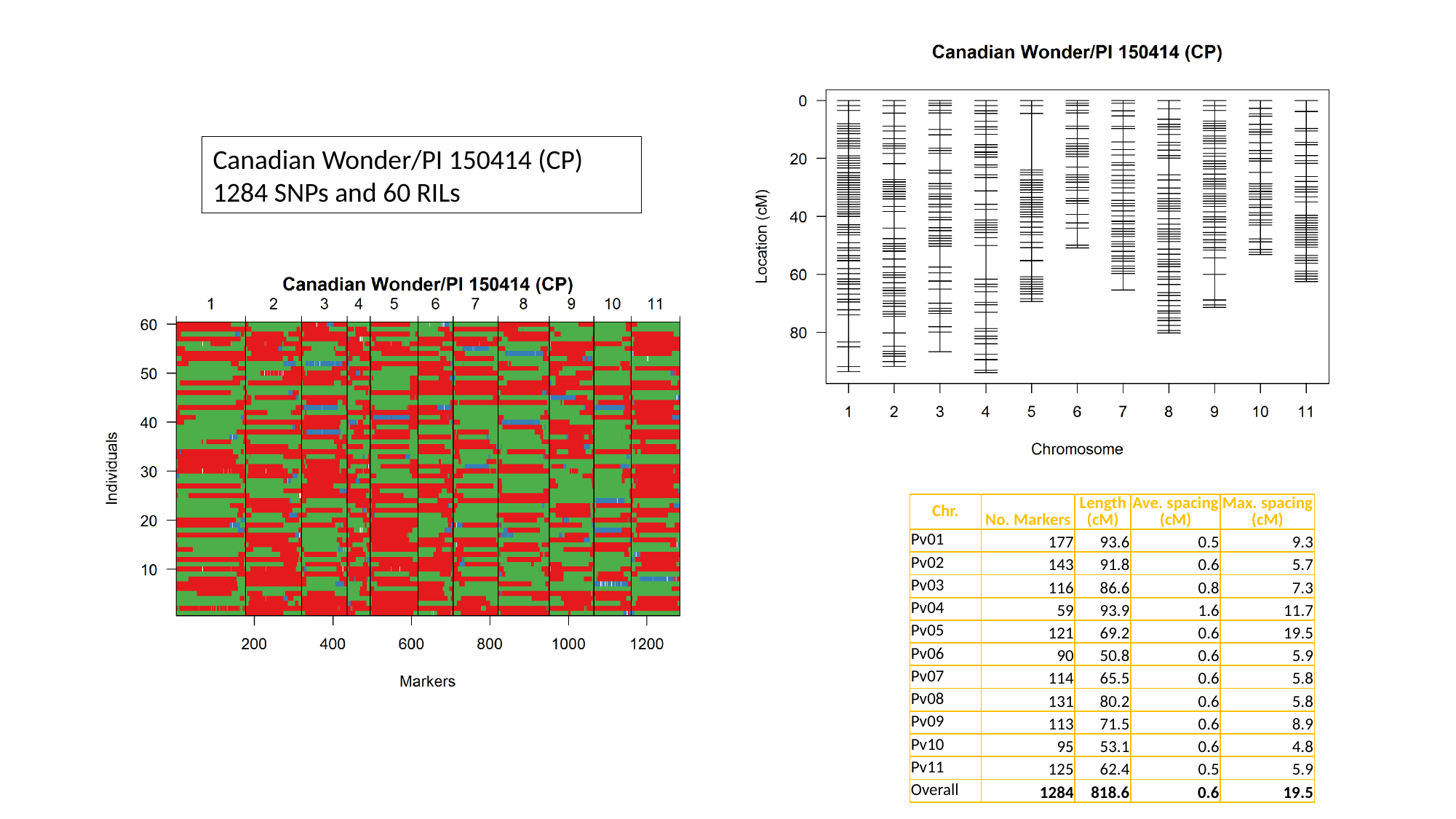

Canadian Wonder/PI 150414 (CP)
1284 SNPs and 60 RILs
| Chr. | No. Markers | Length (cM) | Ave. spacing (cM) | Max. spacing (cM) |
| --- | --- | --- | --- | --- |
| Pv01 | 177 | 93.6 | 0.5 | 9.3 |
| Pv02 | 143 | 91.8 | 0.6 | 5.7 |
| Pv03 | 116 | 86.6 | 0.8 | 7.3 |
| Pv04 | 59 | 93.9 | 1.6 | 11.7 |
| Pv05 | 121 | 69.2 | 0.6 | 19.5 |
| Pv06 | 90 | 50.8 | 0.6 | 5.9 |
| Pv07 | 114 | 65.5 | 0.6 | 5.8 |
| Pv08 | 131 | 80.2 | 0.6 | 5.8 |
| Pv09 | 113 | 71.5 | 0.6 | 8.9 |
| Pv10 | 95 | 53.1 | 0.6 | 4.8 |
| Pv11 | 125 | 62.4 | 0.5 | 5.9 |
| Overall | 1284 | 818.6 | 0.6 | 19.5 |

## Slide 5
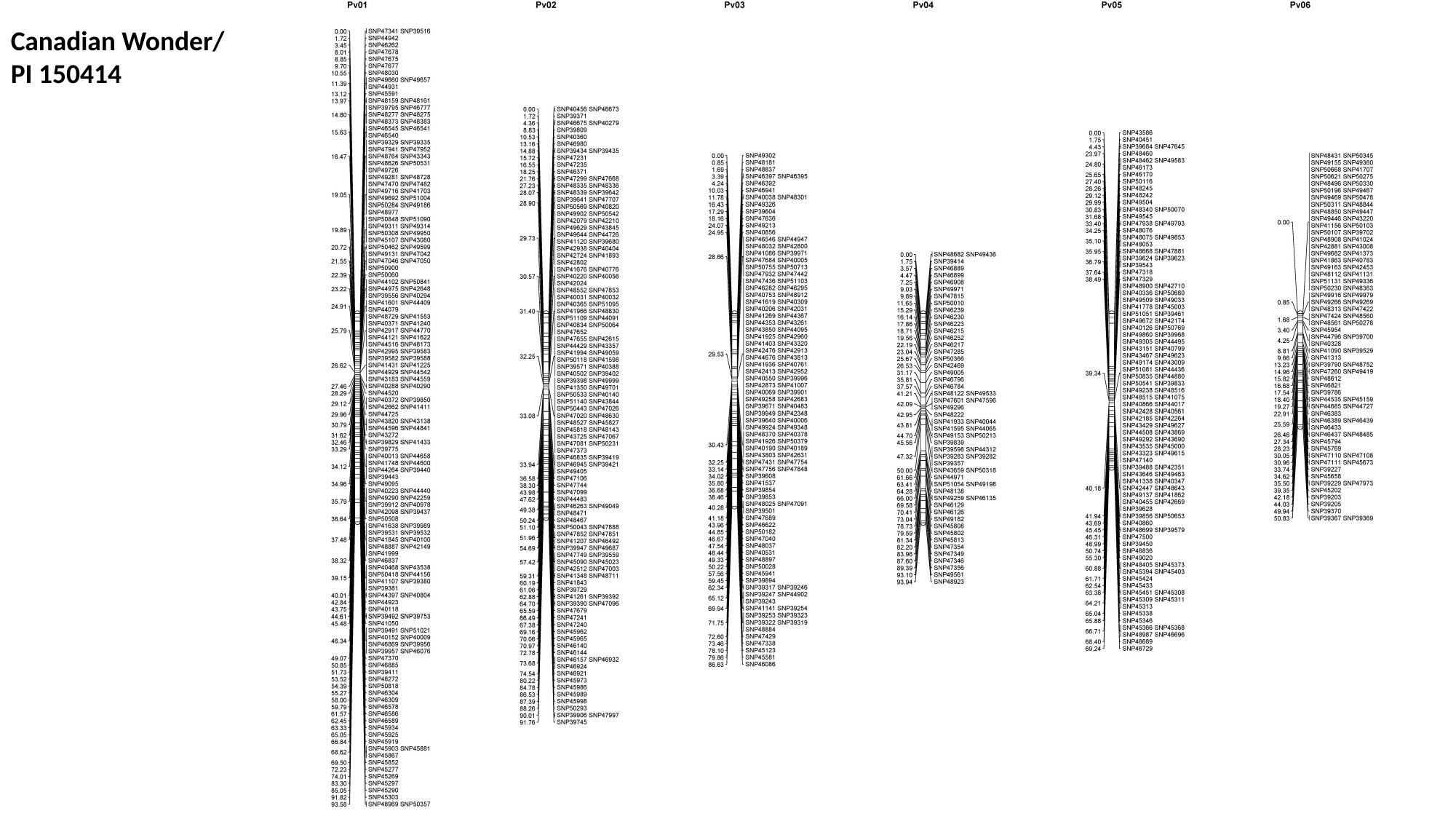

Canadian Wonder/
PI 150414

## Slide 6
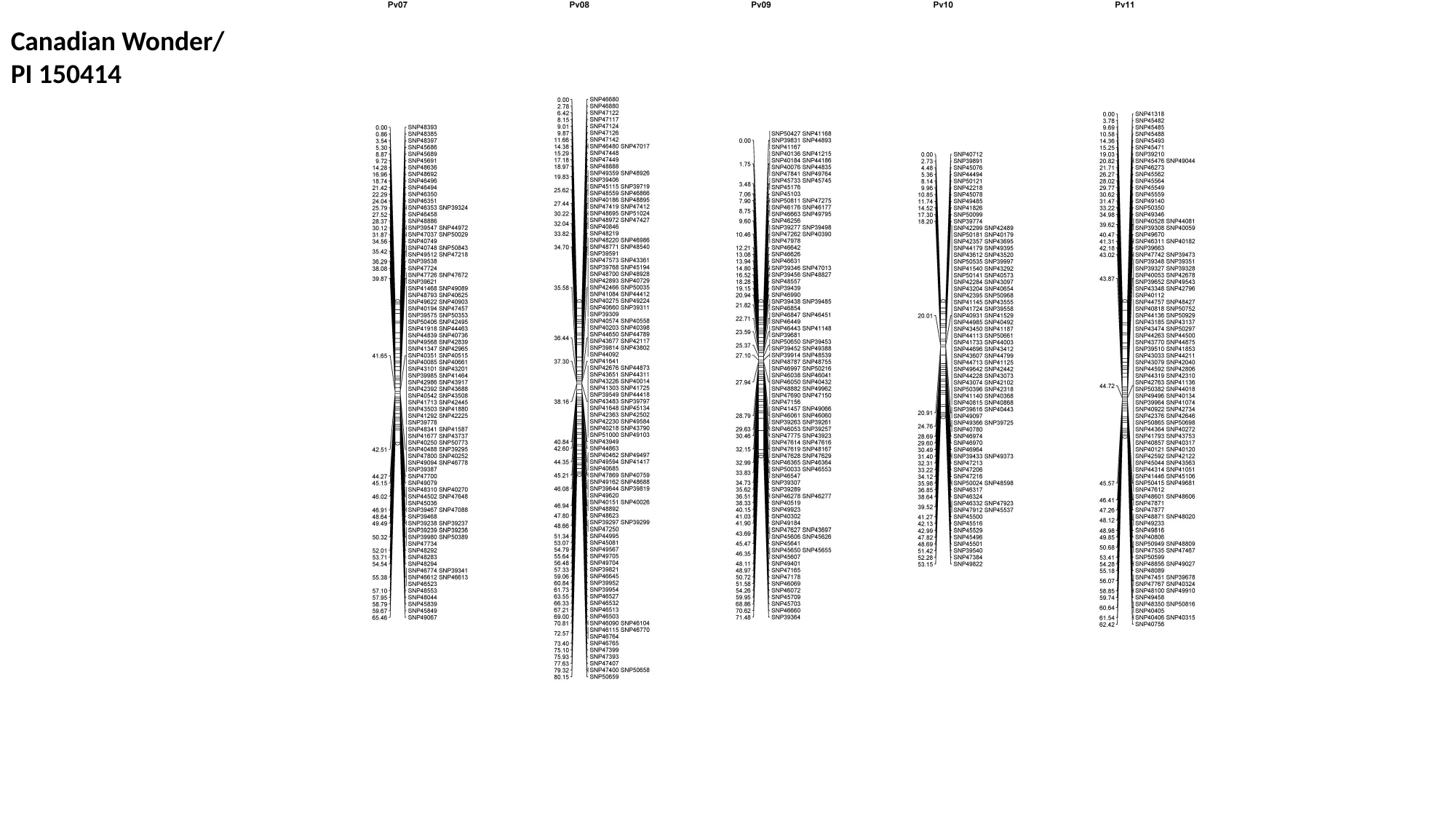

Canadian Wonder/
PI 150414

## Slide 7
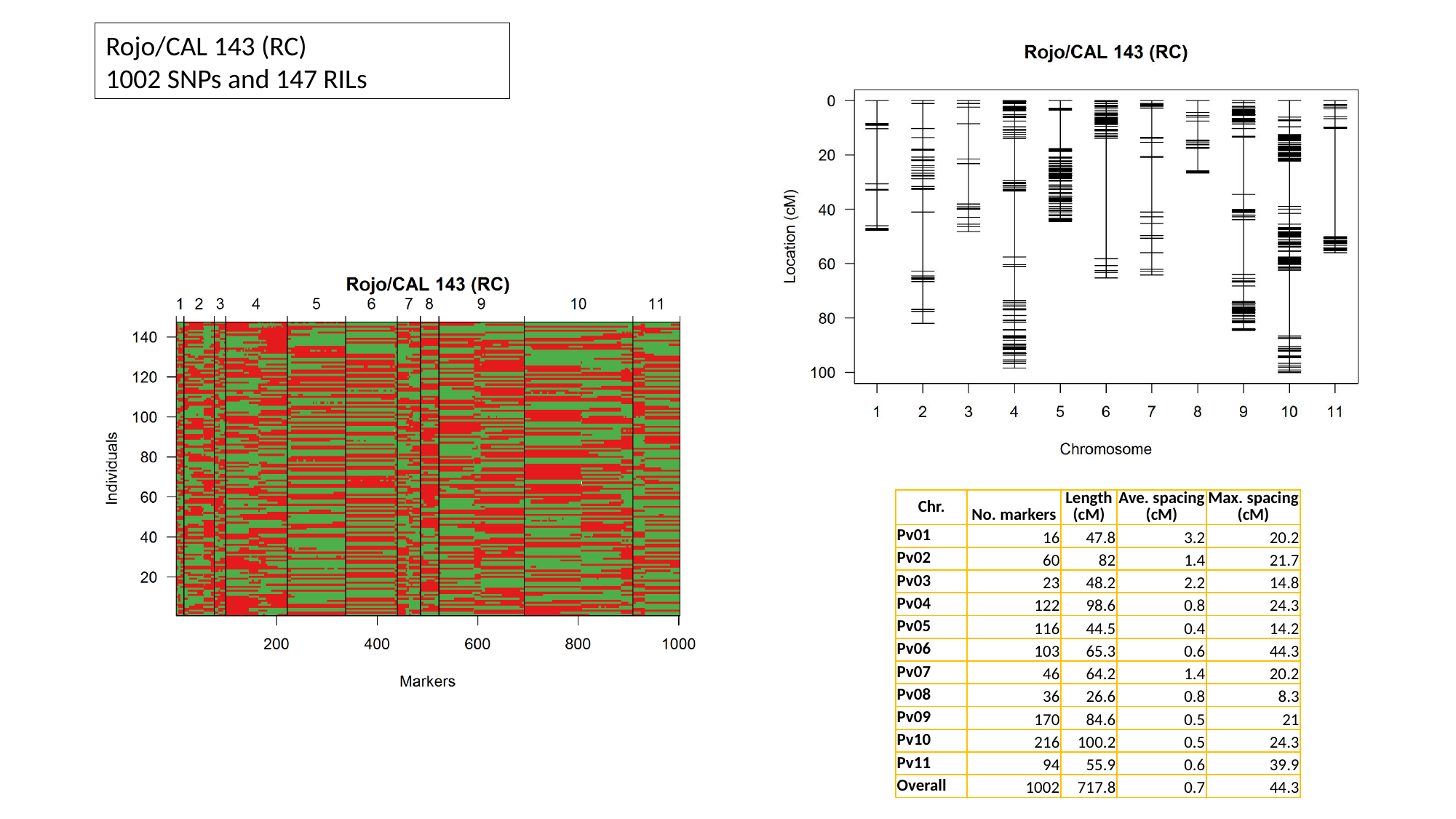

Rojo/CAL 143 (RC)
1002 SNPs and 147 RILs
| Chr. | No. markers | Length (cM) | Ave. spacing (cM) | Max. spacing (cM) |
| --- | --- | --- | --- | --- |
| Pv01 | 16 | 47.8 | 3.2 | 20.2 |
| Pv02 | 60 | 82 | 1.4 | 21.7 |
| Pv03 | 23 | 48.2 | 2.2 | 14.8 |
| Pv04 | 122 | 98.6 | 0.8 | 24.3 |
| Pv05 | 116 | 44.5 | 0.4 | 14.2 |
| Pv06 | 103 | 65.3 | 0.6 | 44.3 |
| Pv07 | 46 | 64.2 | 1.4 | 20.2 |
| Pv08 | 36 | 26.6 | 0.8 | 8.3 |
| Pv09 | 170 | 84.6 | 0.5 | 21 |
| Pv10 | 216 | 100.2 | 0.5 | 24.3 |
| Pv11 | 94 | 55.9 | 0.6 | 39.9 |
| Overall | 1002 | 717.8 | 0.7 | 44.3 |

## Slide 8
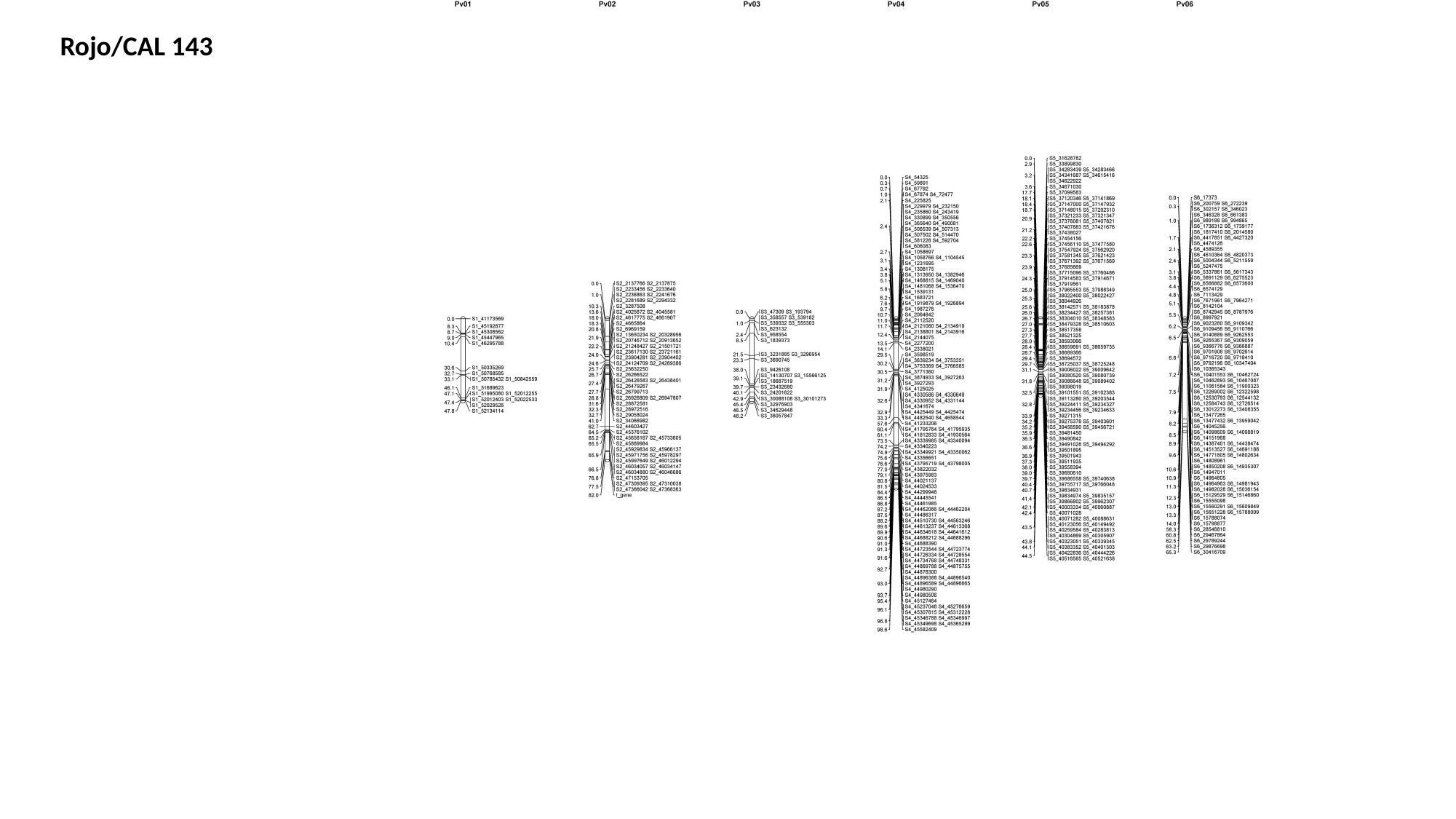

Rojo/CAL 143

## Slide 9
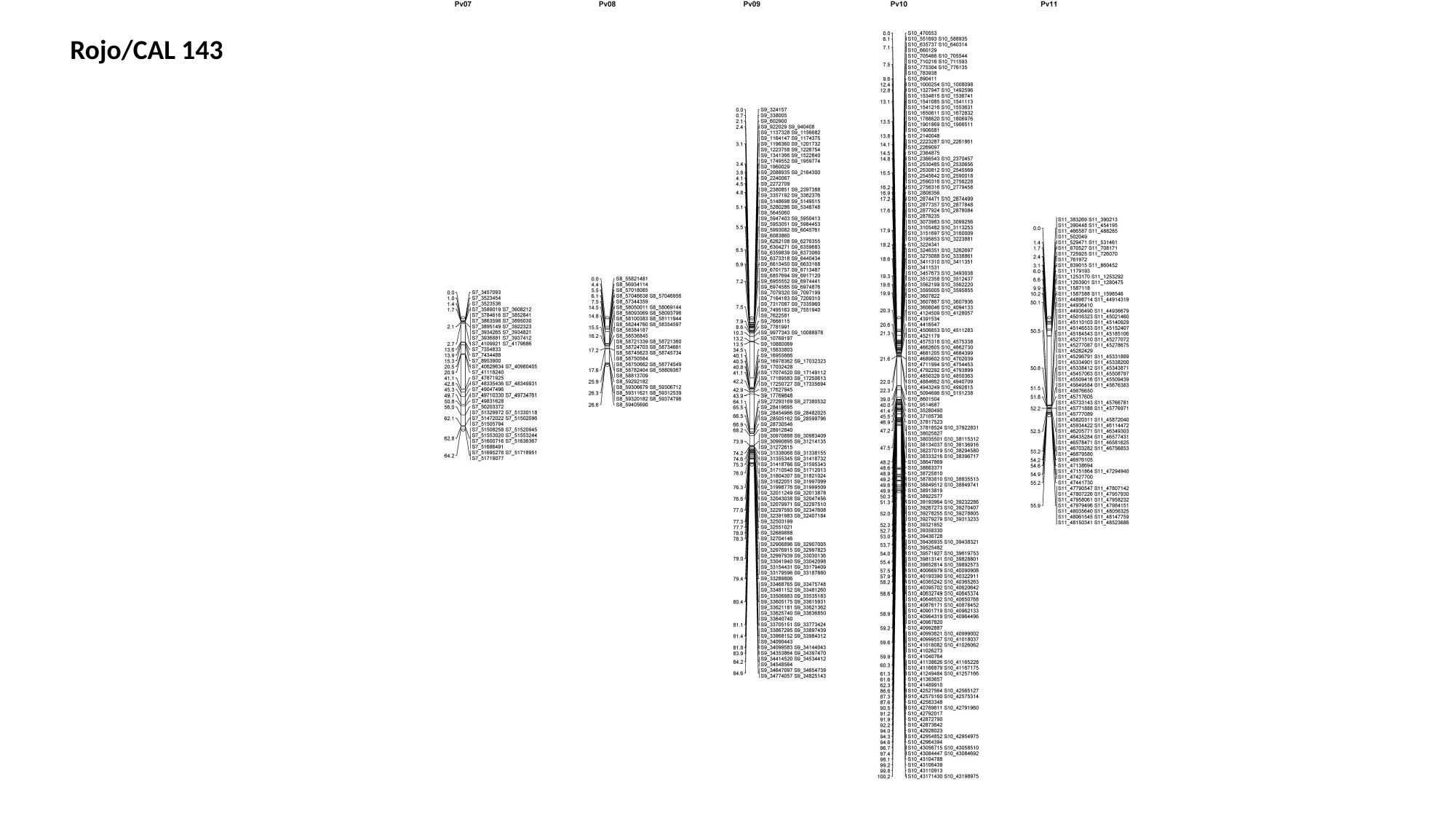

Rojo/CAL 143
